# High-Throughput Mutagenesis and Cross-Complementation Experiments Reveal Substrate Preference and Critical Residues of the Capsule Transporters in *Streptococcus pneumoniae*

**DOI:** 10.1128/mBio.02615-21

**Published:** 2021-11-02

**Authors:** Wan-Zhen Chua, Matthias Maiwald, Kean Lee Chew, Raymond Tzer-Pin Lin, Sanduo Zheng, Lok-To Sham

**Affiliations:** a Infectious Diseases Translational Research Programme and Department of Microbiology and Immunology, National University of Singapore, Singapore; b Department of Pathology and Laboratory Medicine, KK Women’s and Children’s Hospital Singapore; c Duke-NUS Graduate Medical School, National University of Singapore, Singapore; d Department of Laboratory Medicine, National University Hospital, Singapore; e National Public Health Laboratory, Ministry of Health, Singapore; f National Institute of Biological Sciences, Beijing, China; g Tsinghua Institute of Multidisciplinary Biomedical Research, Tsinghua University, Beijing, China; National Cancer Institute

**Keywords:** MOP transporters, *Streptococcus pneumoniae*, capsular polysaccharide, capsule, lipid flippase, transporters

## Abstract

MOP (Multidrug/Oligosaccharidyl-lipid/Polysaccharide) family transporters are found in almost all life forms. They are responsible for transporting lipid-linked precursors across the cell membrane to support the synthesis of various glycoconjugates. While significant progress has been made in elucidating their transport mechanism, how these transporters select their substrates remains unclear. Here, we systematically tested the MOP transporters in the Streptococcus pneumoniae capsule pathway for their ability to translocate noncognate capsule precursors. Sequence similarity cannot predict whether these transporters are interchangeable. We showed that subtle changes in the central aqueous cavity of the transporter are sufficient to accommodate a different cargo. These changes can occur naturally, suggesting a potential mechanism of expanding substrate selectivity. A directed evolution experiment was performed to identify gain-of-function variants that translocate a noncognate cargo. Coupled with a high-throughput mutagenesis and sequencing (Mut-seq) experiment, residues that are functionally important for the capsule transporter were revealed. Lastly, we showed that the expression of a flippase that can transport unfinished precursors resulted in an increased susceptibility to bacitracin and mild cell shape defects, which may be a driving force to maintain transporter specificity.

## INTRODUCTION

**FIG 1:**
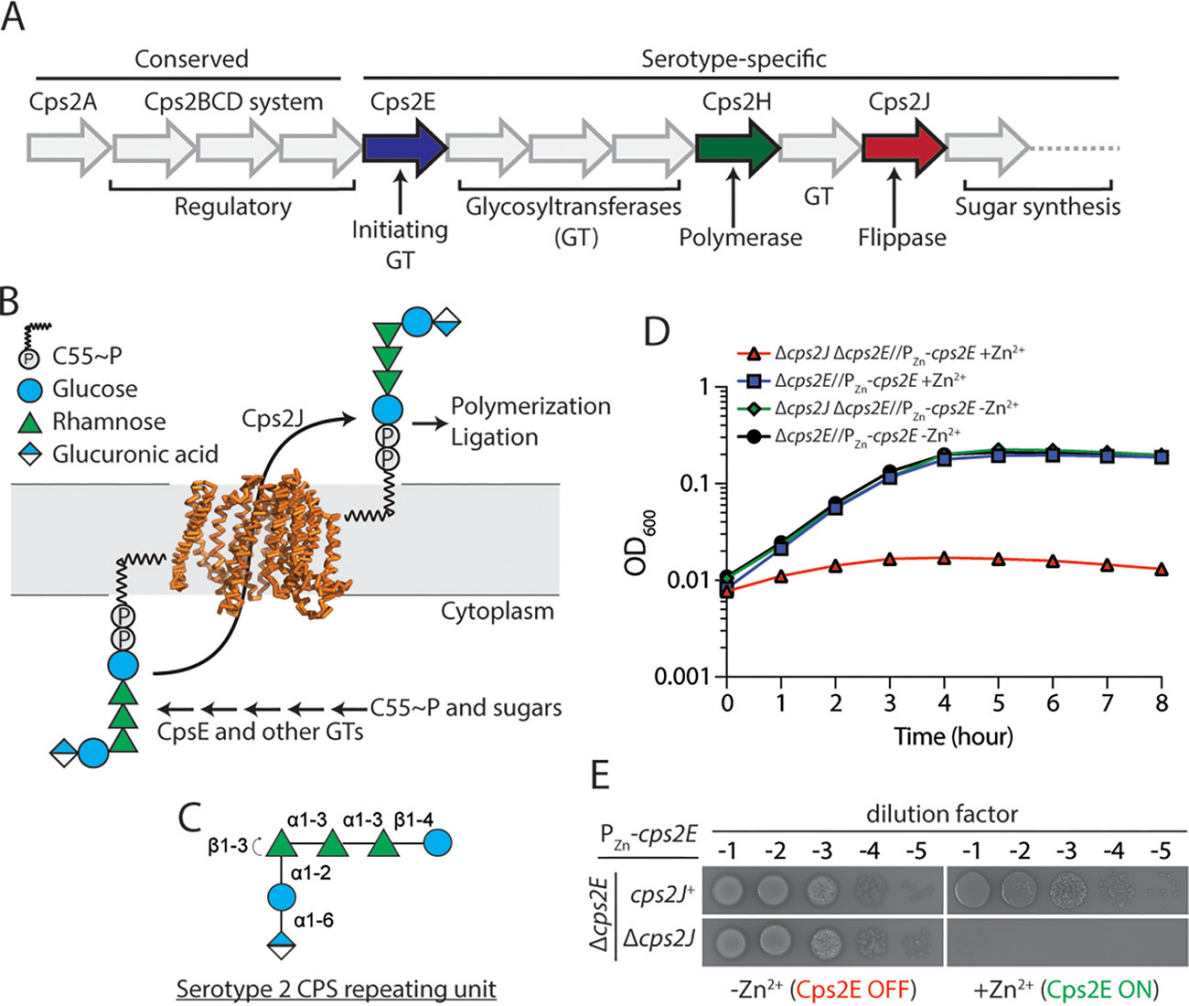
CpsJ transports the CPS repeating units across the cytoplasmic membrane. (A) Genetic organization of the *cps* locus in the serotype 2 strain D39. The first four genes, *cps2ABCD*, are conserved in most of the pneumococcal isolates. The predicted flippase Cps2J and the polymerase Cps2H are highlighted in red and green, respectively. (B) The precursor of the serotype 2 CPS pathway is synthesized by the polyprenol phosphate phosphoglycosyl transferase (PGT) Cps2E and other glycosyltransferases (GTs). Once it is completed, the repeating unit is flipped across the cell membrane presumably by Cps2J. Shown is the structural model of Cps2J generated based on the published MurJ structure (39). (C) A schematic of the serotype 2 CPS repeating unit drawn in the Symbol Nomenclature For Glycans (SNFG) format (66). (D) Depletion of *cps2E* alleviates the lethality caused by *cps2J* deletion. Strains NUS0267 (Δ*cps2E*//P_Zn_-*cps2E*) and NUS0893 (Δ*cps2E* Δ*cps2J*//P_Zn_-*cps2E*) were grown in BHI at 37°C in 5% CO_2_ overnight. Cultures were diluted in BHI with or without ZnCl_2_ and MnCl_2_, and growth was monitored by measuring the optical densities. (E) Cultures of the indicated strains were serially diluted and spotted on blood agar with or without ZnCl_2_ and MnCl_2_ supplement. The plates were incubated at 37°C in 5% CO_2_ overnight before being imaged.

Many pathogenic bacteria are surrounded by a layer of capsular polysaccharide (CPS). This layer has multifaceted roles, such as preventing phagocytosis, blocking complement deposition, avoiding mucociliary clearance, ensuring survival under starvation (1), and facilitating transmission to a new host (2; reviewed in references 3, 4, and 5). Typically, CPSs are synthesized via one of the three pathways: the synthase-dependent pathway, the ATP-binding cassette (ABC) transporter pathway, and the Wzx/Wzy pathway (6). Among them, the Wzx/Wzy pathway is the most common mechanism and is found in nearly half of the bacterial species (7). In this pathway, repeating units of CPS are assembled on a lipid carrier called undecaprenyl phosphate (Und-P). The finished lipid-linked precursor is still at the inner leaflet of the cytoplasmic membrane, which must be transported to the other side of the compartment for further processing (or “flipped”). Since the lipid-linked precursor contains a large hydrophilic glycan motif, spontaneous diffusion is too energetically unfavorable to support the rapid expansion of the cell envelope (8). To facilitate substrate translocation, the Wzx (CpsJ) transporter serves as a conduit to catalyze the topological inversion of the precursors (Fig. 1). After the repeating units are flipped, they are polymerized by Wzy(CpsH) and conjugated to peptidoglycan (PG) (9) or other lipid anchors (10). Completing the CPS pathway releases Und-P, which is recycled for the next round of synthesis.

Streptococcus pneumoniae (pneumococcus) can produce more than a hundred types of structurally distinct CPSs (11, 12). Each strain typically produces one type of CPS, also known as the serotype. The type of CPS determines the serological and surface properties of the cell (13). Since most of the pneumococcal CPS studies were conducted in the serotype 2 strain D39 (3, 12), we selected it as our prototype. To specify which serotype the gene is originated from, for simplicity we designated the homolog of serotype 2 flippase (*cps2J*) in serotype 19F as *cps19FJ*. Except for serotype 3 and 37, all pneumococcal CPSs are synthesized by the Wzx/Wzy mechanism in which the *cps* genes are organized into a single operon located between *dexB* and *aliA* (Fig. 1). The first gene of the serotype 2 *cps* locus, *cps2A*, encodes a LytR/CpsA/Psr (LCP) family protein that presumably conjugates CPS to PG (14–16). Cps2B, Cps2C, and Cps2D form a tyrosine kinase system that regulates CPS synthesis (17). The downstream genes encode other enzymes such as the polyprenol phosphate phosphoglycosyl transferase (PGT) Cps2E, the flippase Cps2J(Wzx), and the polymerase Cps2H(Wzy) (Fig. 1). The genetic organization is similar for other pneumococcal *cps* loci. This arrangement presumably facilitates genetic exchanges, which accelerates serotype replacement and the evolution of new serotypes, thereby contributing to the diversity of pneumococcal CPS (18).

MOP (Multidrug/Oligosaccharidyl-lipid/Polysaccharide) superfamily transporters are found in virtually all life forms. Members of this family include the PG precursor (lipid II) flippase MurJ (19, 20), the teichoic acid precursor flippase TacF (21), and the capsule flippase Cps2J (22). In general, MOP transporters are specific for their substrates, unless they are overexpressed (23) or mutagenized (24). Although structures of several MOP family transporters have been solved (25–29), residues responsible for substrate recognition remain unknown. Previously, we attempted to address this question using a directed evolution approach (24). Capsule flippase variants with relaxed substrate specificity were isolated in Escherichia coli by selecting for mutants that can transport PG precursors. Although these mutations provided some mechanistic insights into flippase specificity, the genetic approach was limited by the number of lipid-linked cargos that could be tested. Since the glycan motifs of PG and the colanic acid capsule precursor share little similarity (24), interpreting the results of the study is relatively challenging.

Here, we harnessed the wealth of genetic and structural information on the pneumococcal CPS to revisit this important biological question. CPS flippases from 82 serotypes were tested for their ability to translocate noncognate serotype 2 and 33B precursors. Overall, most CPS flippases are specific in S. pneumoniae. Strikingly, close homologs of Cps2J could not compensate for the Cps2J function, whereas distant homologs in serogroups 10, 33, and 34 could. This is in contrast to the flippases that could complement *cps33BJ*, for which only close homologs of Cps33BJ could substitute for its function. Site-directed mutagenesis of CpsJ in serogroup 10 revealed residues critical for substrate recognition. To better understand the substrate selectivity of CPS flippases, we isolated Cps23BJ variants that could replace Cps2J. In addition, immutable residues in Cps2J were identified. Lastly, we demonstrated that the expression of a CPS flippase capable of transporting an incomplete precursor resulted in cell shape defects and hypersensitivity to bacitracin, which may explain the selective advantage of substrate selection by MOP transporters.

## RESULTS

### Cps2J depletion was lethal and resulted in cell shape defects.

**FIG 2:**
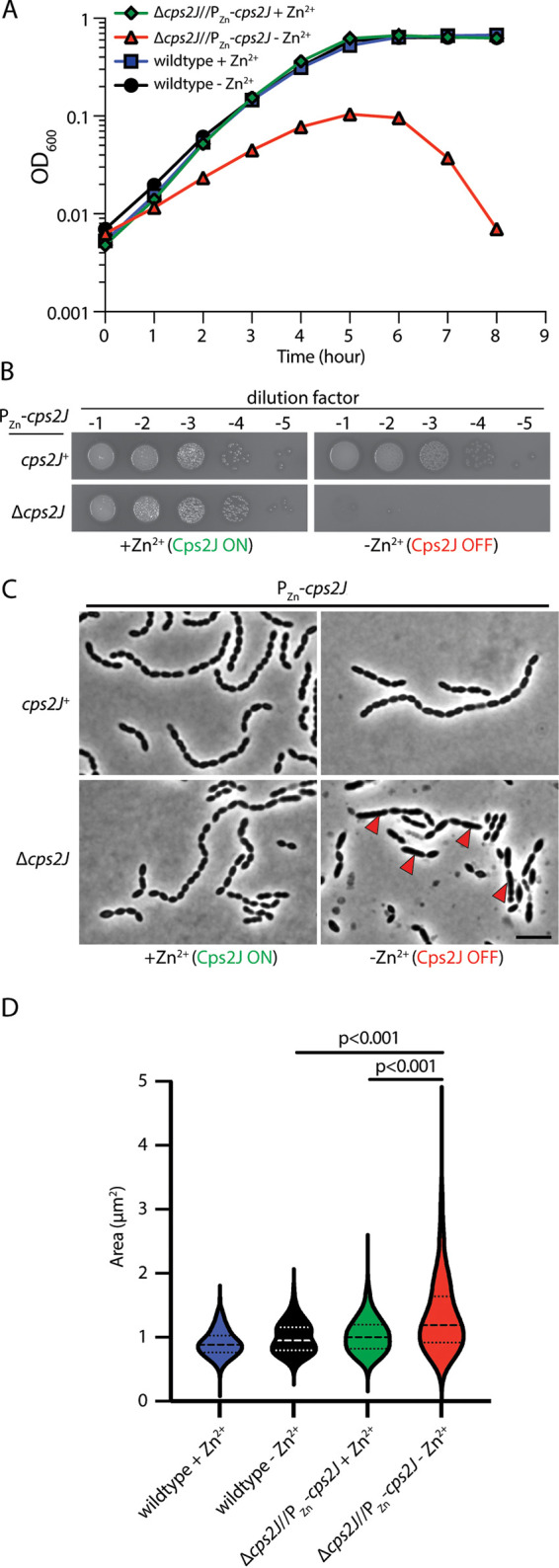
Cps2J is required for growth. (A) Strains NUS0063 (P_Zn_-*cps2J*) and NUS0084 (Δ*cps2J*//P_Zn_-*cps2J*) were grown in BHI supplemented with ZnCl_2_ and MnCl_2_ at 37°C in 5% CO_2_. Cells were washed and diluted in BHI and continued to grow at 37°C. Growth was monitored by measuring the OD_600_ hourly. Shown are representative growth curves from three biological replicates. (B) The same strains were grown in BHI supplemented with ZnCl_2_ and MnCl_2_. Cultures were normalized by their optical densities, serially diluted, and spotted on blood agar with (left) or without (right) ZnCl_2_ and MnCl_2_. (C) Cells of the same strains were grown in BHI with or without ZnCl_2_ and MnCl_2_. Just before the culture started lysing (∼6 h postinoculation), cells were imaged by phase-contrast microscopy, and the images were quantified by MicrobeJ (*n* = 1969) (D). *P* values were computed by using the Mann-Whitney U test. Cells exhibiting cell shape defects are highlighted with red arrows. Scale bar, 5 μm.

Genes involved in the late-stage CPS synthesis are thought to be essential for growth (22, 30). The prevailing model is that their inactivation stalls CPS synthesis, leading to the accumulation of dead-end intermediates. This process subsequently sequesters Und-P and inhibits PG synthesis. In Gram-negative bacteria, the incomplete precursors are also implicated in the disruption of membrane integrity and cause cell shape defects (31, 32). To test whether *cps2J* is required for growth in pneumococcus, we constructed a strain in which *cps2J* expression was under the control of a zinc inducible promoter (P_Zn_). When Cps2J was depleted, cells became elongated and lysed after ∼6 h (Fig. 2). Our result is consistent with the general observation that premature termination of the Wzx/Wzy pathway is lethal because it disrupts Und-P recycling. Indeed, stopping Und-P flux into the CPS pathway by depleting the initiating PGT *cps2E* alleviated the lethality caused by the disruption of *cps2J* (Fig. 1).

### Systematic cross-complementation of Cps2J illustrated substrate preferences.

Except for serotypes 3 and 37, each pneumococcal *cps* locus encodes a dedicated CpsJ flippase. We therefore hypothesized that these flippases are specific, similar to other MOP flippases in Gram-negative bacteria (33–35). To test this, we aligned the CpsJ variants from 93 serotypes. As expected, CPS flippases are remarkably diverse. They can be classified into 17 sequence clusters (see Fig. S1 in the supplemental material), many of which have less than 40% sequence identity to their closest homolog. Interestingly, there is a strong correlation between the primary sequences of CpsJ and the precursors they transport (see Fig. S1). For example, CpsJ variants in serotypes 35F and 47F, as well as in serotypes 35C and 42, are approximately 97% identical, and their substrates are strikingly similar. In contrast, CpsJ variants in serotypes 7F and 7A are different from those in serotypes 7B and 7C, although they belong to the same serogroup. Consistently, the repeating units of serotype 7F and 7A CPSs could be easily distinguished from serotypes 7B and 7C. Assuming the sequence similarity of CpsJ can be used to predict the chemical structures of their cargos, the repeating units of serotype 38 and serogroup 25, as well as serotypes 7B and 40, are likely to be related.

10.1128/mBio.02615-21.2 FIG S1 (A) Diversity of CPS flippases in S. pneumoniae. A phylogenetic tree illustrating the relationship between the CpsJ variants is shown. A selection of CPS repeating units is illustrated on the right. The serotypes in gray were not tested in this study due to technical difficulties in strain construction. The prototypical serotype 2 is highlighted in red. The evolutionary distance shown in the scale bar is the number of amino acid differences per site, which was calculated using the p-distance method (see Materials and Methods). (B) Identification of CpsJ variants that could substitute for Cps2J. Strains derived from NUS0650 (Δ*cps2J*::P-*sacB*-*kan*-*rpsL*^+^//P_Zn_-*cps2J*) harboring various CpsJ variants (see Table S1) were grown, serially diluted, and spotted onto blood plates with or without Zn^2+^/Mn^2+^ as described in the legend of Fig. 2. Plates were imaged ∼16 h after incubation at 37°C in 5% CO_2_. Download FIG S1, PDF file, 0.6 MB. Copyright © 2021 Chua et al. 2021 Chua et al. https://creativecommons.org/licenses/by/4.0/ This content is distributed under the terms of the Creative Commons Attribution 4.0 International license.

**FIG 3:**
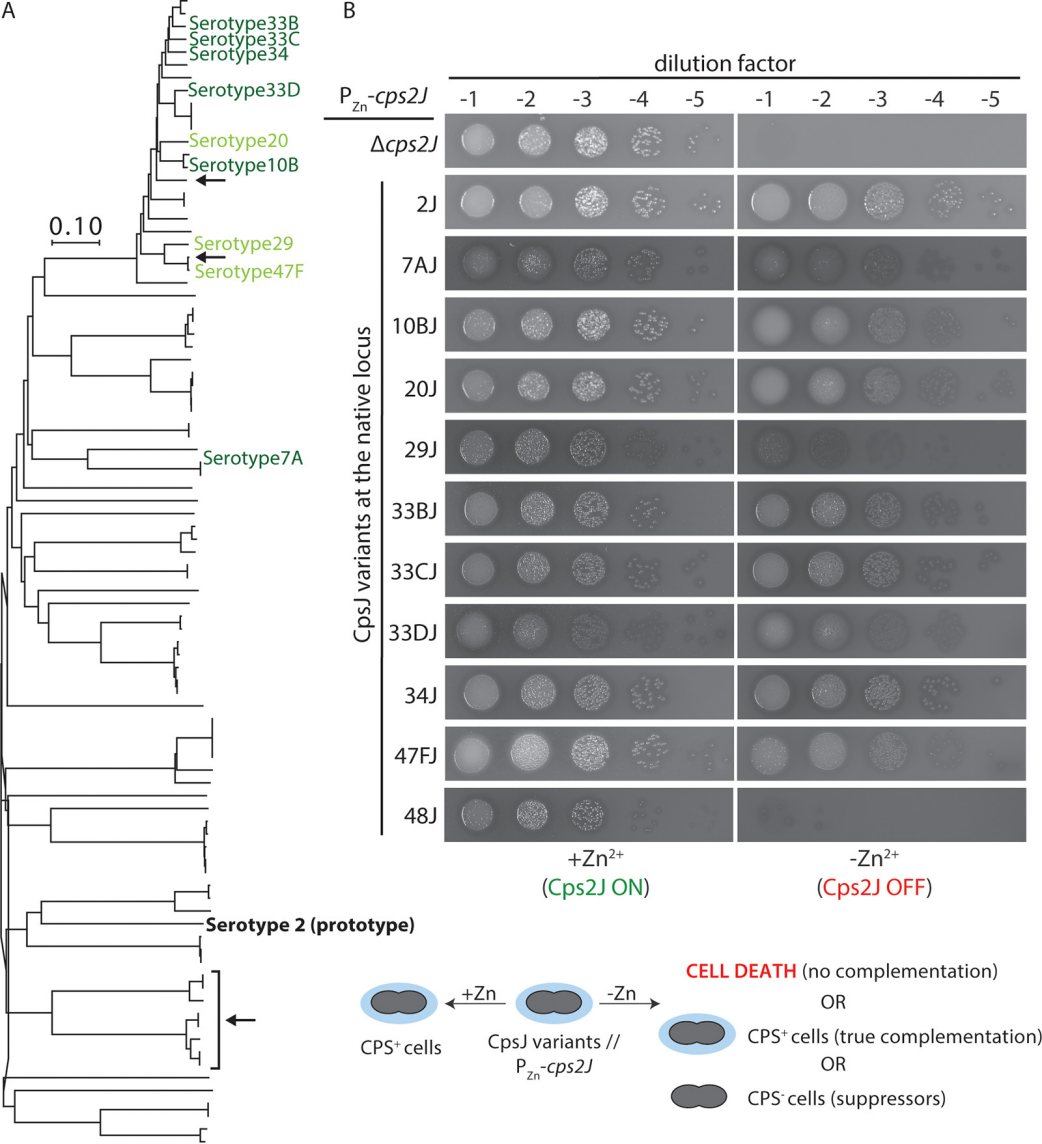
CpsJ variants that could substitute for Cps2J are clustered around serogroup 33. (A) Phylogenetic tree showing the CpsJ variants that can transport the serotype 2 precursor. Serotypes colored dark green produce CpsJ variants that can fully complement *cps2J*, whereas serotypes in light green produce variants that partially complement *cps2J.* The black arrows indicate the serotypes that could not be tested in the study (i.e., serotypes 16A and 35F and serogroup 11). The unit for the scale bar is the number of amino acid differences per site. (B) Cells of strain NUS0650 (*rpsL1* Δ*cps2J*::P-*sacB*-*kan*-*rpsL^+^*//P_Zn_-*cps2J*) and its derivatives expressing different CpsJ variants were grown in BHI with ZnCl_2_ and MnCl_2_. Cultures were diluted and spotted on blood agar as described in the legend of Fig. 2. Plates were incubated at 37°C in 5% CO_2_ overnight before being photographed. Shown are the representative images of the spot dilution assays and the rest are illustrated in Fig. S1B. Complementation of *cps2J* is judged by the formation of smooth colonies on plates without Zn^2+^. Cps48J that could not replace Cps2J is shown here as a negative control for comparison.

To test the interchangeability of CPS flippases, we replaced *cps2J* with the open reading frame of a noncognate *cpsJ*. Clinical isolates of S. pneumoniae were collected from hospitals and research institutes and served as DNA templates (see Table S1). Since Cps2J is essential, we first introduced an inducible copy of *cps2J* at an ectopic locus (P_Zn_-*cps2J^+^*) to maintain cell viability. We then engineered the strains such that *cps2J* at the native locus was replaced by the noncognate *cpsJ* variants. This strategy maintains the gene dosage and prevents artifacts caused by the overexpression of noncognate flippases, because *cpsJ* expression was driven by the native capsule promoter (P_cps_) and ribosomal binding site (RBS_cps_). If the noncognate flippase at the *cps* locus could complement *cps2J* and transport serotype 2 cargo, *cps2J* at the ectopic locus would become dispensable for growth. In total, CpsJ variants from 82 serotypes were tested, and most of them were unable to complement *cps2J* (see Fig. S1). Six CpsJ variants were able to substitute for the Cps2J function (Cps7AJ, Cps10BJ, Cps33BJ, Cps33CJ, Cps33DJ, and Cps34J) and three of them (Cps20J, Cps29J, and Cps47FJ) could only partially complement *cps2J* (Fig. 3). Here, we define partial complementation as the scenario where the mutant carrying the *cpsJ* variant exhibited growth defects when it is the sole copy of CPS flippase in the cell (see below). Unexpectedly, CpsJ variants that could complement *cps2J* are not the closest homologs of Cps2J. Instead, they cluster around serogroup 33 (see Fig. S1). The results indicate that CpsJ is generally specific to its cognate substrate, and the ability to cross-complement cannot be predicted based on their sequence similarity.

10.1128/mBio.02615-21.9 TABLE S1 Bacterial strains (A) and oligonucleotides (B) used in this study. Download Table S1, DOCX file, 0.1 MB. Copyright © 2021 Chua et al. 2021 Chua et al. https://creativecommons.org/licenses/by/4.0/ This content is distributed under the terms of the Creative Commons Attribution 4.0 International license.

We demonstrated that cell viability faithfully reports the flippase activity of Cps2J. Alternative explanations were eliminated by a series of experiments. First, we excluded the possibility that the CpsJ switch mutants contained suppressor mutations in *cps2E* (30). Inactivation of the early *cps* genes (e.g., *cps2E*) prevents Und-P from entering the CPS pathway and alleviates Und-P sequestration. However, the resulting strain could no longer produce CPS. This is not the case because all CpsJ switch mutants remained encapsulated as judged by immunostaining (i.e., the Quellung reaction) (see Fig. S2). Next, we sought to eliminate the possibility that leaky expression of the P_Zn_-*cps2J* cassette might support growth. To test this, the P_Zn_-*cps2J* cassette was replaced with a P-*erm* cassette. The strains remained viable and encapsulated (see Fig. S2), suggesting that these CpsJ variants can indeed complement Cps2J. Finally, inactivation of the noncognate *cpsJ* allele restored zinc dependence for growth, indicating there is no mutation elsewhere in the genome that can account for the survival of the CpsJ switch mutants (see Fig. S2).

10.1128/mBio.02615-21.3 FIG S2 Complementation of *cps2J* is not due to suppressor mutations or leaky expression from the P_Zn_-*cps2J* cassette. (A) The ectopic P_Zn_-*cps2J* cassette was deleted by replacing it with a P*-erm* cassette, which abolishes any residual *cps2J* expression. The resulting strains were grown and spotted on blood agar as described in the legend Fig. 2. Plates were imaged ∼16 h after incubation at 37°C in 5% CO_2_. (B) Deletion of the *cpsJ* variants at the native locus restored the requirement of Zn^2+^ for growth. Strains harboring the indicated *cpsJ* variants (*cpsJ*//P_Zn_-*cps2J*) were transformed with a Δ*cpsJ*::P-*erm* cassette to disrupt the noncognate *cpsJ*. The resulting strains were grown and spotted on blood agar with or without Zn^2+^/Mn^2+^ supplement. Plates were imaged ∼16 h after incubation at 37°C in 5% CO_2_. (C) Strains harboring the noncognate *cpsJ* variants as the only copy of CPS flippase produced the serotype 2 CPS. Cells expressing the indicated *cpsJ* variants were stained with anti-serotype 2 CPS antibodies to visualize the CPS (Quellung reaction). Scale bar, 5 μm. (D) The C-terminal FLAG-tagged CpsJ constructs are functional and generated the same phenotype. Strains harboring the indicated CpsJ-FLAG variants were grown, diluted, and spotted on blood agar with or without Zn^2+^/Mn^2+^. (E) *cps2J* complementation could not be explained by the overproduction of noncognate CpsJ variants. Cells expressing the indicated CpsJ or CpsJ-FLAG variants were grown in BHI medium with Zn^2+^/Mn^2+^. Proteins were extracted, and the FLAG-tagged proteins were detected by immunoblotting with anti-FLAG antibodies. The black arrow indicates the expected size of the corresponding to CpsJ-FLAG constructs. Download FIG S2, PDF file, 0.4 MB. Copyright © 2021 Chua et al. 2021 Chua et al. https://creativecommons.org/licenses/by/4.0/ This content is distributed under the terms of the Creative Commons Attribution 4.0 International license.

It is plausible that Cps2J-complementing flippases are more stable than the others. As shown in other Wzx flippases (23), overexpression of the noncognate CpsJ may support translocation of unrelated substrates. To exclude this possibility, we fused several CpsJ variants (Cps7AJ, Cps10AJ, Cps10BJ, Cps23FJ, Cps33BJ, and Cps48J) with a FLAG tag (DYKDDDDK) at the C terminus and monitored their levels by immunoblotting. FLAG-tagged Cps2J, Cps7AJ, Cps10BJ, and Cps33BJ are functional because they could complement the *cps2J* depletion mutant (see Fig. S2). While the amount of Cps2J was the lowest among the CpsJ variants we tested (see Fig. S2), the ability to substitute Cps2J does not seem to correlate with the protein level of the CpsJ variants (see Fig. S2).

As mentioned above, some CpsJ variants could only partially replace Cps2J. When the P_Zn_-*cps2J* cassette was deleted in mutants harboring these variants (*cps20J*, *cps29J*, and *cps47FJ*), cells exhibited various degrees of growth and cell shape defects (see Fig. S3). To show that they were indeed defective in substrate transport, we quantified the amount of CPS in strains expressing Cps29J (partial complementation) and Cps34J (full complementation) (see Fig. S3). As expected, cells with Cps29J produced ∼40% of the wild-type level of CPS, whereas cells with Cps34J had no significant difference in the amount of CPS (see Fig. S3). Thus, some of these cross-complementing CpsJ variants may be inefficient at transporting serotype 2 substrates.

10.1128/mBio.02615-21.4 FIG S3 Strains expressing CpsJ variants that partially complement *cps2J* exhibited growth and cell shape defects. (A) Strains harboring the indicated CpsJ variants were grown in BHI medium at 37°C in 5% CO_2_ overnight. Cultures were diluted in BHI at a starting OD_600_ of 0.01. (B) Growth was monitored by measuring the OD_600_, and the cells were visualized by phase-contrast microscopy. Scale bar, 5 μm. (C) The area of the cells was quantified by using MicrobeJ (*n* = 2,310). *P* values were computed by using the Mann-Whitney U test. (D) Cps34J and Cps29J can transport the serotype 2 precursor and restore CPS production. Cells expressing the indicated CpsJ variants were grown and biochemically fractionated as described in Materials and Methods. CPS was detected by immunoblotting with anti-serotype 2 CPS antibodies. CW, cell wall fractions; P, protoplast fractions. (E) Quantitation of CPS produced in strains harboring Cps2J, Cps34J, and Cps29J from the immunoblot described in panel D. Plotted are the averages and standard derivations from three biological replicates. *P* values were calculated by using the Student’s *t* test. n.s., not significant. Download FIG S3, PDF file, 0.3 MB. Copyright © 2021 Chua et al. 2021 Chua et al. https://creativecommons.org/licenses/by/4.0/ This content is distributed under the terms of the Creative Commons Attribution 4.0 International license.

### Cps33BJ could not flip PG and teichoic acid precursors.

Since we could not identify any glycan motifs shared by the repeating units of serotype 2 and the cross-complementing serotypes, we hypothesized that the CpsJ variants that could replace Cps2J may have relaxed specificity like Wzk (36). To test this, we investigated whether Cps33BJ could substitute for the lipid II flippase YtgP (a MurJ-like protein) (37) and the teichoic acid precursor flippase TacF (21). We constructed strains in which *cps2J* was replaced by *cps33BJ*, *tacF*, or *ytgP*. In order to maintain cell viability, *cps2J* was expressed ectopically with the P_Zn_ promoter. Given *cps33BJ* could complement *cps2J*, the Zn^2+^ inducer was not required for growth. We then deleted *ytgP* or *tacF* at the native loci. No transformant was obtained in the strain harboring *cps33BJ*, compared to the numerous transformants recovered when the recipient cells were the *cps2J*<>*ytgP* or *cps2J*<>*tacF* mutants (see Table S2A). Unsurprisingly, Cps2J could not substitute for YtgP or TacF, because adding Zn^2+^ to the medium did not allow deletion of these flippases. Together, our results suggest that *cps33BJ* cannot flip PG and teichoic acid precursors to the level that restores growth.

10.1128/mBio.02615-21.10 TABLE S2 CpsJ variants can transport cognate precursors but not structurally distinct lipid-linked precursors of peptidoglycan and teichoic acid. (A) Cps33BJ does not transport lipid-linked peptidoglycan and teichoic acid precursors. (B) Cps10AJ* variants transport the cognate precursor. (C) Cps10BJ does not transport lipid-linked peptidoglycan and teichoic acid precursors. (D) Cps23BJ* variants transport the cognate precursor. (E) Cps23BJ* variants do not transport lipid-linked peptidoglycan and teichoic acid precursors. Download Table S2, DOCX file, 0.02 MB. Copyright © 2021 Chua et al. 2021 Chua et al. https://creativecommons.org/licenses/by/4.0/ This content is distributed under the terms of the Creative Commons Attribution 4.0 International license.

Next, we investigated whether Cps2J could reciprocally flip serotype 33B CPS precursor. The native serotype 2 *cps* locus in strain IU1781 (D39 *rpsL1*) was replaced with the serotype 33B *cps* locus, so that the strain produced a serotype 33B instead of a serotype 2 capsule (38). We then engineered the capsule-switch mutant by introducing an ectopic copy of P_Zn_-*cps33BJ*, followed by deleting *cps33BJ* at the native locus (i.e., NUS0490 [*rpsL1* CPS33B Δ*cps33BJ*//P_Zn_-*cps33BJ*]). The resulting strain now required the Zn^2+^ inducer for growth. *cps2J* was then introduced to the *cps33B* locus, and we tested whether it could complement *cps33BJ* and restore viability. We did not detect complementation of *cps33BJ* by *cps2J* (see Fig. S3), indicating that the cross-complementation event is unlikely caused by the structural similarity between Cps2J and Cps33BJ or their corresponding cargos.

### Cps33BJ could be substituted by its close homologs.

**FIG 4:**
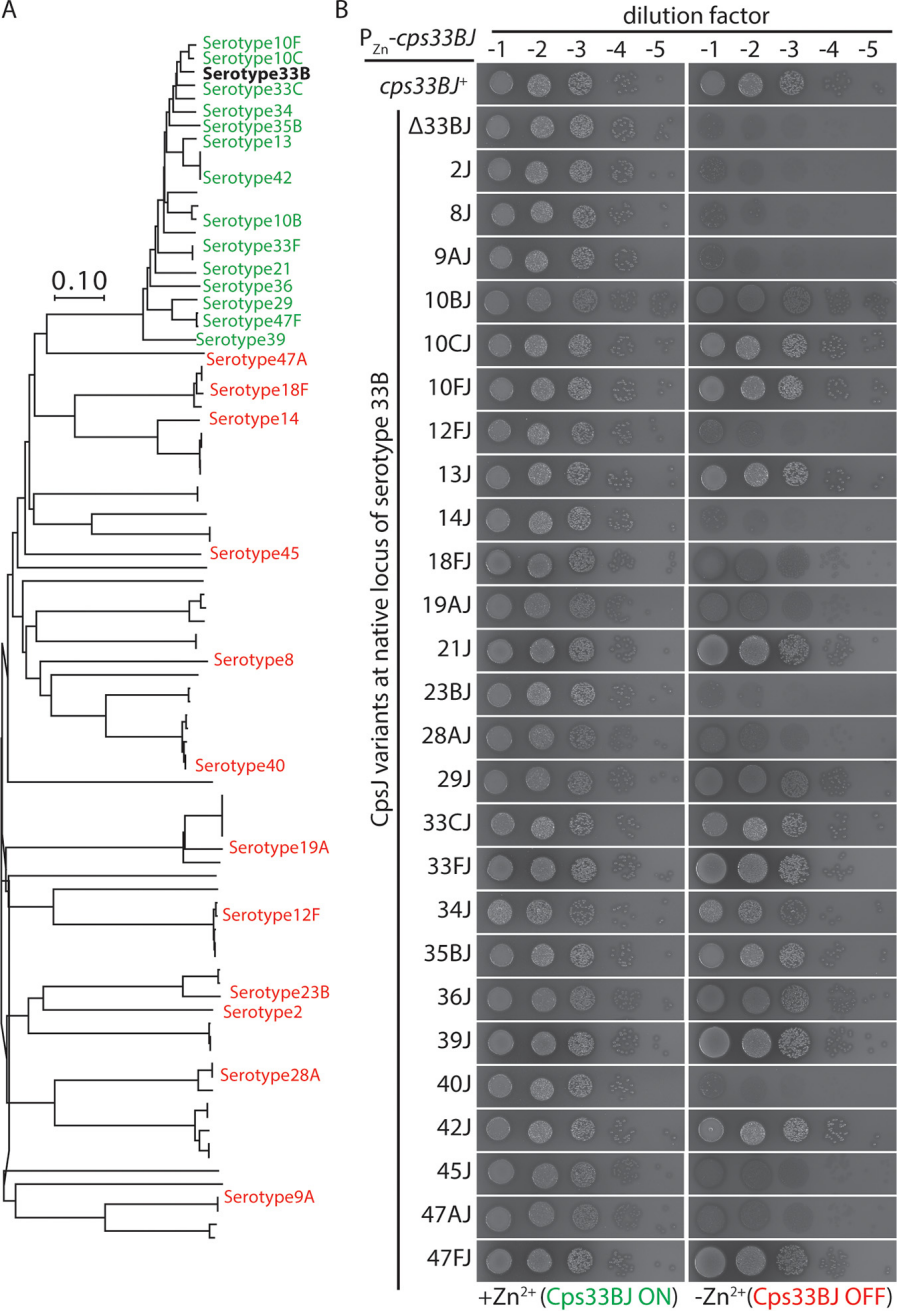
Cps33BJ could be complemented by its close homologs. (A) Representative CpsJ variants from each clade were tested for their ability to transport the serotype 33B precursor. Serotypes highlighted in green represent CpsJ variants that could complement *cps33BJ* (in boldface), whereas serotypes in red indicate CpsJ variants that could not. The unit of the scale bar is the number of amino acid differences per site. (B) Strain NUS1549 (*rpsL1* CPS33B Δ*cps33BJ*::P-*sacB-kan-rpsL^+^*//P_Zn_-*cps33BJ*) and its derivatives harboring different CpsJ variants were grown, serially diluted, and spotted on blood agar plats with or without ZnCl_2_ and MnCl_2_ supplement. Plates were incubated overnight at 37°C in 5% O_2_ before imaging.

We investigated whether Cps33BJ could be replaced by other CpsJ variants, similar to the case of Cps2J. First, we employed the capsule-switch mutant that produced a serotype 33B capsule. An ectopic copy of *cps33BJ* was introduced under the control of a P_Zn_ promoter, followed by replacing *cps33BJ* at the native locus with a noncognate *cpsJ* allele (NUS1549 [*rpsL1* CPS33B Δ*cps33BJ*::P-*sacB-kan-rpsL^+^*//P_Zn_-*cps33BJ*]). As mentioned above, if the CpsJ variant(s) can replace Cps33BJ, the Zn^2+^ inducer would no longer be required for growth. We tested representative CpsJ variants from each clade in Fig. S1, as well as the close homologs of Cps33BJ. Unlike Cps2J, none of the distant Cps33BJ homologs we tested were able to compensate for the Cps33BJ function (Fig. 4). In addition, other than Cps47AJ, CPS flippases that are similar to Cps33BJ could flip Cps33B precursor. These results further support the notion that CPS flippases are generally specific, given CpsJ variants that can substitute for the CPS flippase in another serotype are relatively rare.

### A comparison of Cps10AJ and Cps10BJ revealed residues important for transporting the serotype 2 cargo.

The phylogenetic tree illustrated in Fig. S1 highlighted two interesting CpsJ homologs that might provide insights into the specificity of CPS flippases. Cps10AJ and Cps10BJ differ by five amino acid residues (see Fig. S4), and yet only Cps10BJ could substitute for Cps2J. Structural modeling by I-TASSER suggested Cps10BJ adopts a V-shape conformation, resembling the “inward-open” state of MurJ (28, 29, 39) (see Fig. S4). Assuming MurJ and CpsJ function similarly, Cps10BJ is expected to transition between the inward-open and outward-open states during substrate translocation. If so, mutations that promote the conformational change upon substrate binding may allow CpsJ to translocate a noncognate substrate (40). Based on this assumption, residue F109 in Cps10BJ lies in the putative extracellular gate (see Fig. S4), if changed to valine, may destabilize the inward-open conformation and relax substrate specificity (24). Similarly, the asparagine residue at position 222 of TM8 in Cps10AJ likely favors an inward-open conformation compared to a glutamate residue. We hypothesized that residue H192 is not involved in substrate selection because it is too far away from the putative substrate binding pocket (see Fig. S4). To test these speculations, we performed site-directed mutagenesis to pinpoint the change(s) that allowed Cps10BJ to flip the serotype 2 CPS precursor. As predicted, *cps10BJ^H192Y^* could still complement *cps2J* (see Fig. S4). Changes in the remaining four residues to the equivalent of Cps10AJ (T101I, V109F, N222E, and V356A) abolished serotype 2 precursor transport (see Fig. S4). To evaluate the stability of the variant proteins, we fused the C-terminal end of Cps10BJ with a FLAG tag. The constructs remained functional (see Fig. S2), and the only variant that caused a statistically significant change in the protein level is N222E, which is around 60% of the wild-type level of Cps10BJ (see Fig. S4). Next, we tested whether changing the corresponding residues in Cps10AJ would result in gain-of-function mutations that allowed cross-complementation of *cps2J*. None of the single or double mutants of Cps10AJ could transport the serotype 2 CPS substrate (see Fig. S4). With that, every possible combination has been tested because the triple mutants of Cps10AJ would result in proteins identical to the Cps10BJ single mutants. In addition, we showed that the mutated Cps10AJ still retained the ability to transport its own substrate (see Table S2B), indicating that they are functional flippases. Finally, to examine whether *cps10BJ* could transport structurally distinct substrates, we attempted to delete *ytgP* and *tacF* in strain NUS0550 (*rpsL1 cps2J<>cps10BJ*//P_Zn_-*cps2J*). No transformant could be recovered regardless of Zn^2+^ supplementation (see Table S2C), demonstrating that Cps10BJ could not appreciably transport PG and teichoic acid precursors. In conclusion, the four residues near the proposed central aqueous cavity are involved in the transportation of the noncognate serotype 2 precursor.

10.1128/mBio.02615-21.5 FIG S4 Cps10AJ and Cps10BJ are similar and the identification of residues that are required for Cps10BJ to complement *cps2J*. (A) Sequences of Cps10AJ and Cps10BJ were aligned using Clustal Omega. The black arrows and red letters denote the differences between the two proteins. Apart from the five amino acid residues, two silent mutations are located at positions 96 and 165, respectively. The changes in nucleotide sequence are tabulated in panel B. (C) Strains harboring the P_Zn_-*cps2J* cassette and the indicated *cps10AJ* or *cps10BJ* alleles were grown in BHI with Zn^2+^/Mn^2+^, serially diluted, and spotted onto blood agar plates with or without Zn^2+^/Mn^2+^. The plates were incubated overnight at 37°C in 5% CO_2_ before imaging. (D) Structure model of Cps10BJ predicted by I-TASSER. Residues that are different in Cps10AJ are highlighted with colored sticks. (E) The Cps10BJ variants are stable. Cells expressing the indicated CpsJ or CpsJ-FLAG variants were grown in BHI medium with Zn^2+^/Mn^2+^. Proteins were extracted, and the FLAG-tagged proteins were detected by immunoblotting with anti-FLAG antibodies. The black arrow indicates the expected size of the corresponding CpsJ-FLAG variants. (F) Quantification of the CpsJ variants in strains indicated in panel E. Plotted are the averages and standard deviations of three biological replicates. *P* values were calculated by using the Student’s *t* test. n.s., not significant. Download FIG S4, PDF file, 0.4 MB. Copyright © 2021 Chua et al. 2021 Chua et al. https://creativecommons.org/licenses/by/4.0/ This content is distributed under the terms of the Creative Commons Attribution 4.0 International license.

### Mutagenized Cps23BJ variants could complement *cps2J*.

To investigate why close homologs of Cps2J such as Cps23BJ could not transport serotype 2 substrate, we isolated gain-of-function variants of Cps23BJ that could replace Cps2J (see Fig. S1). Our initial screening strategy was to replace *cps2J* with a mutagenized *cps23BJ* allele while maintaining cell viability by ectopically expressed *cps2J*. However, despite numerous attempts, we could not achieve a sufficiently high transformation efficiency to support this screening strategy. To overcome this problem, we constructed a strain in which *cps2E* expression was controlled by the P_Zn_ promoter. Inactivation of *cps2J* was tolerated, unless Zn^2+^ was added to the medium to initiate CPS synthesis (Fig. 1). With this strain, *cps2J* at the native locus could be readily replaced by a mutagenized copy of *cps23BJ.* Approximately 15,000 transformants were obtained, pooled, and selected on Zn^2+^ supplemented blood plates. Unencapsulated suppressor mutants were discarded by screening ∼100 survivors with immunostaining using anti-CPS antibodies. Isolates that maintained serotype 2 capsule production were sequenced (72 in total). Among them, 17 unique Cps23BJ variants were identified (see Data Set S1 in the supplemental material). The amino acid changes clustered around the predicted extracellular region of the transporter (see Fig. S5), strikingly similar to the WzxC variants reported to have expanded specificity (24). To ensure that the causative mutations were located in *cps23BJ*, we transformed the *cps23BJ* alleles back to the parent strain (NUS0893 [*rpsL1* Δ*cps2E* Δ*cps2J*::P-*sacB*-*kan*-*rpsL^+^*//P_Zn_-*cps2E*]), and the resulting strains retained the phenotype (see Fig. S5). For simplicity, the Cps23BJ variants that can replace Cps2J are here collectively referred to as Cps23BJ*.

10.1128/mBio.02615-21.1 DATA SET S1 (A) List of gain-of-function Cps23BJ variants. (B) High-throughput mutagenesis and sequencing (Mut-seq) data of Cps2J. Download Data Set S1, XLSX file, 0.7 MB. Copyright © 2021 Chua et al. 2021 Chua et al. https://creativecommons.org/licenses/by/4.0/ This content is distributed under the terms of the Creative Commons Attribution 4.0 International license.

10.1128/mBio.02615-21.6 FIG S5 (A) Cps23BJ variants with relaxed substrate specificity. Derivatives of strain NUS0893 (Δ*cps2E* Δ*cps2J*//P_Zn_-*cps2E*) harboring the indicated cps23BJ* alleles were grown and spotted onto blood agar with or without Zn^2+^/Mn^2+^. Plates were photographed after incubation at 37°C in 5% CO_2_ overnight. (B) When *cps2E* expression was restored to the wild-type level, some Cps23BJ* variants could no longer substitute for Cps2J. Δ*cps2J* strains harboring the P_Zn_-*cps2J* cassette, and the indicated cps23BJ* alleles were grown and spotted on blood agar with or without Zn^2+^/Mn^2+^. Plates were photographed after incubation at 37°C in 5% CO_2_ overnight. (C) Amino acid changes that expand substrate specificity of Cps23BJ. A structural model of Cps23BJ generated by I-TASSER is shown. The N- and C-lobes of Cps23BJ are highlighted in blue and green, respectively. Residues identified in the genetic screen that alter substrate specificity are shown as purple sticks. Download FIG S5, PDF file, 0.5 MB. Copyright © 2021 Chua et al. 2021 Chua et al. https://creativecommons.org/licenses/by/4.0/ This content is distributed under the terms of the Creative Commons Attribution 4.0 International license.

We noticed that strains harboring Cps23BJ* in place of Cps2J exhibited growth defects and formed smaller colonies (see Fig. S5). Mild cell shape defects were detected under phase-contrast microscopy after *cps2E* expression was induced (see Fig. S6). This implied that Cps23BJ* variants could not completely replace Cps2J. If so, the amount of CPS produced in these strains should have been reduced. To confirm this, we quantified the CPS in these strains by immunoblotting. As expected, CPS produced in the Cps23BJ* strains were between ≈12% to ≈40% of the wild-type level (see Fig. S6). Since strain NUS0267 (*rpsL1* Δ*cps2E*//P_Zn_-*cps2E*) produced slightly lesser CPS than the wild-type strain, even it is *cps2J*^+^, we wondered whether Cps23BJ* could support growth when the CPS expression was restored to the wild-type level. We therefore introduced cps23BJ* into the native *cps* locus of strain NUS0650 (*rpsL1* Δ*cps2J*::P-*sacB-kan-rpsL*^+^//P_Zn_-*cps2J*) by allelic exchange. Upon *cps2J* depletion, 11 of the 17 cps23BJ* mutants could no longer support grow (see Fig. S5). We also demonstrated that the cps23BJ* alleles remained capable of transporting the serotype 23B substrate because they complemented *cps23BJ* in a capsule-switch mutant (see Table S2D). Lastly, to test whether the Cps23BJ* proteins could flip PG and teichoic acid precursors, *tacF* or *ytgP* was inactivated in strains harboring cps23BJ* alleles, but no viable transformant could be recovered (see Table S2E). We conclude that Cps23BJ* variants could be isolated, although many of which can only partially complement *cps2J* and none can compensate for the function of *tacF* and *ytgP*.

10.1128/mBio.02615-21.7 FIG S6 Cells expressing cps23BJ* exhibited morphological defects and produced less CPS compared to the wild-type cells when Zn^2+^ was added to the medium. (A) Strains derived from NUS0893 (Δ*cps2E* Δ*cps2J*//P_Zn_-*cps2E*) harboring the indicated *cps23BJ* alleles were grown in BHI medium with or without Zn^2+^/Mn^2+^ supplement. Cells were visualized after 6 h of induction by using phase-contrast microscopy. Scale bar, 2 μm. Cultures described in panel A were normalized by their optical densities, harvested by centrifugation, and lysed. The CPS recovered was detected by immunoblotting with anti-serotype 2 antibodies (B) and quantified (C). *P* values were computed using the Student’s *t* test. n.s., not significant. (D) Docking of the serotype 2 CPS precursor to the structural models of Cps2J, Cps10AJ, Cps10BJ, Cps23BJ, and Cps33BJ. Oligosaccharides of the indicated serotypes were retrieved from the Bacterial Carbohydrate Structure Database. Structural models of CpsJ were generated by I-TASSER. The docking experiments and the calculation of binding energies were performed using AutoDock Vina. Download FIG S6, PDF file, 0.3 MB. Copyright © 2021 Chua et al. 2021 Chua et al. https://creativecommons.org/licenses/by/4.0/ This content is distributed under the terms of the Creative Commons Attribution 4.0 International license.

### Essential residues of Cps2J are located in the putative central solvent-exposed cavity.

**FIG 5:**
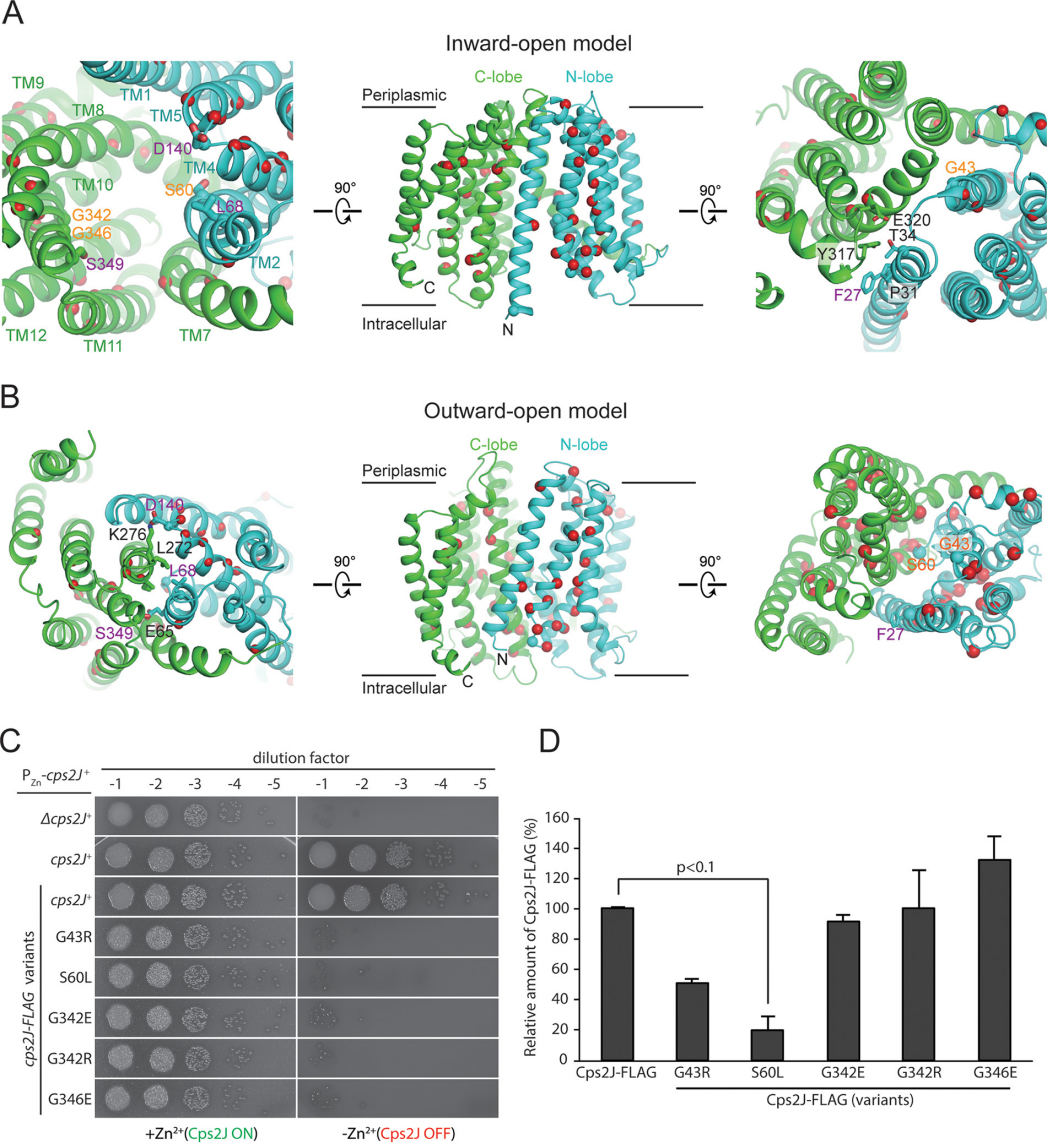
Mapping of the essential residues identified by Mut-seq on the Cps2J model. (A) Ribbon representation of the inward-open model of Cps2J in three different orientations. The N-lobe and C-lobe are colored in cyan and green, respectively. The essential residues are shown as spheres with buried residues colored in red. The exposed essential residues are shown as sticks and labeled with residue numbers colored in magenta. The nearby interacting residues are shown as sticks and labeled with residue numbers colored in black. (B) Ribbon representation of the outward-open model of Cps2J in three different orientations with the same color scheme. D140 engages a salt bridge interaction with K279. S349 makes a hydrogen bond interaction with E65. (C) Cells expressing the indicated Cps2J-FLAG and its corresponding derivatives were grown in BHI with ZnCl_2_ and MnCl_2_. Cultures were diluted and spotted on blood agar as described in the legend of Fig. 2. (D) Quantification of the numbers of CpsJ-FLAG variants by immunoblotting. Cells were grown in BHI with ZnCl_2_ and MnCl_2_, harvested by centrifugation, and resuspended in 2× sample buffer. Proteins were separated by SDS-PAGE, transferred to a membrane, and immunoblotted with anti-FLAG antibodies. *P* values were computed from three biological replicates using the Student’s *t* test.

To identify residues required for the Cps2J function, we performed high-throughput mutagenesis and sequencing (Mut-seq) (41). This approach simultaneously assesses the functional consequences of virtually all single nucleotide polymorphisms (SNPs) in *cps2J*. Briefly, we introduced a PCR mutagenized allele of *cps2J* into a strain where the *cps2E* expression was driven by a P_Zn_ promoter (NUS0267 [*rpsL1* Δ*cps2E*//P_Zn_-*cps2E*]). As mentioned above, when *cps2E* was not induced, cells grew normally regardless of the *cps2J* function. Upon Zn^2+^ addition, only the cells harboring a functional copy of *cps2J* could survive (Fig. 1). The frequencies of SNPs recovered under these two conditions, which represent the fitness cost of each mutation, were compared. In total, we evaluated 3,802 SNPs that encompassed all 1,410 nucleotides of *cps2J*. Of these, 60 SNPs had more than a 5-fold decrease in frequency when selected on Zn^2+^ supplemented plates, indicating that they may have a reduced flippase activity (see Data Set S1). To validate the Mut-seq results, we selected five mutations (G43R, S60L, G342E, G342R, and G346E) and introduced them into the *cps2J* depletion strain (NUS0650 [*rpsL1* Δ*cps2J*::P-*sacB*-*kan*-*rpsL^+^*//Δ*bgaA*::P_Zn_-*cps2J*]) to test whether these alleles remained functional. Indeed, all of these variants were unable to complement *cps2J* (Fig. 5). Without the Cps2J structure solved, it is difficult to understand why these changes will lead to a nonfunctional flippase. Nevertheless, based on the structural model, many essential residues identified are buried and may contribute to protein folding. Unlike MurJ (29), in which several solvent-exposed essential residues were identified in the central aqueous cavity, we only found residue S60 in Cps2J at the kink region of TM2 (Fig. 5). This residue is presumably analogous to residue E57 in MurJ, which was shown to be important for the conformational transition from the inward-open to outward-open state (29, 39). In addition, a few solvent-exposed essential residues are detected in the extracellular and intracellular gates, which may stabilize the inward-open and outward-open states, respectively. Mutation of G43 into bulky residues (e.g., G43R) likely causes clashes with the neighboring residues, therefore disrupting the extracellular gate and favoring the outward-open state. In contrast, mutations of G342 and G346 (G342E/G342R and G346E) on the opposite side perhaps disrupt the intracellular gate and favor the inward-open state (Fig. 5). Lastly, mutations of the two glycine residues into charged residues may also exclude substrate binding. Solving the structure of Cps2J in the future will support or disprove these predictions. To examine whether these changes indeed affect protein stability, we introduced the mutations into the *cps2J*-FLAG construct. When expressed at the native locus, *cps2J*-FLAG could complement *cps2J*^+^ (Fig. 5) Immunoblotting revealed a 2-fold and 5-fold reduction in protein levels of the G43R and S60L variants, respectively, whereas no noticeable change was detected in the levels of the G342E, G342R, and G346R variants. Together, our Mut-seq results suggest that MOP superfamily flippases share a transport mechanism that likely involves a conformational change at the kink region of the TM2.

### Transporting unfinished CPS precursors across the cell membrane resulted in modest cell shape defects and an increased sensitivity to bacitracin.

**FIG 6:**
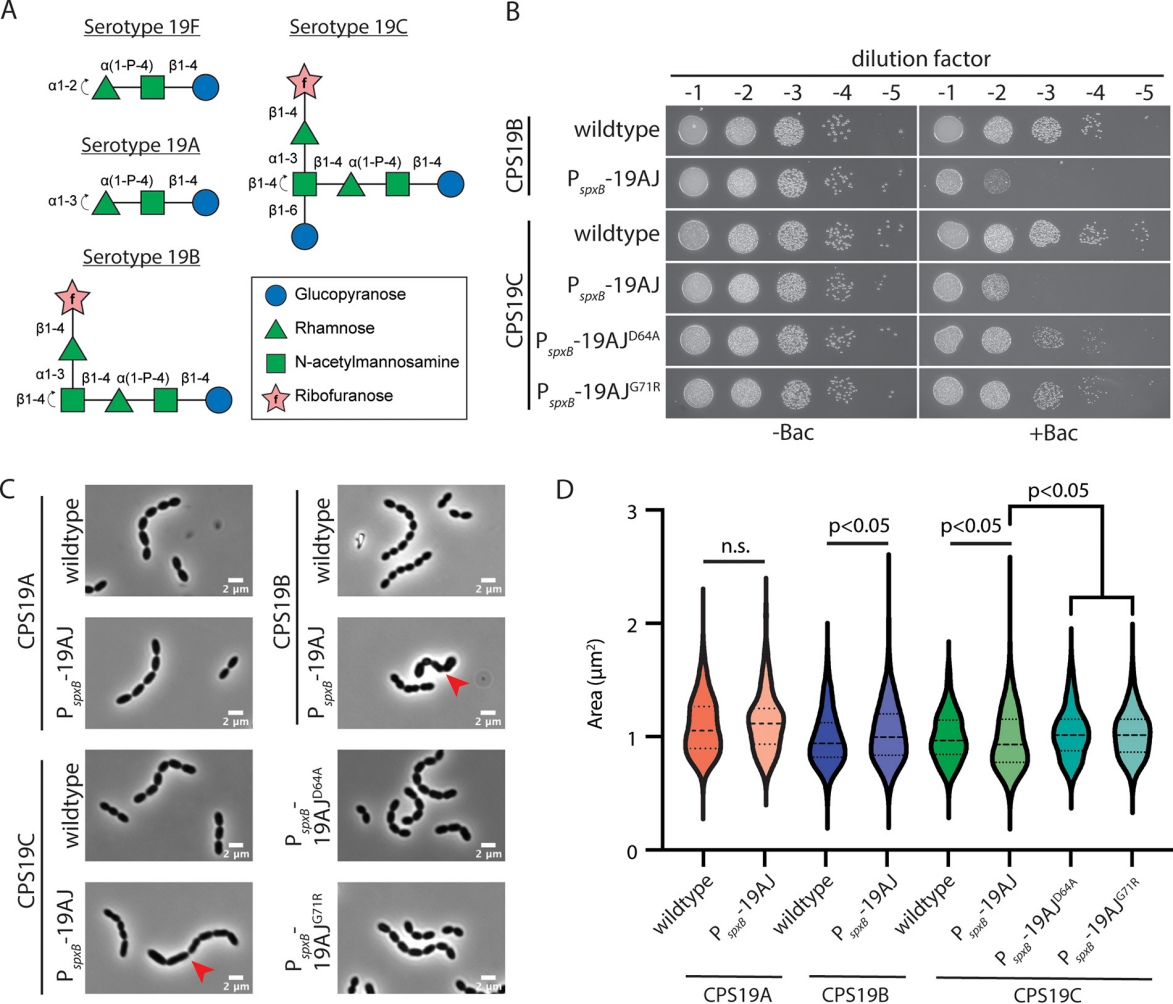
Overexpression of *cps19AJ* in serotypes 19B and 19C resulted in cell shape defects and bacitracin hypersensitivity. (A) Structures of serogroup 19 CPSs drawn in the updated Symbol Nomenclature for Glycans (SNFG) format (12, 66). The reducing ends are on the right, and the sites of polymerization are indicated by the curved arrows on the left. (B) Cells of strains NUS1525 (CPS19B Δ*bgaA*::P-*sacB*-*kan*-*rpsL^+^*), NUS1527 (CPS19B Δ*bgaA*::P*_spxB_*-*cps19AJ*), NUS1568 (CPS19C Δ*bgaA*::P-*sacB*-*kan*-*rpsL^+^*), NUS1584 (CPS19C Δ*bgaA*::P*_spxB_*-*cps19AJ*), NUS2066 [CPS19C Δ*bgaA*::P*_spxB_*-*cps19AJ*(D64A)], and NUS2067 [CPS19C Δ*bgaA*::P*_spxB_*-*cps19AJ*(G71R)] were grown in BHI medium, serially diluted, and spotted on blood plates containing 0.3 μg/ml of bacitracin. Plates were imaged after overnight incubation at 37°C in 5% CO_2_. (C) Cultures of strains NUS0447 (CPS19A Δ*bgaA*::P-*sacB*-*kan*-*rpsL^+^*), NUS1558 (CPS19A Δ*bgaA*::P*_spxB_*-*cps19AJ*), NUS1525, NUS1527, NUS1568, NUS1584, NUS2066, and NUS2067 were grown in BHI to an OD_600_ of 0.2 and then imaged by phase-contrast microscopy. Cells that exhibited cell shape defects are indicated by red arrows. Scale bar, 2 μm. (D) The areas of the cells in panel C were quantified by using MicrobeJ (*n* = 4205). n.s., not significant. *P* values were computed by using the Mann-Whitney U test.

The specificity of MOP transporters could seemingly be relaxed with rather subtle changes in the putative extracellular loops or the central aqueous cavity. This prompted us to investigate the driving force that selects for the substrate selectivity. We hypothesized that the less-specific flippases may accidentally flip incomplete lipid-linked precursors across the cell membrane. Since there is no known mechanism to flip them back into the cytoplasm, the trapped precursors may sequester Und-P and lead to a selective disadvantage. However, mutants expressing WzxC or CpsJ variants with expanded specificity did not exhibit any noticeable growth defect (24). Yet, we have not ruled out the possibility that the variants with growth defects were not recovered with our genetic selection strategy because they were underrepresented. To test whether the expression of a flippase capable of transporting incomplete precursor is detrimental to the cell, we investigated the CPS flippases in serogroup 19. Theoretically, Cps19AJ can compete with the glycosyltransferase WchO in serotypes 19B and 19C by transporting the incomplete precursor ManNAc-Rha-Glc-P-Und to the wrong compartment, before it can be converted to the serotype 19B and 19C repeating units (Fig. 6; see also Fig. S7). First, isogenic capsule-switch mutants that produced serotype 19A, 19B, and 19C capsules were constructed. Next, we cloned *cps19AJ* downstream of a strong constitutive promoter P*_spxB_* and introduced it into an ectopic locus. The P*_spxB_*-*cps19AJ* cassette was functional because it could complement *cps19AJ* in the serotype 19A capsule-switch mutant (NUS1580 [*rpsL1* CPS19A Δ*cps19AJ*::P-*erm* Δ*bgaA*::P*_spxB_*-*cps19AJ*]). We then introduced the P*_spxB_*-*cps19AJ* cassette into the serotype 19B and 19C capsule-switch mutants. Overproduction of *cps19AJ* did not cause any noticeable growth defects in liquid medium, but the strains arguably formed slightly smaller colonies on blood plates (see Fig. S7). The cells also have a mild cell shape defect (Fig. 6). To investigate whether the overproduction of *cps19AJ* caused a reduction in the Und-P level in serotype 19B and 19C strains, we examined whether the cells are hypersensitive to bacitracin (32). Bacitracin binds to undecaprenyl pyrophosphate (Und-PP) and depletes the Und-P pool. If the cell has a lower Und-P level to begin with, the MIC of bacitracin should be reduced. Indeed, strains harboring serotype 19B and 19C capsule with *cps19AJ* overexpressed were more sensitive to bacitracin compared to the parent strains (Fig. 6). Finally, to test whether the transporter function of the *cps19AJ* is required for the bacitracin-sensitive phenotype, inactive variants of *cps19AJ* were constructed and overexpressed in the serotype 19B and 19C capsule-switch mutants. These variants did not lead to the same degree of increase in the bacitracin sensitivity and cell shape defects, albeit the colonies were smaller on plates with bacitracin (Fig. 6). This result argues that the increased bacitracin sensitivity is not due to the general toxicity caused by the overexpression of membrane proteins. Notably, the inactive Cps19AJ variants were produced at a similar level compared to the wild-type Cps19AJ (see Fig. S7), indicating that the mutant proteins are not destabilized. Together, our results suggest that the expression of a flippase that can transport an incomplete precursor may lead to a reduced Und-P pool, which results in an increased sensitivity to bacitracin.

10.1128/mBio.02615-21.8 FIG S7 Overexpression of *cps19AJ* resulted in a mild cell shape defect in serotype 19B and 19C capsule-switch mutants. (A) Cells of strains NUS0282 (CPS19A), NUS1558 (CPS19A P*_spxB_*-*cps19AJ*), NUS0317 (CPS19B), NUS1527 (CPS19B P*_spxB_*-*cps19AJ*), NUS0316 (CPS19C), and NUS1584 (CPS19C P*_spxB_*-*cps19AJ*) were grown in BHI medium. Growth was monitored by measuring the OD_600_ during incubation at 37°C in CO_2_. (B) Cells of NUS0447 (CPS19A Δ*bgaA*::P-*sacB-kan-rpsL^+^*), NUS1558, NUS1580 (CPS19A Δ*cps19AJ* Δ*bgaA*::P*_spxB_*-*cps19AJ*), NUS1525 (CPS19B Δ*bgaA*::P-*sacB*-*kan*-*rpsL^+^*), NUS1527 (CPS19B Δ*bgaA*::P*_spxB_*-*cps19AJ*), NUS1568 (CPS19C Δ*bgaA*::P-*sacB*-*kan*-*rpsL^+^*), NUS1584 (CPS19C Δ*bgaA*::P*_spxB_*-*cps19AJ*), NUS2066 [CPS19C Δ*bgaA*::P*_spxB_*-*cps19AJ*(D64A)], and NUS2067 [CPS19C Δ*bgaA*::P*_spxB_*-*cps19AJ*(G71R)] were grown in BHI, serially diluted, and spotted on blood agar plates. The plates were imaged after overnight incubation at 37°C in 5% CO_2_. (C) Cultures of NUS1525, NUS1527, NUS1568, and NUS1584 were normalized by their optical densities. Cells were harvested by centrifugation and lysed. (D) The CPS recovered was detected by immunoblotting with anti-serotype 19 antibodies and quantified. *P* values were computed using the Student *t* test. n.s., not significant. (E) Detection of Cps19AJ-FLAG and its variants by immunoblotting with anti-FLAG antibodies. The black arrow indicates the expect size of Cps19J-FLAG. (F) Quantification of Cps19AJ and its variants in the immunoblot shown in panel E. Plotted are the averages and standard deviations of three biological replicates. *P* values were calculated using the Student *t* test. n.s., not significant. (G) Serotype 19C CPS synthesis pathway. Overproduction of Cps19AJ may compete with the glycosyltransferase WchO for the trisaccharide lipid-linked precursor. The incomplete precursor at the outer leaflet of the cell membrane may also inhibit the activity of the Cps19CH polymerase. Download FIG S7, PDF file, 0.2 MB. Copyright © 2021 Chua et al. 2021 Chua et al. https://creativecommons.org/licenses/by/4.0/ This content is distributed under the terms of the Creative Commons Attribution 4.0 International license.

## DISCUSSION

The polymorphic CPS is one of the most important virulence factors in S. pneumoniae. Other than serotypes 3 and 37, all pneumococcal CPSs are synthesized by the Wzx/Wzy pathway (3, 4, 12). A critical step in this pathway is the translocation of the lipid-linked precursors across the cytoplasmic membrane. This step is thought to be mediated by the widely conserved MOP-family flippase CpsJ (22). Many studies to elucidate the transport mechanism of MOP transporters were carried out using the lipid II flippase MurJ as a prototype (29, 39, 40, 42, 43). The prevailing model is that the two pseudosymmetrical bundles of transmembrane helices adopt a V-shaped configuration. To initiate the transport cycle, the lipid-linked precursor at the inner leaflet of the cell membrane interacts with the central hydrophilic cavity of the flippase and destabilizes the inward-open conformation, resulting in the inward-occluded fold. This conformation prevents the substrate from diffusing back into the cytoplasm (39), before the transporter transitions to the outward-facing conformation and releases the cargo to the outer leaflet of the cell membrane. To restore the inward-open fold, an energy source such as a Na^+^ or H^+^ gradient is presumably required (25, 42), although this is still a subject of debate (39). Finally, the inward-open MOP flippase initiates another round of substrate translocation.

Photo-cross-linking and biotin-tagging experiments provided evidence that the substrate transport is coupled with the transition between the inward-open and outward-open conformations (40), although the electron density of the cargo could not be unambiguously assigned in the crystal structure (39). Nevertheless, how the interaction between the precursor and the flippase results in the conformational change is still unclear. Biochemically reconstitution of the translocation event is expected to be challenging, because it does not chemically modify the substrate (20). In addition, the lipid-linked cargos are usually difficult to obtain. We reasoned these problems could be partially circumvented by taking a genetic approach. In the present study, CpsJ variants from various serotypes were introduced into a serotype 2 strain, and we tested whether they could replace Cps2J. The nine cross-complementing CpsJ variants clustered around serogroup 33 (see Fig. S1), suggesting this group of flippases may have relatively relaxed specificity. We also demonstrated that Cps33BJ does not support the translocation of PG and teichoic acid precursor, so it is not totally nonspecific. Unlike Cps2J, it appears that Cps33BJ could only be substituted by its close homologs. We noticed that the precursors of CpsJ variants that complemented *cps33BJ* function contain galactofuranose at the non-reducing end (12), similar to the serotype 33B CPS precursor. Perhaps the galactofuranose residue is recognized by the flippases. We have yet to fully understand why certain CPS flippases can transport noncognate substrates. Nevertheless, we believe identifying the unexpected cross-complementation events in the CPS pathway may inform future bioinformatics and structural biology efforts to address the longstanding question of MOP-transporter specificity.

We showed that mutations at the extracellular loops of Cps2J could relax its substrate specificity. Similar mutations were identified in an unrelated MOP flippase WzxC (24), and these results support a unifying transport mechanism. While computational docking experiments using the structural models of CpsJ placed the repeating units in the central aqueous cavity (see Fig. S6), we could not clearly identify a region responsible for substrate binding. Work is under way to reveal the substrate binding pocket by solving the CpsJ structures. In addition, Mut-seq results for Cps2J and MurJ (29) did not detect any strictly immutable residue, indicating that the overall fold of these transporters is robust, although they belong to the evolutionarily diverged MVF (mouse virulence factor) or PST (polysaccharide transport) families.

In response to the host adaptive immunity, the capsule of a bacterial pathogen may need to be constantly modified to escape the anti-CPS antibodies. In addition to the variations of glycosyltransferases that alter the repeating units, the new precursors must be able to reach the other side of the cell membrane to complete synthesis. This can be achieved by the overexpression of the flippase involved (33) or the accumulation of mutations at the extracellular/periplasmic gate (24). In this study, we found that changes in the central aqueous cavity can also allow the transporter to flip a noncognate substrate. Since S. pneumoniae is naturally competent, it may acquire flippase genes from the nasopharyngeal microbiome (44, 45). We propose the flippases in the clade of serogroup 33 (see Fig. S1) may be evolutionary intermediates that retained some promiscuity until another set of mutations convert them back into the presumably more efficient flippases that dedicate to their substrates (46).

One possible driving force that maintains flippase specificity is that a flippase capable of transporting incomplete precursors across the cell membrane may reduce the amount of free Und-P (Fig. 1). This model is supported by the mild toxicity due to the overexpression of a Cps19AJ (Fig. 6). A similar observation was noted when a promiscuous flippase Wzk was overproduced in Escherichia coli (36). This toxicity was alleviated by reducing the inducer concentration (36). In addition to sequestering Und-P, the incomplete precursor on the cell surface may also act as a competitive inhibitor of the downstream enzymes since they are structural homologs of the native substrates. Nevertheless, the phenotype observed in this study was rather mild. A plausible explanation is that CpsJ may be able to translocate the unfinished precursors back to the cytoplasm (see Fig. S7). Alternatively, CPS enzymes may be organized in a supramolecular complex and prevent diffusion of the incomplete precursors through metabolic channeling (47). These possibilities warrant further research. Perhaps more importantly, the incomplete glycoconjugates displayed on the cell surface may be vulnerable to the host’s defense system, such as being recognized by the C-type lectin SIGN-R1 (a murine homolog of DC-SIGN in human) (48). In plants, bacterial flagellin fragments are sensed by the FLS2 receptor, unless they are properly shielded by glycosylation. To “visualize” the flagellin glycopeptides, the host produces glycosidases such as BGAL1 to strip the protective sugar residue at the nonreducing ends (49). As a defense strategy, the MOP flippases in pathogens likely serve as a molecular checkpoint to ensure that the vulnerable glycans remain inside the cytoplasm.

## MATERIALS AND METHODS

### Bioinformatics analysis.

Neighbor-joining phylogenetic trees were generated using the Molecular Evolutionary Genetics Analysis version 7 (MEGA7) software (50). First, CpsJ sequences were retrieved from the National Center for Biotechnology Information (NCBI) website and aligned using the built-in ClustalW program. The evolutionary distance was calculated in the unit of the number of amino acid difference per site using the “p-distance method” and the “15-aa-window” settings of the MEGA7 software. The confidence level of the branch was tested by bootstrapping 500 replicates. Pairwise sequence identities were calculated using Clustal Omega (51). Structural models of CpsJ were generated by I-TASSER (52) and visualized with PyMOL (http://pymol.org). The number of transmembrane helices in the flippases was predicted using TMHMM (53). Structures of the CPS repeating units were downloaded from the bacterial carbohydrate database (http://csdb.glycoscience.ru/), and their PDB files were generated by REStLESS (54). They were docked with the CpsJ structural models using Autodock Vina (55).

### Media, culture conditions, and bacterial strains.

The strains used in this study are listed in Table S1 in the supplemental material. Clinical isolates of S. pneumoniae expressing 93 types of CPS were collected from the local and international hospitals and research institutes, as indicated in Table S1. Unless otherwise specified, cells were grown in brain heart infusion broth (BHI; Thermo Fisher Scientific) or on tryptic soy agar plates supplemented with 5% (vol/vol) sheep blood (blood plates) (Biomed Diagnostics) at 37°C in 5% CO_2_. Antibiotics were purchased from Sigma-Aldrich and used at final concentrations of 0.3 μg/ml for erythromycin (Erm), 250 μg/ml for kanamycin (Kan), 0.3 μg/ml for bacitracin, and 300 μg/ml for streptomycin (Str). We noticed that bacitracin could not be evenly spread on blood plates, and thus we freshly prepared the plates by mixing bacitracin with the agar solution before pouring it. When indicated, ZnCl_2_ and MnCl_2_ were added to liquid cultures at final concentrations of 400 and 40 μM, respectively. Mn^2+^ was added to the medium to alleviate Zn^2+^ toxicity (56, 57). When blood plates were used, the concentrations of ZnCl_2_ and MnCl_2_ were adjusted to 500 and 50 μM, respectively.

### Strain construction.

The primers used for synthesizing the PCR amplicons are listed in Table S1. In general, PCR fragments were synthesized using high-fidelity Phusion DNA polymerase (NEB M0530S) according to the manufacturer’s instructions. PCR products were diagnosed by gel electrophoresis and purified using a QIAquick PCR purification kit (Qiagen, catalog no. 28106). Pneumococcal cells were transformed after inducing natural competence, with cassettes assembled by overlap extension PCR or isothermal assembly (58, 59). Transformants were selected on blood plates supplemented with the indicated antibiotics. Allelic replacement was performed using the Janus cassette (P-*kan*-*rpsL^+^*) or the Sweet Janus cassette (P-*sacB*-*kan*-*rpsL^+^*) as described previously (38, 60). We could not obtain amplicons of *cpsJ* from serogroup 11, serotype 16A, and serotype 35F, but the remaining 82 *cpsJ* variants were introduced into strain NUS0650 (*rpsL1* Δ*cps2J*::P-*sacB*-*kan*-*rpsL*^+^//Δ*bgaA*::P_Zn_-*cps2J*) by transforming the cassette harboring the corresponding *cpsJ* allele. Transformants were selected for Str and sucrose resistance. The resulting strains were validated by PCR using GoTaq DNA polymerase (Promega, M712) and Sanger sequencing.

### Microscopy and measurements of growth.

Overnight cultures of strain NUS0063 (Δ*bgaA*::P_Zn_-*cps2J*) and NUS0084 (Δ*cps2J*//Δ*bgaA*::P_Zn_-*cps2J*) were grown in BHI supplemented with ZnCl_2_ and MnCl_2_, as described above. When the optical density at 600 nm (OD_600_) reached 0.2 to 0.4, cultures were normalized to an OD_600_ of 0.2. Cells were pelleted by centrifugation, washed with 1 ml of BHI medium twice to remove residual ZnCl_2_ and MnCl_2_, and diluted to an OD_600_ of 0.01 in BHI with or without ZnCl_2_/MnCl_2_. The diluted cultures were distributed into a 96-well plate at a final volume of 200 μl. Growth was monitored at 37°C for 10 h using a Tecan microplate reader. OD_600_ readings were taken every 10 min immediately after a short purse with 30 s of shaking.

To visualize the cells deprived of *cps2J*, overnight culture of strain NUS0084 (Δ*cps2J*//Δ*bgaA*::P_Zn_-*cps2J*) was diluted to an OD_600_ of 0.01 and grew for ∼6 h at 37°C. Just prior to the onset of cell lysis, the cells were harvested by centrifugation at 3,000 × *g* for 5 min at room temperature. The supernatant was removed, and the pellets were resuspended in 50 μl of BHI. Cells were mounted on a glass slide and imaged with an IX81 phase-contrast microscope (Olympus). Micrographs were analyzed and quantitated using MicrobeJ, as described previously (61).

### Immunoblotting.

Immunoblotting was done essentially as described previously (62). Briefly, cultures were grown to an OD_600_ of 0.2 to 0.4. After adjusting the OD_600_ of the culture to 0.3, 1 ml of the culture was collected and centrifuged at 16,100 × *g* for 1 min at room temperature. Spent medium was discarded and the pellets were washed once with 1× phosphate-buffer saline (PBS) and resuspended in 200 μl of protoplast buffer (0.5 M sucrose, 20 mM MgCl_2_, 20 mM Tris-HCl [pH 7]). To normalize the samples based on protein concentrations, we measured the amount of protein in the cell suspension using the NI protein assay kit (G-Biosciences, catalog no. 786-005). Alternatively, normalization of the loading volume based on the OD_600_ readings of the culture produced nearly identical results. The suspension was treated by adding 4 μl of 10 U/μl mutanolysin (Sigma-Aldrich, catalog no. M9901-10KU) and 15 μl of 10 mg/ml lysozyme (Sigma-Aldrich, 62970-1G-F) to digest the cell wall. Spheroplast formation was confirmed by microscopy, and they were pelleted by centrifugation at 3,000 × *g* for 10 min at room temperature. Supernatant contain the digested cell wall were collected. The pellets (spheroplasts) were lysed by resuspension in 200 μl of 1× PBS. Samples were mixed with an equal volume of 2× Laemmli sample buffer (Bio-Rad), digested by adding 2 μl of 20 mg/ml proteinase K (Qiagen 19133) and incubation at 50°C for 1 h, before separated to a 4/10% SDS-PAGE gel. The CPS was transferred to a polyvinylidene difluoride membrane and blocked by incubation for 30 min in blocking solution (PBST; 1× PBS with 0.05% [vol/vol] Tween 20) with 5% (wt/vol) skim milk. To the blot, 50 μl of cell lysate from strain HMS0002 (*rpsL1* Δ*cpsE*) was added to minimize antibody binding to non-CPS materials (22). After mixing for 2 min, the anti-CPS serum was added at a dilution of 1:5,000, and the blot was incubated overnight at 4°C with shaking. The blot was washed two times with PBST for 5 min and incubated with the anti-rabbit antibodies conjugated with horseradish peroxidase (HRP; Thermo Fisher Scientific, A16110) at a dilution of 1:10,000 in blocking solution. After incubation for 1 h at room temperature with shaking, the membrane was washed three times with PBST and detected by using enhanced chemiluminescence reagents (Thermo Scientific, catalog no. 34580) according to the manufacturer’s protocol.

To detect FLAG-tagged CpsJ (63), strains expressing *cpsJ-FLAG* variants (*cps2J<>cpsJ-*FLAG//P_Zn_-*cps2J*) were grown in BHI with ZnCl_2_/MnCl_2._ Cultures were normalized to an OD_600_ of 0.3 and then centrifuged at 16,100 × *g* for 1 min at room temperature; next, the pellets were resuspended in 100 μl of 1× PBS. The cell suspension was mixed in a 1:1 ratio with 2× Laemmli buffer containing 5% (vol/vol) β-mercaptoethanol and incubated at 50°C for 5 min. Proteins were separated on a 4/12% SDS-PAGE gel, transferred to a polyvinylidene difluoride membrane, and blotted with the anti-FLAG polyclonal antibodies (1:3,000 dilution; Sigma-Aldrich, F7425), followed by anti-rabbit antibodies conjugated to HRP (1:5,000 dilution). Signal detection was done using the chemiluminescence substrate as described above.

### Mutagenesis sequencing and isolation of *cpsJ* variants with an expanded specificity.

Amplicons of *cps2J* and *cps23BJ* were PCR mutagenized as described before (24, 64) except using the oligonucleotides listed in Table S1 and GoTaq DNA polymerase. Briefly, the *cps2J* or *cps23BJ* cassettes were amplified for 28 cycles to ensure a low mutation rate. As a negative control, the same amplicons were synthesized using Phusion DNA polymerase. PCR products were purified and concentrated using the Qiagen PCR purification kit. Strain NUS0893 (*rpsL1* Δ*cps2E* Δ*cps2J*::P-*sacB-kan-rpsL^+^*//*ΔbgaA*::P_Zn_-*cps2E*) was transformed with the mutagenized and control PCR amplicons and selected for Str and sucrose resistance in the absence of ZnCl_2_/MnCl_2_. Transformants were pooled and stored at −80°C or directly used to select for the phenotypes described below.

For Mut-seq, the mutagenized *cps2J* library was plated on blood plates with or without ZnCl_2_/MnCl_2_. Colonies on the plates were resuspended in BHI and pooled. The suspension was adjusted to an OD_600_ of ∼0.7 with BHI, and genomic DNA was extracted using a DNeasy Blood & Tissue kit (Qiagen, catalog no. 69506). The *cps2J* region was amplified by KAPA DNA polymerase (Roche, catalog no. KK2611) using primers P1694 and P1695. The amount of PCR amplicons was quantified using the Qubit dsDNA HS assay kit (Invitrogen), and the *cps2J* allele was sequenced on a NovaSeq 6000 platform (NovogeneAIT). Data analysis was performed using the CLC workbench software (Qiagen) as described previously (29). In brief, sequencing reads were mapped to the *cps2J* region with the “length” and “similarity fraction” parameters set to 1. Unmapped reads were collected and aligned again to the *cps2J* open reading frame, but with the “similarity fraction” reduced to 0.98. Base changes were detected by the variant detection tool in the CLC workbench software and exported to Microsoft Excel for further analysis.

To select for the *cps23BJ* variants that can transport serotype 2 CPS precursor, cells harboring mutagenized *cps23BJ* alleles in the NUS0893 (*rpsL1* Δ*cps2E* Δ*cps2J*::P-*sacB*-*kan*-*rpsL^+^*//P_Zn_-*cps2E*) background were plated on blood plates with ZnCl_2_/MnCl_2_ after serial dilutions. Under this condition, cells harboring *cps23BJ* variants that were unable to flip the serotype 2 CPS precursor could not survive. Indeed, we observed a significant increase (∼10-fold) in plating efficiency for cells expressing a mutagenized copy of *cps23BJ*, compared to the cells expressing *cps23BJ*^+^. Survivors were screened for capsule production by immunostaining using the antisera specific for serotype 2 CPS (SSI; i.e., Quellung reaction) (65). Encapsulated mutants were stored, and the *cps23BJ* alleles were sequenced. To ensure the phenotype is linked to the *cps23BJ* alleles, the variants were PCR-amplified and used to transform the parent strain NUS0893 (*rpsL1* Δ*cps2E* Δ*cps2J*::P-*sacB-kan-rpsL^+^*//*ΔbgaA*::P_Zn_-*cps2E*). Similarly, the gain-of-function *cps23BJ* alleles were introduced into strain NUS0650 (*rpsL1* Δ*cps2J*::P-*sacB*-*kan*-*rpsL*^+^//Δ*bgaA*::P_Zn_-*cps2J*) in which the CPS level is similar to that of the wild-type strain.
