# Supplementary figures and images for: High-Throughput Mutagenesis and Cross-Complementation Experiments Reveal Substrate Preference and Critical Residues of the Capsule Transporters in Streptococcus pneumoniae
Source: mBio. 2021 Nov 2;12(6):e02615-21. doi: 10.1128/mBio.02615-21 (PMC8561386; doi:10.1128/mBio.02615-21)

A

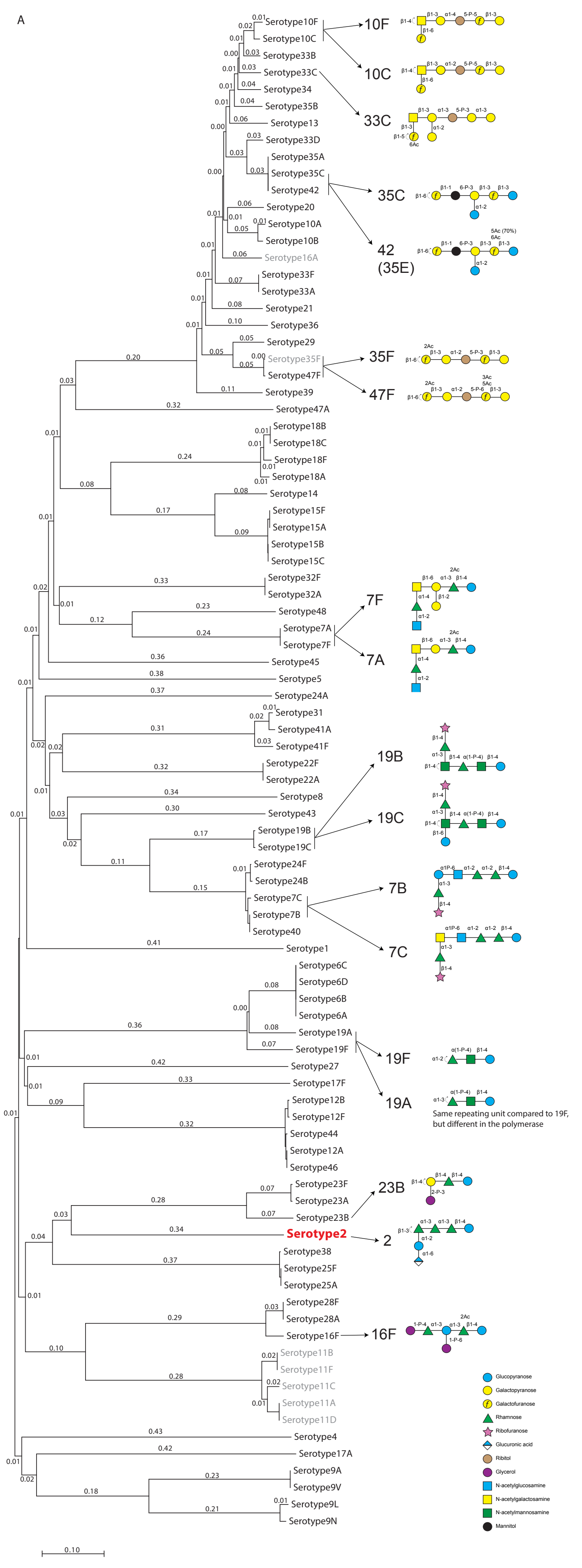

B

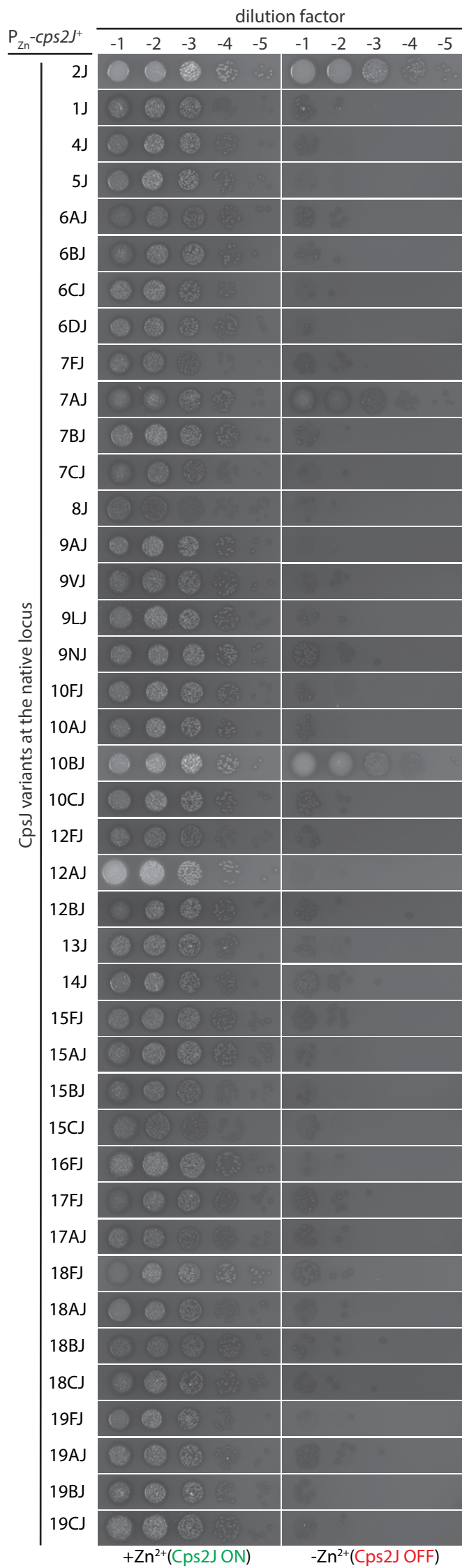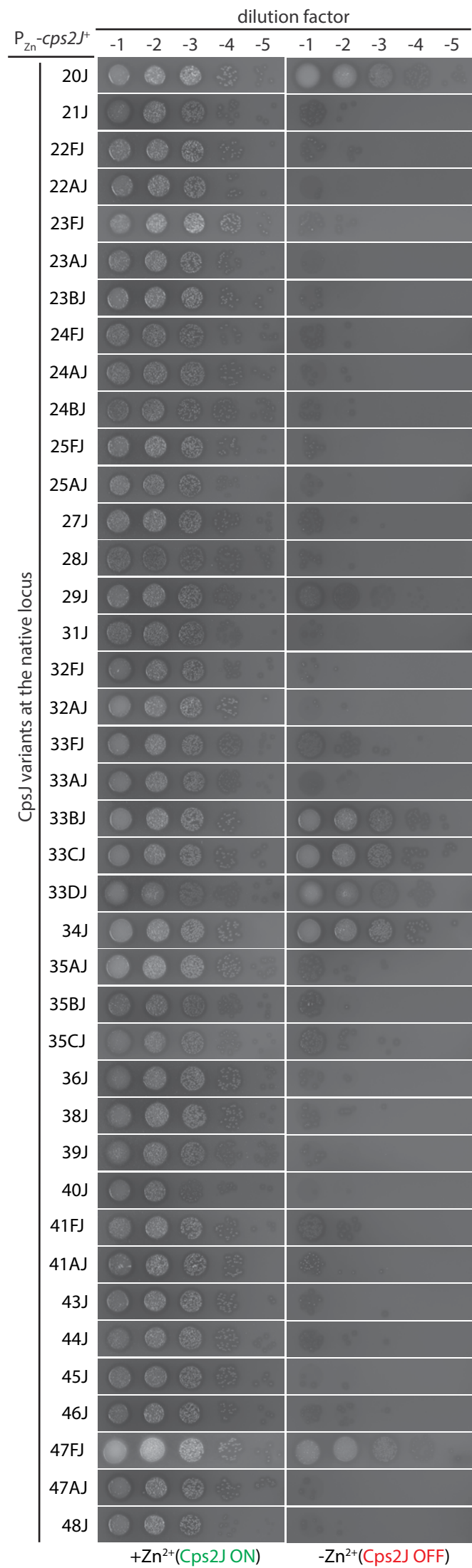

Supplement: FIG S1 [file mbio.02615-21-sf001.pdf]

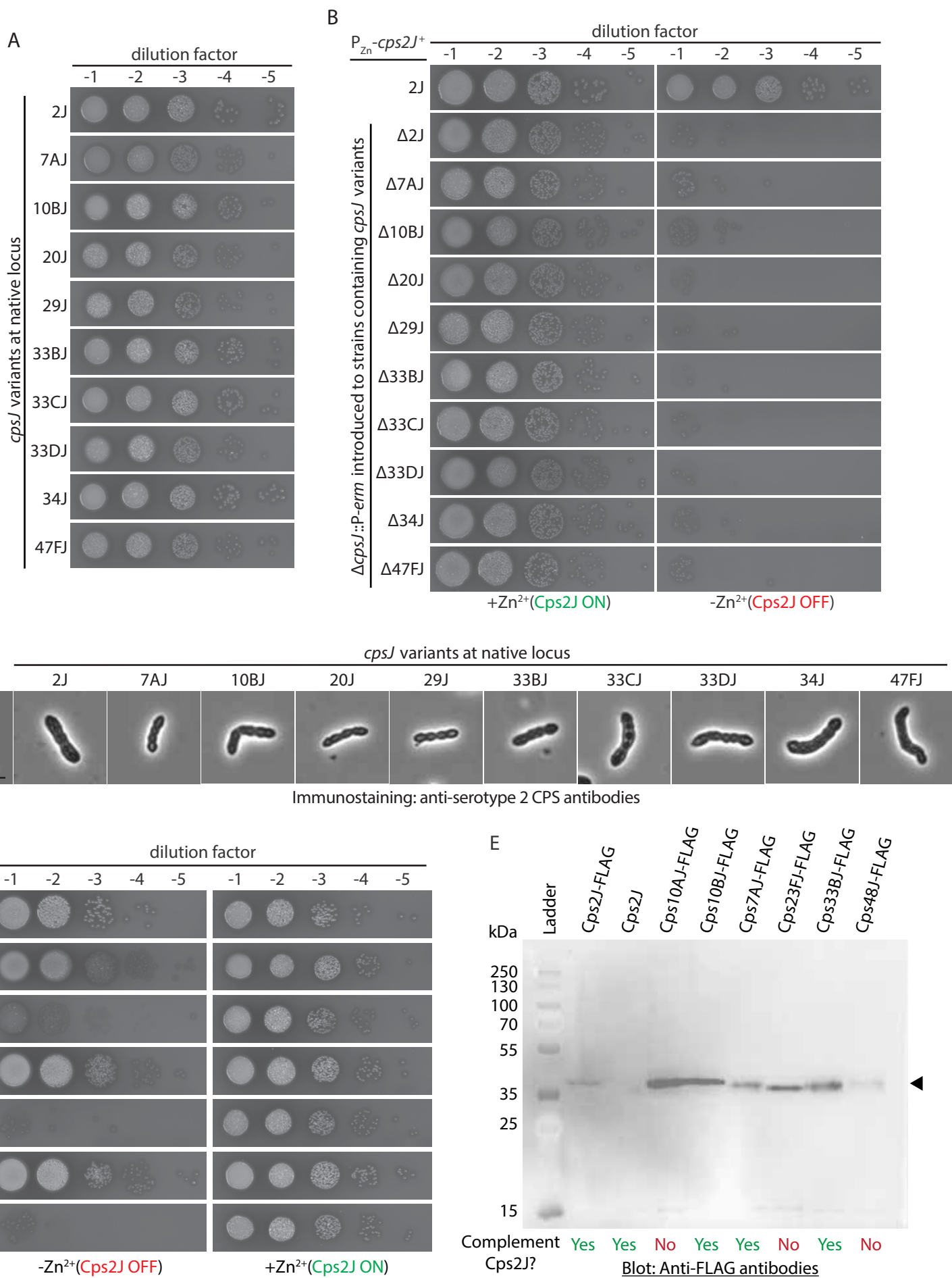

Supplement: FIG S2 [file mbio.02615-21-sf002.pdf]

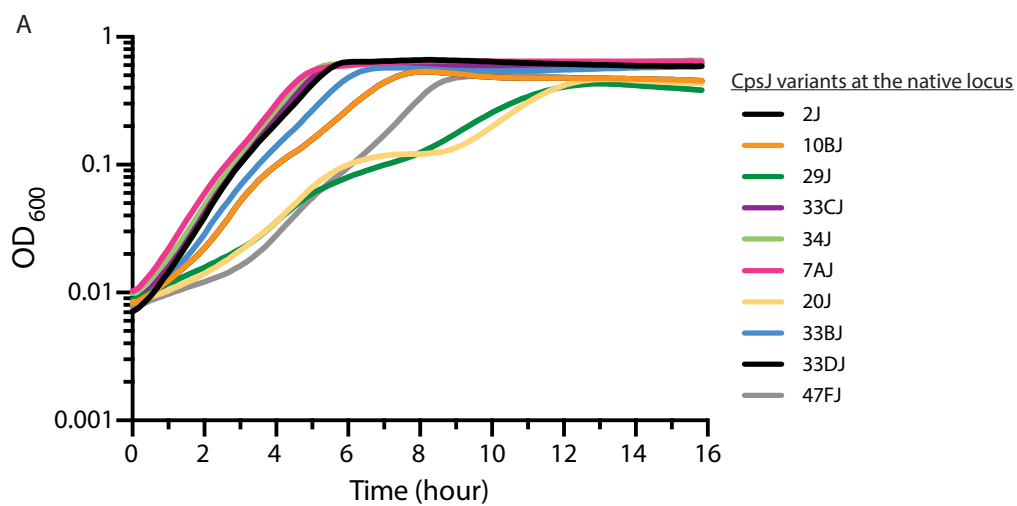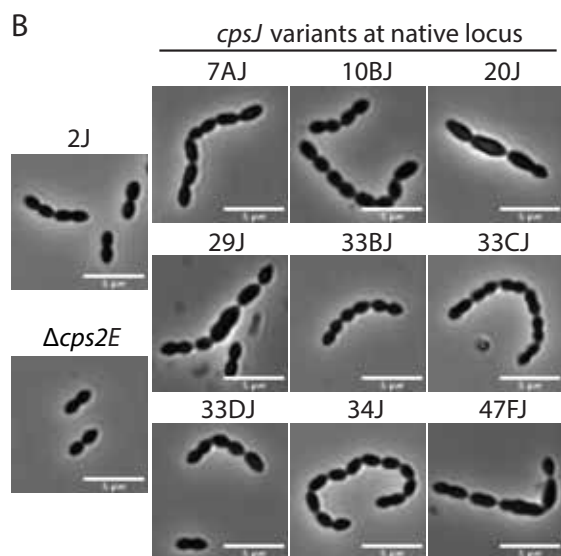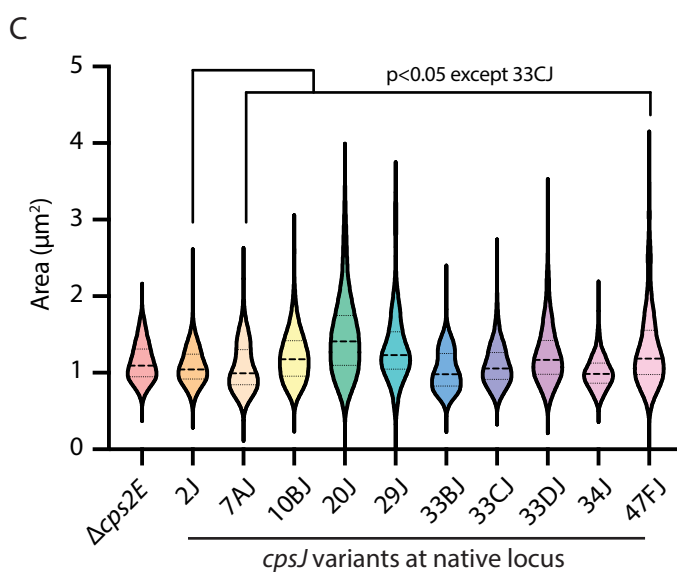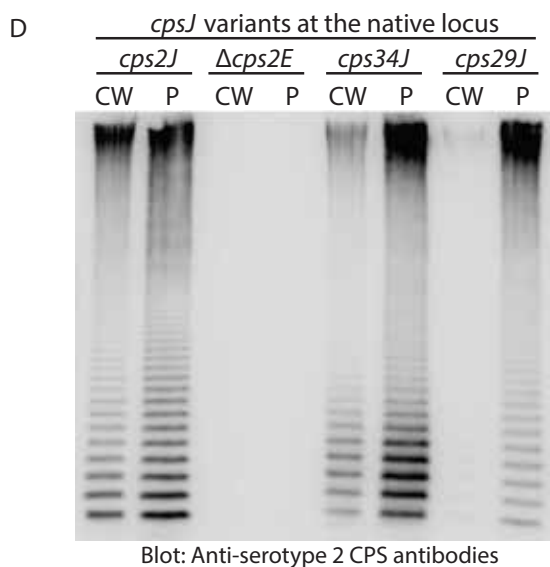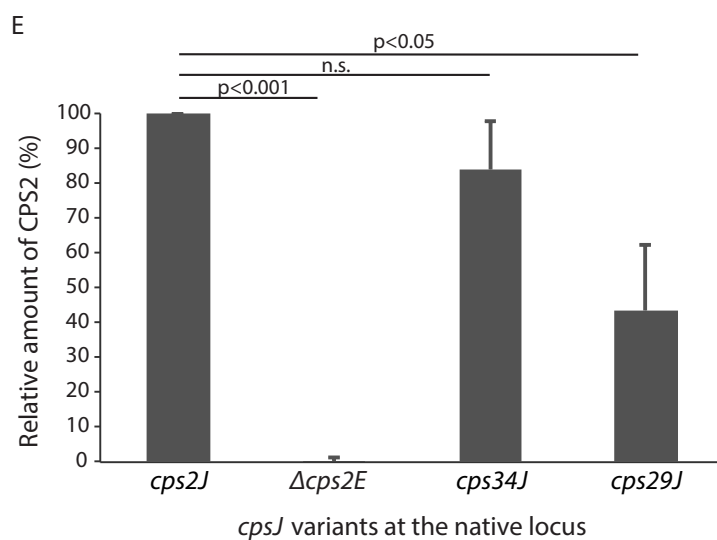

Supplement: FIG S3 [file mbio.02615-21-sf003.pdf]

A

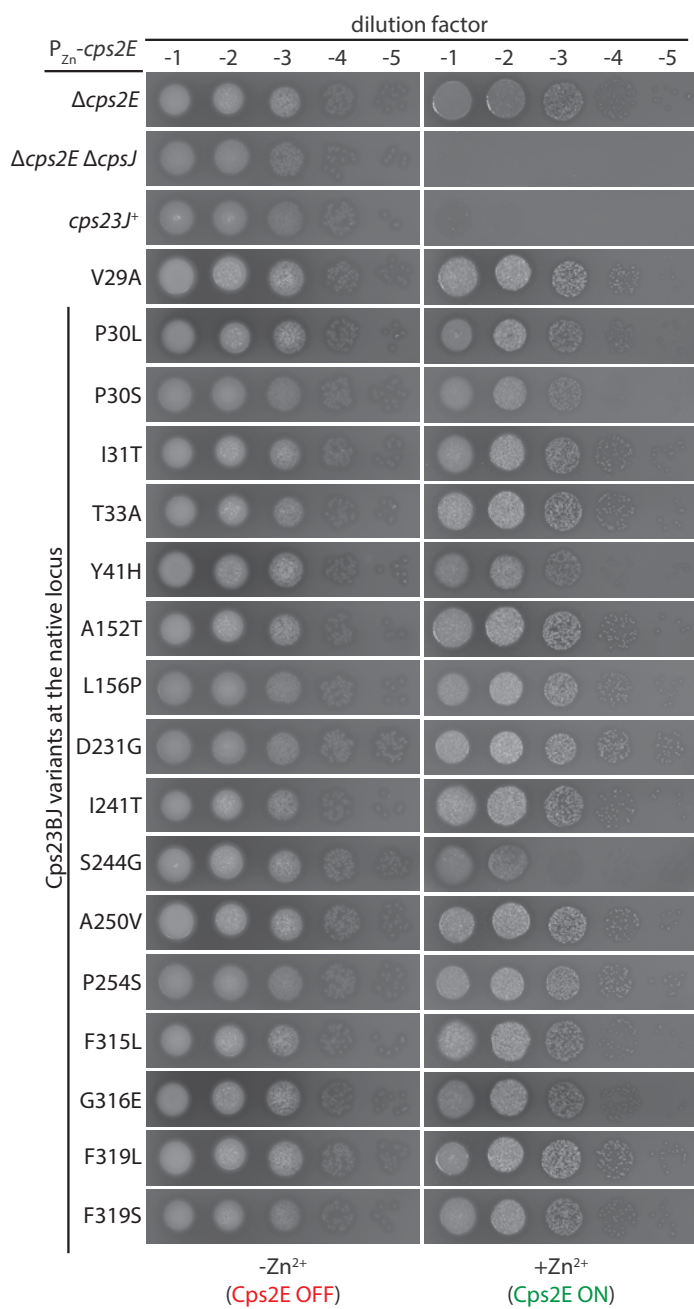

B

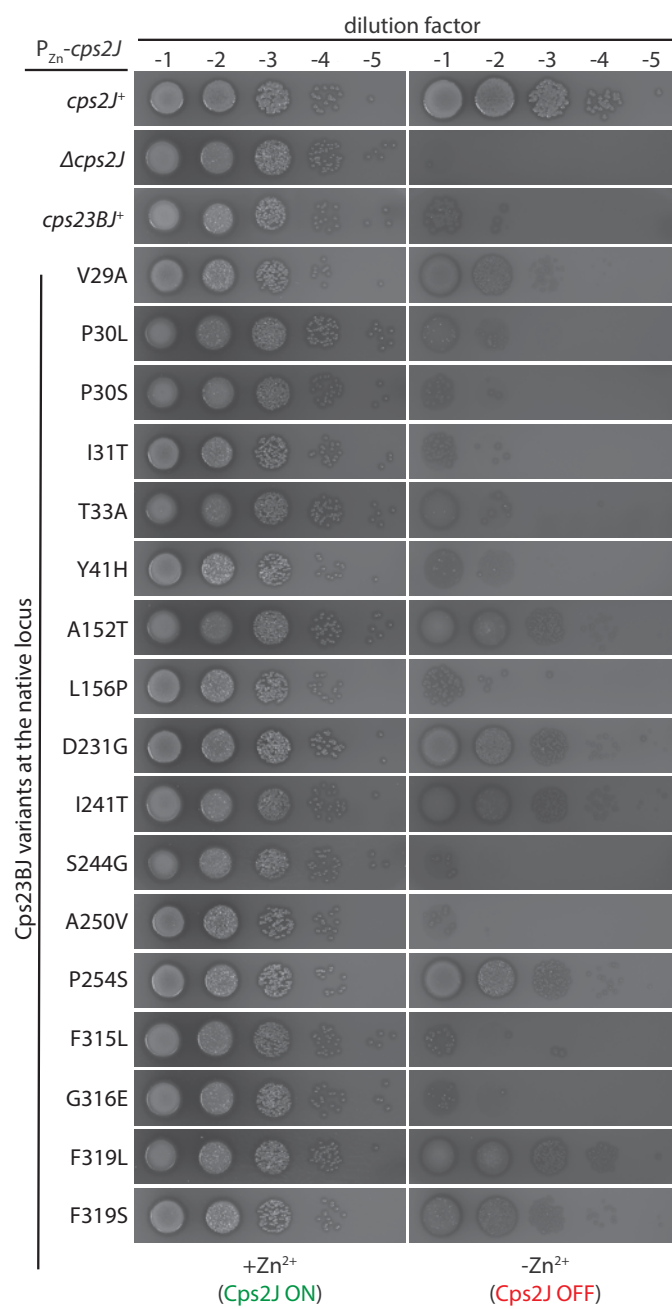

C

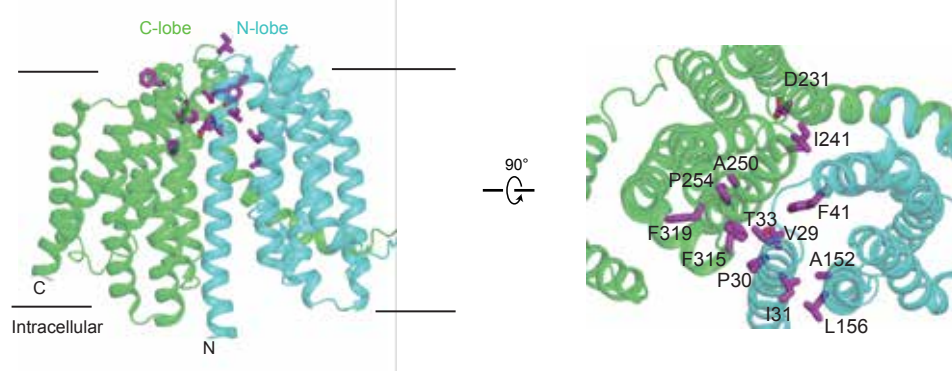

Supplement: FIG S5 [file mbio.02615-21-sf005.pdf]

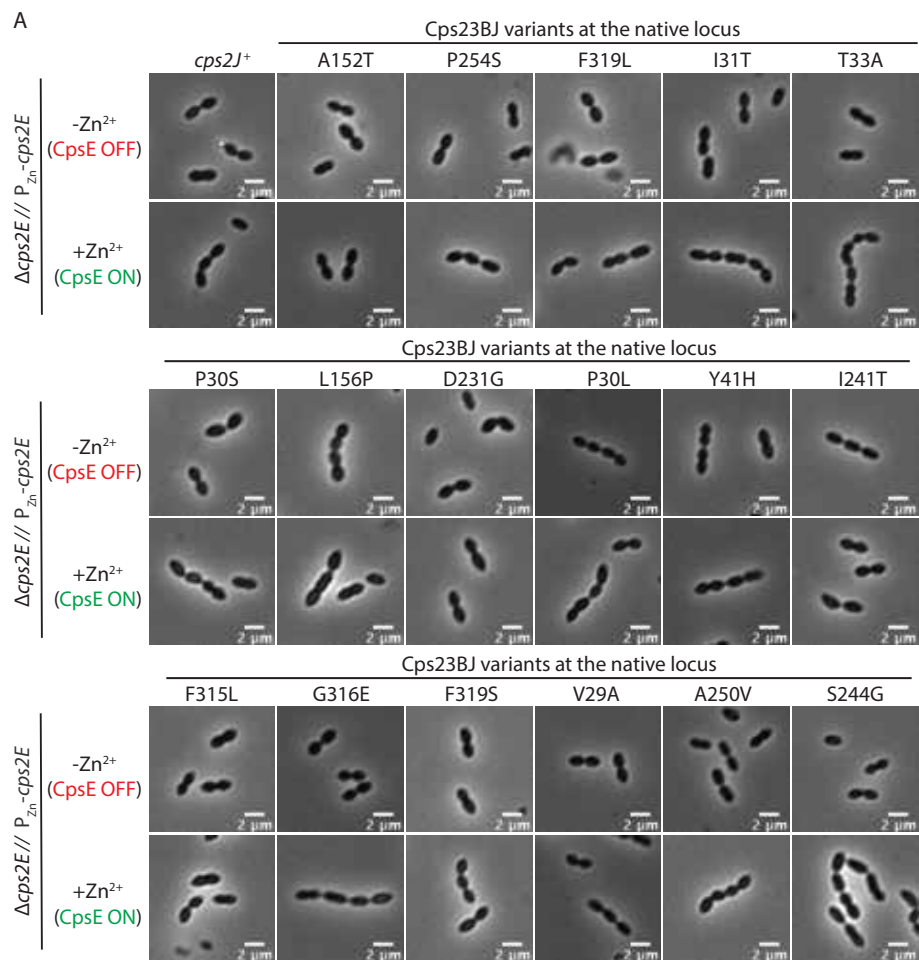

**B**

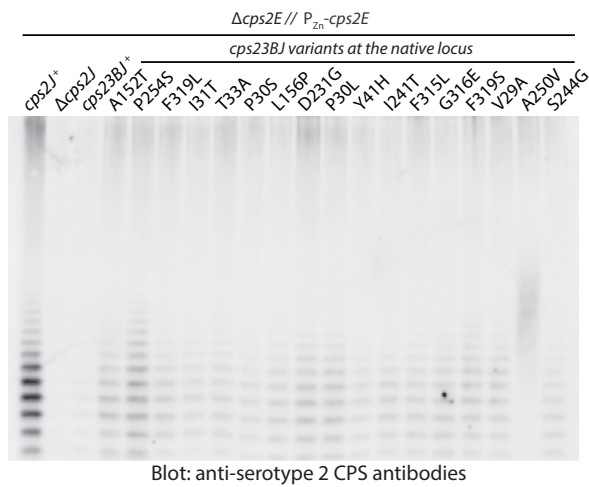

**C**

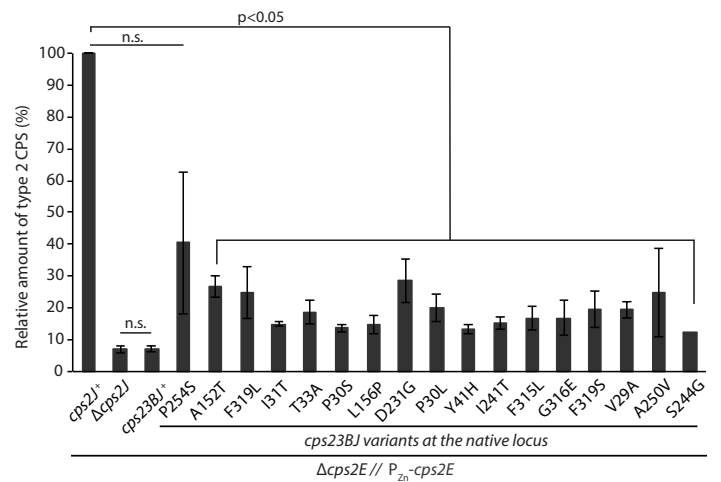

**D**

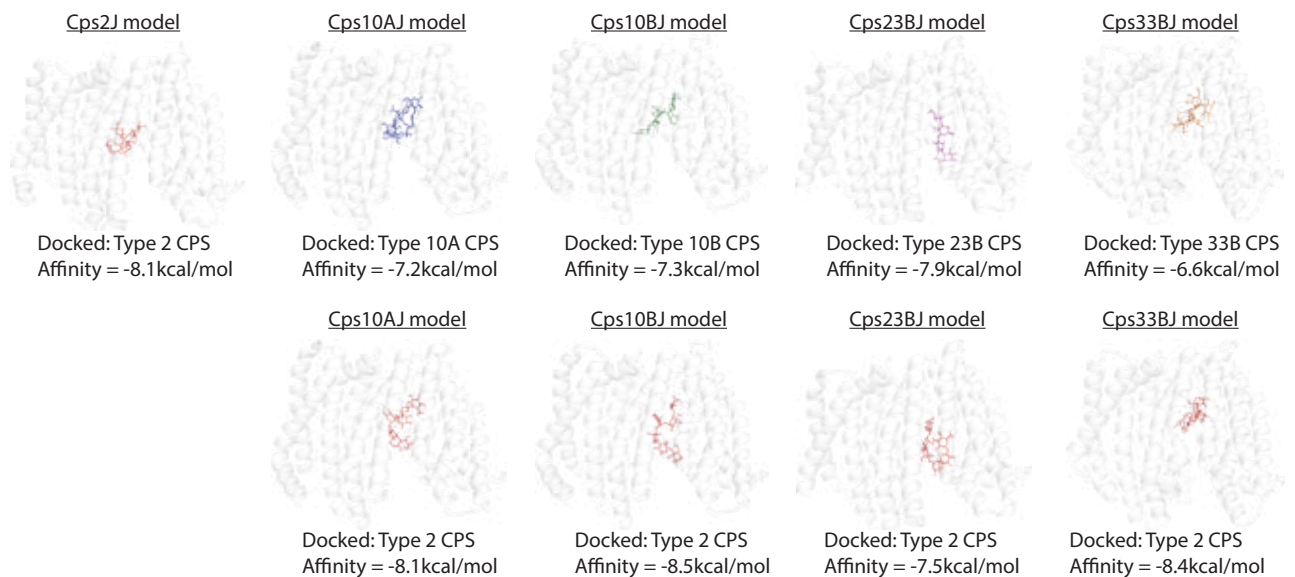

Supplement: FIG S6 [file mbio.02615-21-sf006.pdf]

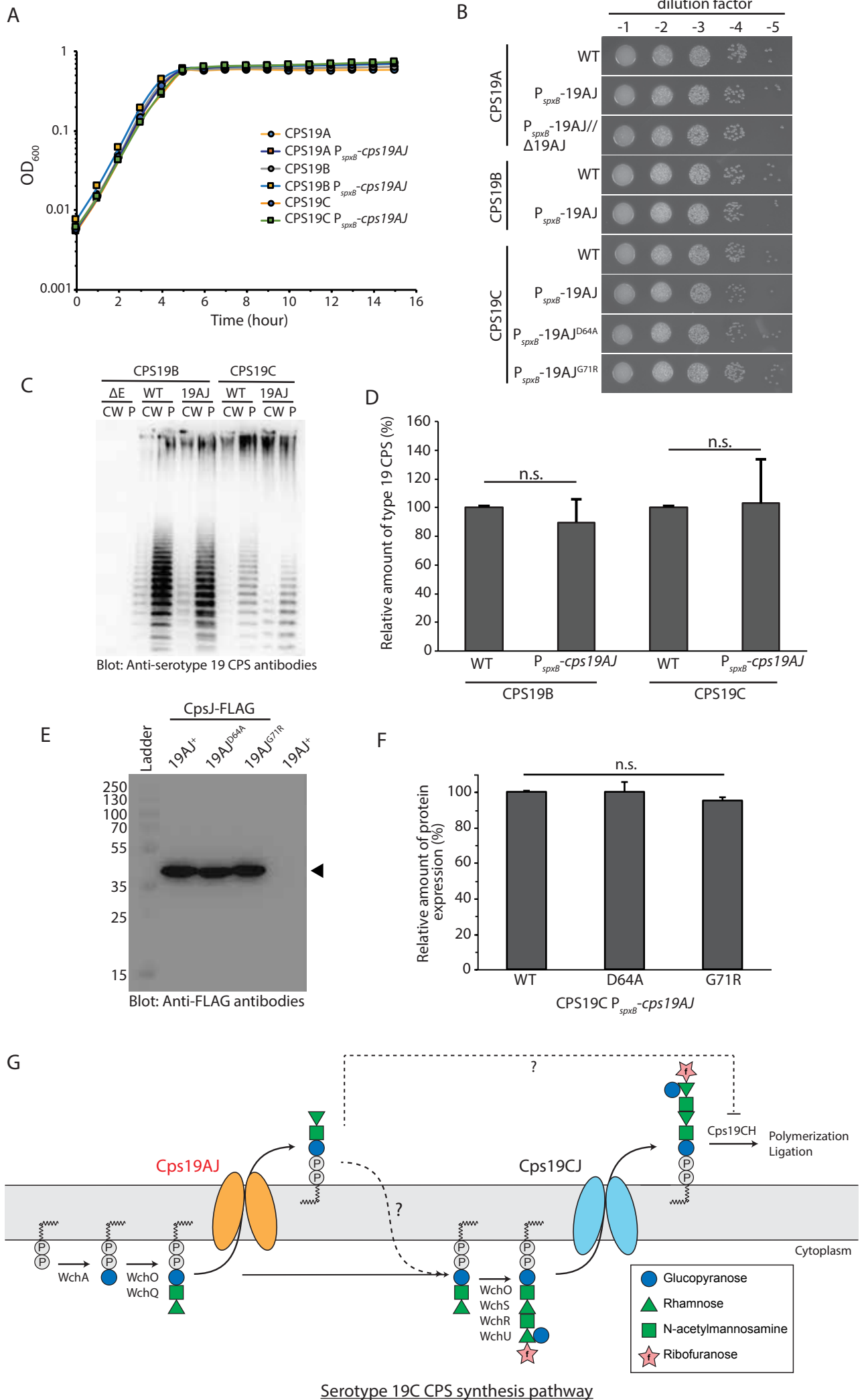

Supplement: FIG S7 [file mbio.02615-21-sf007.pdf]
