# Supplementary material for: High-Throughput Mutagenesis and Cross-Complementation Experiments Reveal Substrate Preference and Critical Residues of the Capsule Transporters in Streptococcus pneumoniae
Source: mBio. 2021 Nov 2;12(6):e02615-21. doi: 10.1128/mBio.02615-21 (PMC8561386; doi:10.1128/mBio.02615-21)
Supplement: TABLE S1 [file mbio.02615-21-st001.docx]

**Table S1A.** Bacterial strains used in this study

| Strain | Relevant genotype^[[1]](#endnote-1),b,c^ | Derivation | Selectable marker^d^ | Source |
| --- | --- | --- | --- | --- |
| IU1690 | serotype 2 strain D39 | - | None | (3) |
| IU1781 | *rpsL1* | *rpsL1* x IU1690 | Str^R^ | (1) |
| AKF_Spn024 | D39 ∆*bgaA*::*tet*-P_Zn_::*pbp1a* | - | Tet^R^ | (2) |
| SpnYL001 | TIGR4S ∆*cps*::*sacB*-*kan*-*rpsL*^+^ | - | Kan^R^, Str^S^, Suc^S^ | (4) |
| HMS0001 | *rpsL1* ∆*cps2E*<>P-*kan-rpsL^+^* | ∆*cps2E*<>P-*kan-rpsL^+^* $\times$ IU1781 | Kan^R^, Str^S^ | This study |
| HMS0002 | *rpsL1* ∆*cps2E* | ∆*cps2E* $\times$ HMS0001 | Str^R^ | This study |
| HMS0007 | *rpsL1* ∆*cps2E* ∆*cps2J*::P-*erm* | ∆*cps2J*::P-*erm* $\times$ HMS0002 | Erm^R^ | This study |
| HMS0019 | *rpsL1* ∆*CEP*::P-*kan*-*rpsL* | ∆*CEP*::P-*kan*-*rpsL* x IU1781 | Kan^R^, Str^S^ | This study |
| NUS0013 | *rpsL1* ∆*bgaA*::P-*erm* | ∆*bgaA*::P-*erm* $\times$ IU1781 | Erm^R^ | This study |
| NUS0015 | *rpsL1* ∆*cps2E* ∆*bgaA*::P-*erm* | ∆*bgaA*::P-*erm* $\times$ HMS0002 | Erm^R^ | This study |
| NUS0016 | *rpsL1* ∆*bgaA*::P-*kan-rpsL^+^* | ∆*bgaA*::P-*kan-rpsL^+^* $\times$ IU1781 | Kan^R^, Str^S^ | This study |
| NUS0063 | *rpsL1* ∆*bgaA*::P_Zn_-*cps2J* | ∆*bgaA*::P_Zn_-*cps2J* $\times$ NUS0016 | Str^R^ | This study |
| NUS0064 | *rpsL1* ∆*cps2J*::P-*kan*-*rpsL^+^* ∆*bgaA*::P_Zn_-*cps2J* | ∆*cps2J*::P-*kan-rpsL^+^* $\times$ NUS0063 | Kan^R^, Str^S^, Zn^2+^ | This study |
| NUS0084 | *rpsL1* ∆*cps2J* ∆*bgaA*::P_Zn_-*cps2J* | ∆*cps2J* $\times$ NUS0064 | Str^R^, Zn^2+^ | This study |
| NUS0650 | *rpsL1* ∆*cps2J*::P-*sacB*-*kan*-*rpsL^+^* ∆*bgaA*::P_Zn_-*cps2J* | ∆*cps2J*::P-*sacB*-*kan*-*rpsL^+^*$\times$ NUS0063 | Kan^R^, Str^S^, Suc^S^ | This study |
| NUS0122 | *rpsL1* ∆*cps2E* ∆*bgaA*::P-*sacB*-*kan*-*rpsL^+^* | ∆*bgaA*::P-*sacB*-*kan*-*rpsL^+^* $\times$ HMS0002 | Kan^R^, Str^S^, Suc^S^ | This study |
| NUS0267 | *rpsL1* ∆*cps2E* ∆*bgaA*::P_Zn_-*cps2E* | ∆*bgaA*::P_Zn_-*cps2E* $\times$ NUS0122 | Str^R^ | This study |
| NUS0454 | *rpsL1* ∆*cps2E* ∆*cps2J*::P-*kan-rpsL^+^* ∆*bgaA*::P_Zn_-*cps2E* | ∆*cps2J*::P-*kan-rpsL^+^* $\times$ NUS0267 | Kan^R^, Str^S^ | This study |
| NUS0893 | *rpsL1* ∆*cps2E* ∆*cps2J*::P-*sacB*-*kan*-*rpsL^+^* ∆*bgaA*::P_Zn_-*cps2E* | ∆*cps2J*::P-*sacB*-*kan*-*rpsL^+^* $\times$ NUS0267 | Kan^R^, Str^S^, Suc^S^ | This study |
| NUS0114 | *rpsL1* ∆*cps*::P-*sacB*-*kan-rpsL^+^* | ∆*cps*::P-*sacB*-*kan*-*rpsL^+^* $\times$ IU1781 | Kan^R^, Str^S^, Suc^S^ | This study |
| NUS0308 | *rpsL1* CPS33B | CPS33B $\times$ NUS0114 | Str^R^, | This study |
| NUS0451 | *rpsL1* CPS33B ∆*bgaA*::P-*kan-rpsL^+^* | ∆*bgaA*::P-*kan-rpsL^+^* $\times$ NUS0308 | Kan^R^, Str^S^ | This study |
| NUS0458 | *rpsL1* CPS33B ∆*bgaA*::P_Zn_-*cps33BJ* | ∆*bgaA*::P_Zn_-*cps33BJ* $\times$ NUS0451 | Str^R^, Zn^2+^ | This study |
| NUS0490 | *rpsL1* CPS33B ∆*cps33BJ*::P-*kan*-*rpsL^+^* ∆*bgaA*::P_Zn_-*cps33BJ* | ∆*cps33BJ*::P-*kan*-*rpsL^+^* $\times$ NUS0458 | Kan^R^, Str^S^, Zn^2+^ | This study |
| NUS1549 | *rpsL1* CPS33B ∆*cps33BJ*::P-*sacB-kan-rpsL* ∆*bgaA*::P_Zn_-*cps33BJ* | ∆*cps33BJ*::P-*sacB-kan*-*rpsL^+^* $\times$ NUS0458 | Kan^R^, Str^S^, Suc^S^, Zn^2+^ | This study |
| NUS0725 | *rpsL1* CPS33B ∆*cps33BJ*::P-*erm* ∆*bgaA*::P_Zn_-*cps33BJ* | ∆*cps33BJ*::P-*erm* $\times$ NUS0552 | Erm^R^, Zn^2+^ | This study |
| NUS0270 | *rpsL1* ∆*cps34J*::P*-erm* ∆*bgaA*::P_Zn_-*cps2J* | ∆*cpsJ*::P-*erm* $\times$ NUS0096 | Erm^R^, Str^R^, Zn^2+^ | This study |
| NUS0298 | *rpsL1* ∆*cps33BJ*::P-*erm* ∆*bgaA*::P_Zn_-*cps2J* | ∆*cpsJ*::P-*erm* $\times$ NUS0266 | Erm^R^, Str^R^, Zn^2+^ | This study |
| NUS0299 | *rpsL1* ∆*cps33CJ*::P-*erm* ∆*bgaA*::P_Zn_-*cps2J* | ∆*cpsJ*::P-*erm* $\times$ NUS0142 | Erm^R^, Str^R^, Zn^2+^ | This study |
| NUS0395 | *rpsL1* ∆*cps33DJ*::P-*erm* ∆*bgaA*::P_Zn_-*cps2J* | ∆*cpsJ*::P-*erm* $\times$ NUS0362 | Erm^R^, Str^R^, Zn^2+^ | This study |
| NUS0724 | *rpsL1* ∆*cps10BJ*::P-*erm* ∆*bgaA*::P_Zn_-*cps2J* | ∆*cpsJ*::P-*erm* $\times$ NUS0550 | Erm^R^, Str^R^, Zn^2+^ | This study |
| NUS0961 | *rpsL1* ∆*cps47FJ*::P-*erm* ∆*bgaA*::P_Zn_-*cps2J* | ∆*cpsJ*::P-*erm* $\times$ NUS0658 | Erm^R^, Str^R^, Zn^2+^ | This study |
| NUS1233 | *rpsL1* ∆*cps7AJ*::P-*erm* ∆*bgaA*::P_Zn_-*cps2J* | ∆*cpsJ*::P-*erm* $\times$ NUS0539 | Erm^R^, Str^R^, Zn^2+^ | This study |
| NUS1234 | *rpsL1* ∆*cps20J*::P-*erm* ∆*bgaA*::P_Zn_-*cps2J* | ∆*cpsJ*::P-*erm* $\times$ NUS0835 | Erm^R^, Str^R^, Zn^2+^ | This study |
| NUS1235 | *rpsL1* ∆*cps29J*::P-*erm* ∆*bgaA*::P_Zn_-*cps2J* | ∆*cpsJ*::P-*erm* $\times$ NUS0901 | Erm^R^, Str^R^, Zn^2+^ | This study |
| NUS1236 | *rpsL1* ∆*cps2J*::P-*erm* ∆*bgaA*::P_Zn_-*cps2J* | ∆*cpsJ*::P-*erm* $\times$ NUS0063 | Erm^R^, Str^R^, Zn^2+^ | This study |
| NUS0282 | *rpsL1* CPS19A | CPS19A $\times$ NUS0114 | Str^R^ | This study |
| NUS0447 | *rpsL1* CPS19A ∆*bgaA*::P-*sacB*-*kan*-*rpsL^+^* | ∆*bgaA*::P-*sacB*-*kan*-*rpsL^+^*$\times$ NUS0282 | Kan^R^, Str^S^, Suc^S^ | This study |
| NUS1558 | *rpsL1* CPS19A ∆*bgaA*::P*_spxB_*-*cps19AJ* | ∆*bgaA*::P*_spxB_*-*cps19AJ* $\times$ NUS0447 | Str^R^ | This study |
| NUS1580 | *rpsL1* CPS19A ∆*cps19AJ*::P-*erm* ∆*bgaA*::P*_spxB_*-*cps19AJ* | ∆*cps19AJ*::P-*erm* $\times$ NUS1558 | Str^R^, Erm^R^ | This study |
| NUS0317 | *rpsL1* CPS19B | CPS19B $\times$ NUS0114 | Str^R^, Suc^R^ | This study |
| NUS1525 | *rpsL1* CPS19B ∆*bgaA*::P-*sacB*-*kan*-*rpsL^+^* | ∆*bgaA*::P-*sacB*-*kan*-*rpsL^+^*$\times$ NUS0317 | Kan^R^, Str^S^, Suc^S^ | This study |
| NUS1527 | *rpsL1* CPS19B ∆*bgaA*::P*_spxB_*-*cps19AJ* | ∆*bgaA*::P*_spxB_*-*cps19AJ* $\times$ NUS1525 | Str^R^ | This study |
| NUS0316 | *rpsL1* CPS19C | CPS19C $\times$ NUS0114 | Str^R^ | This study |
| NUS1568 | *rpsL1* CPS19C ∆*bgaA*::P-*sacB*-*kan*-*rpsL^+^* | ∆*bgaA*::P-*sacB*-*kan*-*rpsL^+^*$\times$ NUS0316 | Kan^R^, Str^S^, Suc^S^ | This study |
| NUS1584 | *rpsL1* CPS19C ∆*bgaA*::P*_spxB_*-*cps19AJ* | ∆*bgaA*::P*_spxB_*-*cps19AJ* $\times$ NUS1568 | Str^R^ | This study |
| NUS0329 | *rpsL1* CPS23B | CPS23B $\times$ NUS0114 | Str^R^ | This study |
| NUS1957 | *rpsL1* CPS23B ∆*bgaA*::P-*kan-cps23BJ^+^* | ∆*bgaA*::P-*kan*-*cps23BJ^+^*$\times$ NUS0329 | Kan^R^, Str^R^ | This study |
| NUS1958 | *rpsL1* CPS23B ∆*bgaA*::P-*kan-cps23BJ*(P30S) | ∆*bgaA*::P-*kan*-*cps23BJ*(P30S*)*$\times$ NUS0329 | Kan^R^, Str^R^ | This study |
| NUS1959 | *rpsL1* CPS23B ∆*bgaA*::P-*kan-cps23BJ(T33A)* | ∆*bgaA*::P-*kan*-*cps23BJ*(T33A*)*$\times$ NUS0329 | Kan^R^, Str^R^ | This study |
| NUS1960 | *rpsL1* CPS23B ∆*bgaA*::P-*kan-cps23BJ*(A152T) | ∆*bgaA*::P-*kan*-*cps23BJ*(A152T*)*$\times$ NUS0329 | Kan^R^, Str^R^ | This study |
| NUS1961 | *rpsL1* CPS23B ∆*bgaA*::P-*kan-cps23BJ*(S244G) | ∆*bgaA*::P-*kan*-*cps23BJ*(S244G)$\times$ NUS0329 | Kan^R^, Str^R^ | This study |
| NUS1962 | *rpsL1* CPS23B ∆*bgaA*::P-*kan-cps23BJ*(D231G) | ∆*bgaA*::P-*kan*-*cps23BJ*(D231G*)*$\times$ NUS0329 | Kan^R^, Str^R^ | This study |
| NUS1963 | *rpsL1* CPS23B ∆*bgaA*::P-*kan-cps23BJ*(P254S) | ∆*bgaA*::P-*kan*-*cps23BJ*(P254S*)*$\times$ NUS0329 | Kan^R^, Str^R^ | This study |
| NUS1964 | *rpsL1* CPS23B ∆*bgaA*::P-*kan-cps23BJ*(G316E) | ∆*bgaA*::P-*kan*-*cps23BJ*(G316E*)*$\times$ NUS0329 | Kan^R^, Str^R^ | This study |
| NUS1972 | *rpsL1* CPS19A ∆*bgaA*::*P_spxB_*-*cps19AJ*(D64A) | ∆*bgaA*::P*_spxB_*-*cps19AJ*(D64A) $\times$ NUS0447 | Str^R^ | This study |
| NUS1973 | *rpsL1* CPS19A ∆*bgaA*::*P_spxB_*-*cps19AJ*(G71R) | ∆*bgaA*::P*_spxB_*-*cps19AJ*(G71R) $\times$ NUS0447 | Str^R^ | This study |
| NUS1997 | *rpsL1* CPS23B ∆*cps23BJ*::P-*erm* ∆*bgaA*::P-*kan*-*cps23BJ*^+^ | ∆*cps23BJ*::P-*erm* $\times$ NUS1957 | Erm^R^, Kan^R^ | This study |
| NUS1998 | *rpsL1* CPS23B ∆*cps23BJ*::P-*erm* ∆*bgaA*::P-*kan*-*cps23BJ* (P30S) | ∆*cps23BJ*::P-*erm* $\times$ NUS1958 | Erm^R^, Kan^R^ | This study |
| NUS1999 | *rpsL1* CPS23B ∆*cps23BJ*::P-*erm* ∆*bgaA*::P-*kan*-*cps23BJ* (T33A) | ∆*cps23BJ*::P-*erm* $\times$ NUS1959 | Erm^R^, Kan^R^ | This study |
| NUS2000 | *rpsL1* CPS23B ∆*cps23BJ*::P-*erm* ∆*bgaA*::P-*kan*-*cps23BJ* (A152T) | ∆*cps23BJ*::P-*erm* $\times$ NUS1960 | Erm^R^, Kan^R^ | This study |
| NUS2001 | *rpsL1* CPS23B ∆*cps23BJ*::P-*erm* ∆*bgaA*::P-*kan*-*cps23BJ* (S244G) | ∆*cps23BJ*::P-*erm* $\times$ NUS1961 | Erm^R^, Kan^R^ | This study |
| NUS2002 | *rpsL1* CPS23B ∆*cps23BJ*::P-*erm* ∆*bgaA*::P-*kan*-*cps23BJ* (D231G) | ∆*cps23BJ*::P-*erm* $\times$ NUS1962 | Erm^R^, Kan^R^ | This study |
| NUS2003 | *rpsL1* CPS23B ∆*cps23BJ*::P-*erm* ∆*bgaA*::P-*kan*-*cps23BJ* (P254S) | ∆*cps23BJ*::P-*erm* $\times$ NUS1963 | Erm^R^, Kan^R^ | This study |
| NUS2004 | *rpsL1* CPS23B ∆*cps23BJ*::P-*erm* ∆*bgaA*::P-*kan*-*cps23BJ* (G316E) | ∆*cps23BJ*::P-*erm* $\times$ NUS1964 | Erm^R^, Kan^R^ | This study |
| NUS0312 | *rpsL1* CPS10A | CPS10A $\times$ NUS0114 | Str^R^ | This study |
| NUS2049 | *rpsL1* CPS10A ∆*bgaA*::P-*kan*-*cps10AJ^+^* | ∆*bgaA*::P-*kan*-*cps10AJ^+^*$\times$ NUS0312 | Kan^R^ | This study |
| NUS2050 | *rpsL1* CPS10A ∆*bgaA*::P-*kan*-*cps10AJ*(I101T) | ∆*bgaA*::P-*kan*-*cps10A*(I101T)$\times$ NUS0312 | Kan^R^ | This study |
| NUS2051 | *rpsL1* CPS10A ∆*bgaA*::P-*kan*-*cps10AJ*(F109V) | ∆*bgaA*::P-*kan*-*cps10AJ*(F109V)$\times$ NUS0312 | Kan^R^ | This study |
| NUS2052 | *rpsL1* CPS10A ∆*bgaA*::P-*kan*-*cps10AJ*(Y192H) | ∆*bgaA*::P-*kan*-*cps10AJ*(Y182H)$\times$ NUS0312 | Kan^R^ | This study |
| NUS2053 | *rpsL1* CPS10A ∆*bgaA*::P-*kan*-*cps10AJ*(E222N) | ∆*bgaA*::P-*kan*-*cps10AJ*(E222N)$\times$ NUS0312 | Kan^R^ | This study |
| NUS2054 | *rpsL1* CPS10A ∆*bgaA*::P-*kan*-*cps10AJ*(A356V) | ∆*bgaA*::P-*kan*-*cps10AJ*(A356V)$\times$ NUS0312 | Kan^R^ | This study |
| NUS2064 | *rpsL1* CPS19B ∆*CEP:*:P-*kan*-*cps19C(wchU)* | ∆*CEP*::P-*kan*-*cps19C(wchU)* x NUS0317 | Kan^R^ | This study |
| NUS2065 | *rpsL1* CPS19B ∆*CEP*::P-*kan*-*cps19C(wchU)* ∆*bgaA*::P*_spxB_*-*cps19AJ* | ∆*CEP*::P-*kan*-*cps19C(wchU)* x NUS1527 | Kan^R^ | This study |
| NUS2066 | *rpsL1* CPS19C P*_spxB_*-*cps19AJ*(D64A) | ∆*bgaA*::P*_spxB_*-*cps19AJ*(D64A) x NUS1568 | Str^R^ | This study |
| NUS2067 | *rpsL1* CPS19C P*_spxB_*-*cps19AJ*(G71R) | ∆*bgaA*::P*_spxB_*-*cps19AJ*(G71R) x NUS1568 | Str^R^ | This study |
| NUS2074 | *rpsL1* CPS10A ∆*cps10AJ*::P-*erm* ∆*bgaA*::P-*kan*-*cps10AJ^+^* | ∆*cps10AJ*::P-*erm* x NUS2049 | Erm^R^, Kan^R^ | This study |
| NUS2075 | *rpsL1* CPS10A ∆*cps10AJ*::P-*erm* ∆*bgaA*::P-*kan*-*cps10AJ*(I101T) | ∆*cps10AJ*::P-*erm* x NUS2050 | Erm^R^, Kan^R^ | This study |
| NUS2076 | *rpsL1* CPS10A ∆*cps10AJ*::P-*erm* ∆*bgaA*::P-*kan*-*cps10AJ*(F109V) | ∆*cps10AJ*::P-*erm* x NUS2051 | Erm^R^, Kan^R^ | This study |
| NUS2077 | *rpsL1* CPS10A ∆*cps10AJ*::P-*erm* ∆*bgaA*::P-*kan*-*cps10AJ*(Y192H) | ∆*cps10AJ*::P-*erm* x NUS2052 | Erm^R^, Kan^R^ | This study |
| NUS2078 | *rpsL1* CPS10A ∆*cps10AJ*::P-*erm* ∆*bgaA*::P-*kan*-*cps10AJ*(E222N) | ∆*cps10AJ*::P-*erm* x NUS2053 | Erm^R^, Kan^R^ | This study |
| NUS2079 | *rpsL1* CPS10A ∆*cps10AJ*::P-*erm* ∆*bgaA*::P-*kan*-*cps10AJ*(A356V) | ∆*cps10AJ*::P-*erm* x NUS2054 | Erm^R^, Kan^R^ | This study |
| NUS2066 | *rpsL1* CPS19C P*_spxB_*-*cps19AJ*(D64A) | ∆*bgaA*::P*_spxB_*-*cps19AJ*(D64A) x NUS1568 | Str^R^ | This study |
| NUS2067 | *rpsL1* CPS19C P*_spxB_*-*cps19AJ*(G71R) | ∆*bgaA*::P*_spxB_*-*cps19AJ*(G71R) x NUS1568 | Str^R^ | This study |
| NUS2508 | *rpsL1* CPS19C P*_spxB_*-*cps19AJ*-FLAG | ∆*bgaA*::P*_spxB_*-*cps19AJ*-FLAG x NUS1568 | Str^R^ | This study |
| NUS2509 | *rpsL1* CPS19C P*_spxB_*-*cps19AJ(D64A)*-FLAG | ∆*bgaA*::P*_spxB_*-*cps19AJ*(D64A)-FLAG x NUS1568 | Str^R^ | This study |
| NUS2510 | *rpsL1* CPS19C P*_spxB_*-*cps19AJ(G71R)*-FLAG | ∆*bgaA*::P*_spxB_*-*cps19AJ*(G71R)-FLAG x NUS1568 | Str^R^ | This study |
| **Clinical isolates** | | | | |
| NUH0002 | Serotype 6A | - | - | NUH |
| NUH0003 | Serotype 6B | - | - | NUH |
| NUH0004 | Serotype 6C | - | - | NUH |
| NUH0005 | Serotype 7F | - | - | NUH |
| NUH0006 | Serotype 8 | - | - | NUH |
| NUH0007 | Serotype 14 | - | - | NUH |
| NUH0008 | Serotype 15A | - | - | NUH |
| NUH0009 | Serotype 15B | - | - | NUH |
| NUH0010 | Serotype 15C | - | - | NUH |
| NUH0011 | Serotype 15F | - | - | NUH |
| NUH0012 | Serotype 18C | - | - | NUH |
| NUH0013 | Serotype 19A | - | - | NUH |
| NUH0014 | Serotype 19F | - | - | NUH |
| NUH0015 | Serotype 22A | - | - | NUH |
| NUH0016 | Serotype 23A | - | - | NUH |
| NUH0017 | Serotype 23F | - | - | NUH |
| PATH18 | Serotype 31 | - | - | CDC |
| PATH20 | Serotype 24F | - | - | CDC |
| PATH30 | Serotype 9V | - | - | CDC |
| PATH46 | Serotype 5 | - | - | CDC |
| PATH51 | Serotype 35B | - | - | CDC |
| PATH57 | Serotype 21 | - | - | CDC |
| PATH89 | Serotype 11A | - | - | CDC |
| PATH98 | Serotype 9N | - | - | CDC |
| PATH101 | Serotype 33F | - | - | CDC |
| PATH106 | Serotype 1 | - | - | CDC |
| PATH112 | Serotype 38 | - | - | CDC |
| PATH115 | Serotype 22F | - | - | CDC |
| PATH122 | Serotype 12F | - | - | CDC |
| PATH203 | Serotype 7C | - | - | CDC |
| PATH212 | Serotype 23B | - | - | CDC |
| PATH269 | Serotype 18B | - | - | CDC |
| PATH344 | Serotype 33C | - | - | CDC |
| PATH352 | Serotype 25F | - | - | CDC |
| PATH382 | Serotype 28F | - | - | CDC |
| PATH656 | Serotype 45 | - | - | CDC |
| PATH676 | Serotype 35F | - | - | CDC |
| PATH680 | Serotype 17F | - | - | CDC |
| PATH682 | Serotype 20 | - | - | CDC |
| PATH691 | Serotype 10A | - | - | CDC |
| PATH1005 | Serotype 11C | - | - | CDC |
| PATH1539 | Serotype 10F | - | - | CDC |
| PATH1702 | Serotype 16F | - | - | CDC |
| PATH1706 | Serotype 12A | - | - | CDC |
| PATH1709 | Serotype 35A | - | - | CDC |
| PATH1754 | Serotype 33A | - | - | CDC |
| PATH1803 | Serotype 7B | - | - | CDC |
| PATH1833 | Serotype 36 | - | - | CDC |
| PATH1886 | Serotype 13 | - | - | CDC |
| PATH1895 | Serotype 35C | - | - | CDC |
| PATH1937 | Serotype 48 | - | - | CDC |
| PATH1945 | Serotype 33B | - | - | CDC |
| PATH2009 | Serotype 39 | - | - | CDC |
| PATH2459 | Serotype 10B | - | - | CDC |
| PATH2460 | Serotype 10C | - | - | CDC |
| PATH2461 | Serotype 11F | - | - | CDC |
| PATH2462 | Serotype 11D | - | - | CDC |
| PATH2463 | Serotype 19C | - | - | CDC |
| PATH2464 | Serotype 24A | - | - | CDC |
| PATH2465 | Serotype 24B | - | - | CDC |
| PATH2466 | Serotype 25A | - | - | CDC |
| PATH2467 | Serotype 27 | - | - | CDC |
| PATH2468 | Serotype 32F | - | - | CDC |
| PATH2469 | Serotype 40 | - | - | CDC |
| PATH2470 | Serotype 41F | - | - | CDC |
| PATH2471 | Serotype 41A | - | - | CDC |
| PATH2472 | Serotype 43 | - | - | CDC |
| PATH2473 | Serotype 44 | - | - | CDC |
| PATH2474 | Serotype 46 | - | - | CDC |
| PATH2475 | Serotype 47F | - | - | CDC |
| PATH2476 | Serotype 47A | - | - | CDC |
| PATH2477 | Serotype 7A | - | - | CDC |
| PATH2478 | Serotype 9L | - | - | CDC |
| PATH2479 | Serotype 12B | - | - | CDC |
| PATH2480 | Serotype 17A | - | - | CDC |
| PATH2481 | Serotype 33D | - | - | CDC |
| PATH2606 | Serotype 19B | - | - | CDC |
| PATH3390 | Serotype 6D | - | - | CDC |
| PATH4478 | Serotype 29 | - | - | CDC |
| PATH4559 | Serotype 11B | - | - | CDC |
| PATH4560 | Serotype 18A | - | - | CDC |
| PATH4599 | Serotype 18F | - | - | CDC |
| PATH4969 | Serotype 9A | - | - | CDC |
| PATH6653 | Serotype 32A | - | - | CDC |
| PATH6745 | Serotype 16A | - | - | CDC |
| PATH9002 | Serotype 28A | - | - | CDC |
| CCUG2399 | Serotype 34 | - | - | CCUG |
| CCUG6568 | Serotype 42 | - | - | CCUG |
| CCUG37285 | Serotype 4 | - | - | CCUG |
| **Flippase-swapped mutants** | | | | |
| NUS0088 | *rpsL1* *∆cps2J<>cps15AJ* ∆*bgaA*::P_Zn_-*cps2J* | *∆cps2J<>cps15AJ* $\times$ NUS0064 | Str^R^, Zn^2+^ | This study |
| NUS0089 | *rpsL1* *∆cps2J<>cps23FJ* ∆*bgaA*::P_Zn_-*cps2J* | *∆cps2J<>cps23FJ* $\times$ NUS0064 | Str^R^, Zn^2+^ | This study |
| NUS0094 | *rpsL1* *∆cps2J<>cps35BJ* ∆*bgaA*::P_Zn_-*cps2J* | *∆cps2J<>cps35BJ* $\times$ NUS0064 | Str^R^, Zn^2+^ | This study |
| NUS0095 | *rpsL1* *∆cps2J<>cps8J* ∆*bgaA*::P_Zn_-*cps2J* | *∆cps2J<>cps8J* $\times$ NUS0064 | Str^R^, Zn^2+^ | This study |
| NUS0096 | *rpsL1* *∆cps2J<>cps34J* ∆*bgaA*::P_Zn_-*cps2J* | *∆cps2J<>cps34J* $\times$ NUS0064 | Str^R^, Zn^2+^ | This study |
| NUS0098 | *rpsL1* *∆cps2J<>cps14J* ∆*bgaA*::P_Zn_-*cps2J* | *∆cps2J<>cps14J* $\times$ NUS0064 | Str^R^, Zn^2+^ | This study |
| NUS0099 | *rpsL1* *∆cps2J<>cps6AJ* ∆*bgaA*::P_Zn_-*cps2J* | *∆cps2J<>cps6AJ* $\times$ NUS0064 | Str^R^, Zn^2+^ | This study |
| NUS0141 | *rpsL1* *∆cps2J<>cps23BJ* ∆*bgaA*::P_Zn_-*cps2J* | *∆cps2J<>cps23BJ* $\times$ NUS0064 | Str^R^, Zn^2+^ | This study |
| NUS0142 | *rpsL1* *∆cps2J<>cps33CJ* ∆*bgaA*::P_Zn_-*cps2J* | *∆cps2J<>cps33CJ* $\times$ NUS0064 | Str^R^, Zn^2+^ | This study |
| NUS0143 | *rpsL1* *∆cps2J<>cps35AJ* ∆*bgaA*::P_Zn_-*cps2J* | *∆cps2J<>cps35AJ* $\times$ NUS0064 | Str^R^, Zn^2+^ | This study |
| NUS0266 | *rpsL1* *∆cps2J<>cps33BJ* ∆*bgaA*::P_Zn_-*cps2J* | *∆cps2J<>cps35BJ* $\times$ NUS0064 | Str^R^, Zn^2+^ | This study |
| NUS0356 | *rpsL1* *∆cps2J<>cps33AJ* ∆*bgaA*::P_Zn_-*cps2J* | *∆cps2J<>cps33AJ* $\times$ NUS0064 | Str^R^, Zn^2+^ | This study |
| NUS0362 | *rpsL1* *∆cps2J<>cps33DJ* ∆*bgaA*::P_Zn_-*cps2J* | *∆cps2J<>cps33DJ* $\times$ NUS0064 | Str^R^, Zn^2+^ | This study |
| NUS0363 | *rpsL1* *∆cps2J<>cps33FJ* ∆*bgaA*::P_Zn_-*cps2J* | *∆cps2J<>cps33FJ* $\times$ NUS0064 | Str^R^, Zn^2+^ | This study |
| NUS0455 | *rpsL1* *∆cps2J<>cps10FJ* ∆*bgaA*::P_Zn_-*cps2J* | *∆cps2J<>cps10FJ* $\times$ NUS0064 | Str^R^, Zn^2+^ | This study |
| NUS0456 | *rpsL1* *∆cps2J<>cps10CJ* ∆*bgaA*::P_Zn_-*cps2J* | *∆cps2J<>cps10CJ* $\times$ NUS0064 | Str^R^, Zn^2+^ | This study |
| NUS0457 | *rpsL1* *∆cps2J<>cps13J* ∆*bgaA*::P_Zn_-*cps2J* | *∆cps2J<>cps13J* $\times$ NUS0064 | Str^R^, Zn^2+^ | This study |
| NUS0489 | *rpsL1* *∆cps2J<>cps9AJ* ∆*bgaA*::P_Zn_-*cps2J* | *∆cps2J<>cps9AJ* $\times$ NUS0064 | Str^R^, Zn^2+^ | This study |
| NUS0527 | *rpsL1* *∆cps2J<>cps5J* ∆*bgaA*::P_Zn_-*cps2J* | *∆cps2J<>cps5J* $\times$ NUS0064 | Str^R^, Zn^2+^ | This study |
| NUS0528 | *rpsL1* ∆*cps2J<>cps9LJ* ∆*bgaA*::P_Zn_-*cps2J* | *∆cps2J<>cps9LJ* $\times$ NUS0064 | Str^R^, Zn^2+^ | This study |
| NUS0529 | *rpsL1* ∆*cps2J<>cps23AJ* ∆*bgaA*::P_Zn_-*cps2J* | *∆cps2J<>cps23AJ* $\times$ NUS0064 | Str^R^, Zn^2+^ | This study |
| NUS0531 | *rpsL1* ∆*cps2J<>cps7BJ* ∆*bgaA*::P_Zn_-*cps2J* | *∆cps2J<>cps7BJ* $\times$ NUS0064 | Str^R^, Zn^2+^ | This study |
| NUS0538 | *rpsL1* ∆*cps2J<>cps1J* ∆*bgaA*::P_Zn_-*cps2J* | *∆cps2J<>cps1J* $\times$ NUS0064 | Str^R^, Zn^2+^ | This study |
| NUS0539 | *rpsL1* ∆*cps2J<>cps7AJ* ∆*bgaA*::P_Zn_-*cps2J* | *∆cps2J<>cps7AJ* $\times$ NUS0064 | Str^R^, Zn^2+^ | This study |
| NUS0540 | *rpsL1* ∆*cps2J<>cps7CJ* ∆*bgaA*::P_Zn_-*cps2J* | *∆cps2J<>cps7CJ* $\times$ NUS0064 | Str^R^, Zn^2+^ | This study |
| NUS0541 | *rpsL1* ∆*cps2J<>cps7FJ* ∆*bgaA*::P_Zn_-*cps2J* | *∆cps2J<>cps7FJ* $\times$ NUS0064 | Str^R^, Zn^2+^ | This study |
| NUS0549 | *rpsL1* ∆*cps2J<>cps10AJ* ∆*bgaA*::P_Zn_-*cps2J* | *∆cps2J<>cps10AJ* $\times$ NUS0064 | Str^R^, Zn^2+^ | This study |
| NUS0550 | *rpsL1* ∆*cps2J<>cps10BJ* ∆*bgaA*::P_Zn_-*cps2J* | *∆cps2J<>cps10BJ* $\times$ NUS0064 | Str^R^, Zn^2+^ | This study |
| NUS0551 | *rpsL1* ∆*cps2J<>cps39J* ∆*bgaA*::P_Zn_-*cps2J* | *∆cps2J<>cps39J* $\times$ NUS0064 | Str^R^, Zn^2+^ | This study |
| NUS0577 | *rpsL1* ∆*cps2J<>cps15BJ* ∆*bgaA*::P_Zn_-*cps2J* | *∆cps2J<>cps15BJ* $\times$ NUS0064 | Str^R^, Zn^2+^ | This study |
| NUS0578 | *rpsL1* ∆*cps2J<>cps35CJ* ∆*bgaA*::P_Zn_-*cps2J* | *∆cps2J<>cps35CJ* $\times$ NUS0064 | Str^R^, Zn^2+^ | This study |
| NUS0579 | *rpsL1* ∆*cps2J<>cps15FJ* ∆*bgaA*::P_Zn_-*cps2J* | *∆cps2J<>cps15FJ* $\times$ NUS0064 | Str^R^, Zn^2+^ | This study |
| NUS0580 | *rpsL1* ∆*cps2J<>cps15CJ* ∆*bgaA*::P_Zn_-*cps2J* | *∆cps2J<>cps15CJ* $\times$ NUS0064 | Str^R^, Zn^2+^ | This study |
| NUS0581 | *rpsL1* ∆*cps2J<>cps36J* ∆*bgaA*::P_Zn_-*cps2J* | *∆cps2J<>cps36J* $\times$ NUS0064 | Str^R^, Zn^2+^ | This study |
| NUS0641 | *rpsL1* ∆*cps2J<>cps31J* ∆*bgaA*::P_Zn_-*cps2J* | *∆cps2J<>cps31J* $\times$ NUS0064 | Str^R^, Zn^2+^ | This study |
| NUS0642 | *rpsL1* ∆*cps2J<>cps41AJ* ∆*bgaA*::P_Zn_-*cps2J* | *∆cps2J<>cps41AJ* $\times$ NUS0064 | Str^R^, Zn^2+^ | This study |
| NUS0643 | *rpsL1* ∆*cps2J<>cps41FJ* ∆*bgaA*::P_Zn_-*cps2J* | *∆cps2J<>cps41FJ* $\times$ NUS0064 | Str^R^, Zn^2+^ | This study |
| NUS0644 | *rpsL1* ∆*cps2J<>cps43J* ∆*bgaA*::P_Zn_-*cps2J* | *∆cps2J<>cps43J* $\times$ NUS0064 | Str^R^, Zn^2+^ | This study |
| NUS0645 | *rpsL1* ∆*cps2J<>cps45J* ∆*bgaA*::P_Zn_-*cps2J* | *∆cps2J<>cps45J* $\times$ NUS0064 | Str^R^, Zn^2+^ | This study |
| NUS0652 | *rpsL1* ∆*cps2J<>cps32AJ* ∆*bgaA*::P_Zn_-*cps2J* | *∆cps2J<>cps32AJ* $\times$ NUS0064 | Str^R^, Zn^2+^ | This study |
| NUS0653 | *rpsL1* ∆*cps2J<>cps32FJ* ∆*bgaA*::P_Zn_-*cps2J* | *∆cps2J<>cps32FJ* $\times$ NUS0064 | Str^R^, Zn^2+^ | This study |
| NUS0654 | *rpsL1* ∆*cps2J<>cps40J* ∆*bgaA*::P_Zn_-*cps2J* | *∆cps2J<>cps40J* $\times$ NUS0064 | Str^R^, Zn^2+^ | This study |
| NUS0655 | *rpsL1* ∆*cps2J<>cps44J* ∆*bgaA*::P_Zn_-*cps2J* | *∆cps2J<>cps44J* $\times$ NUS0064 | Str^R^, Zn^2+^ | This study |
| NUS0656 | *rpsL1* ∆*cps2J<>cps46J* ∆*bgaA*::P_Zn_-*cps2J* | *∆cps2J<>cps46J* $\times$ NUS0064 | Str^R^, Zn^2+^ | This study |
| NUS0657 | *rpsL1* ∆*cps2J<>cps47AJ* ∆*bgaA*::P_Zn_-*cps2J* | *∆cps2J<>cps47AJ* $\times$ NUS0064 | Str^R^, Zn^2+^ | This study |
| NUS0658 | *rpsL1* ∆*cps2J<>cps47FJ* ∆*bgaA*::P_Zn_-*cps2J* | *∆cps2J<>cps47FJ* $\times$ NUS0064 | Str^R^, Zn^2+^ | This study |
| NUS0659 | *rpsL1* ∆*cps2J<>cps48J* ∆*bgaA*::P_Zn_-*cps2J* | *∆cps2J<>cps48J* $\times$ NUS0064 | Str^R^, Zn^2+^ | This study |
| NUS0694 | *rpsL1* ∆*cps2J<>cps38J* ∆*bgaA*::P_Zn_-*cps2J* | *∆cps2J<>cps38J* $\times$ NUS0064 | Str^R^, Zn^2+^ | This study |
| NUS0695 | *rpsL1* ∆*cps2J<>cps25FJ* ∆*bgaA*::P_Zn_-*cps2J* | *∆cps2J<>cps25FJ* $\times$ NUS0064 | Str^R^, Zn^2+^ | This study |
| NUS0760 | *rpsL1* ∆*cps2J<>cps25AJ* ∆*bgaA*::P_Zn_-*cps2J* | *∆cps2J<>cps25AJ* $\times$ NUS0064 | Str^R^, Zn^2+^ | This study |
| NUS0761 | *rpsL1* ∆*cps2J<>cps24BJ* ∆*bgaA*::P_Zn_-*cps2J* | *∆cps2J<>cps24BJ* $\times$ NUS0064 | Str^R^, Zn^2+^ | This study |
| NUS0762 | *rpsL1* ∆*cps2J<>cps24FJ* ∆*bgaA*::P_Zn_-*cps2J* | *∆cps2J<>cps24FJ* $\times$ NUS0064 | Str^R^, Zn^2+^ | This study |
| NUS0774 | *rpsL1* ∆*cps2J<>cps6BJ* ∆*bgaA*::P_Zn_-*cps2J* | *∆cps2J<>cps6BJ* $\times$ NUS0064 | Str^R^, Zn^2+^ | This study |
| NUS0775 | *rpsL1* ∆*cps2J<>cps6CJ* ∆*bgaA*::P_Zn_-*cps2J* | *∆cps2J<>cps6CJ* $\times$ NUS0064 | Str^R^, Zn^2+^ | This study |
| NUS0776 | *rpsL1* ∆*cps2J<>cps6DJ* ∆*bgaA*::P_Zn_-*cps2J* | *∆cps2J<>cps6DJ* $\times$ NUS0064 | Str^R^, Zn^2+^ | This study |
| NUS0783 | *rpsL1* ∆*cps2J<>cps9VJ* ∆*bgaA*::P_Zn_-*cps2J* | *∆cps2J<>cps9VJ* $\times$ NUS0064 | Str^R^, Zn^2+^ | This study |
| NUS0784 | *rpsL1* ∆*cps2J<>cps9NJ* ∆*bgaA*::P_Zn_-*cps2J* | *∆cps2J<>cps9NJ* $\times$ NUS0064 | Str^R^, Zn^2+^ | This study |
| NUS0835 | *rpsL1* *∆cps2J<>cps20J* ∆*bgaA*::P_Zn_-*cps2J* | *∆cps2J<>cps20J* $\times$ NUS0064 | Str^R^, Zn^2+^ | This study |
| NUS0836 | *rpsL1* *∆cps2J<>cps21J* ∆*bgaA*::P_Zn_-*cps2J* | *∆cps2J<>cps21J* $\times$ NUS0064 | Str^R^, Zn^2+^ | This study |
| NUS0837 | *rpsL1* ∆*cps2J<>cps22AJ* ∆*bgaA*::P_Zn_-*cps2J* | *∆cps2J<>cps22AJ* $\times$ NUS0064 | Str^R^, Zn^2+^ | This study |
| NUS0838 | *rpsL1* ∆*cps2J<>cps22FJ* ∆*bgaA*::P_Zn_-*cps2J* | *∆cps2J<>cps22FJ* $\times$ NUS0064 | Str^R^, Zn^2+^ | This study |
| NUS0839 | *rpsL1* ∆*cps2J<>cps27J* ∆*bgaA*::P_Zn_-*cps2J* | *∆cps2J<>cps27J* $\times$ NUS0064 | Str^R^, Zn^2+^ | This study |
| NUS0840 | *rpsL1* ∆*cps2J<>cps28AJ* ∆*bgaA*::P_Zn_-*cps2J* | *∆cps2J<>cps28AJ* $\times$ NUS0064 | Str^R^, Zn^2+^ | This study |
| NUS0841 | *rpsL1* ∆*cps2J<>cps28FJ* ∆*bgaA*::P_Zn_-*cps2J* | *∆cps2J<>cps28FJ* $\times$ NUS0064 | Str^R^, Zn^2+^ | This study |
| NUS0854 | *rpsL1* *∆cps2J<>cps12AJ* ∆*bgaA*::P_Zn_-*cps2J* | *∆cps2J<>cps12AJ* $\times$ NUS0064 | Str^R^, Zn^2+^ | This study |
| NUS0855 | *rpsL1* ∆*cps2J<>cps12BJ* ∆*bgaA*::P_Zn_-*cps2J* | *∆cps2J<>cps12BJ* $\times$ NUS0064 | Str^R^, Zn^2+^ | This study |
| NUS0856 | *rpsL1* ∆*cps2J<>cps12FJ* ∆*bgaA*::P_Zn_-*cps2J* | *∆cps2J<>cps12FJ* $\times$ NUS0064 | Str^R^, Zn^2+^ | This study |
| NUS0866 | *rpsL1* ∆*cps2J<>cps18BJ* ∆*bgaA*::P_Zn_-*cps2J* | *∆cps2J<>cps18BJ* $\times$ NUS0064 | Str^R^, Zn^2+^ | This study |
| NUS0867 | *rpsL1* ∆*cps2J<>cps18CJ* ∆*bgaA*::P_Zn_-*cps2J* | *∆cps2J<>cps18CJ* $\times$ NUS0064 | Str^R^, Zn^2+^ | This study |
| NUS0868 | *rpsL1* ∆*cps2J<>cps18FJ* ∆*bgaA*::P_Zn_-*cps2J* | *∆cps2J<>cps18FJ* $\times$ NUS0064 | Str^R^, Zn^2+^ | This study |
| NUS0869 | *rpsL1* ∆*cps2J<>cps19AJ* ∆*bgaA*::P_Zn_-*cps2J* | *∆cps2J<>cps19AJ* $\times$ NUS0064 | Str^R^, Zn^2+^ | This study |
| NUS0870 | *rpsL1* ∆*cps2J<>cps18AJ* ∆*bgaA*::P_Zn_-*cps2J* | *∆cps2J<>cps18AJ* $\times$ NUS0064 | Str^R^, Zn^2+^ | This study |
| NUS0871 | *rpsL1* ∆*cps2J<>cps19FJ* ∆*bgaA*::P_Zn_-*cps2J* | *∆cps2J<>cps19FJ* $\times$ NUS0064 | Str^R^, Zn^2+^ | This study |
| NUS0874 | *rpsL1* ∆*cps2J<>cps19BJ* ∆*bgaA*::P_Zn_-*cps2J* | *∆cps2J<>cps19BJ* $\times$ NUS0064 | Str^R^, Zn^2+^ | This study |
| NUS0875 | *rpsL1* ∆*cps2J<>cps19CJ* ∆*bgaA*::P_Zn_-*cps2J* | *∆cps2J<>cps19CJ* $\times$ NUS0064 | Str^R^, Zn^2+^ | This study |
| NUS0877 | *rpsL1* ∆*cps2J<>cps16FJ* ∆*bgaA*::P_Zn_-*cps2J* | *∆cps2J<>cps16FJ* $\times$ NUS0064 | Str^R^, Zn^2+^ | This study |
| NUS0878 | *rpsL1* ∆*cps2J<>cps17AJ* ∆*bgaA*::P_Zn_-*cps2J* | *∆cps2J<>cps17AJ* $\times$ NUS0064 | Str^R^, Zn^2+^ | This study |
| NUS0879 | *rpsL1* ∆*cps2J<>cps17FJ* ∆*bgaA*::P_Zn_-*cps2J* | *∆cps2J<>cps17FJ* $\times$ NUS0064 | Str^R^, Zn^2+^ | This study |
| NUS0886 | *rpsL1* ∆*cps2J<>cps24AJ* ∆*bgaA*::P_Zn_-*cps2J* | *∆cps2J<>cps24AJ* $\times$ NUS0064 | Str^R^, Zn^2+^ | This study |
| NUS0900 | *rpsL1* ∆*cps2J<>cps4J* ∆*bgaA*::P_Zn_-*cps2J* | *∆cps2J<>cps4J* $\times$ NUS0064 | Str^R^, Zn^2+^ | This study |
| NUS0901 | *rpsL1* ∆*cps2J<>cps29J* ∆*bgaA*::P_Zn_-*cps2J* | *∆cps2J<>cps29J* $\times$ NUS0064 | Str^R^, Zn^2+^ | This study |
| NUS1110 | *rpsL1* ∆*cps2J<>cps42J* ∆*bgaA*::P_Zn_-*cps2J* | *∆cps2J<>cps42J* $\times$ NUS0064 | Str^R^, Zn^2+^ | This study |
| NUS0723 | *rpsL1* ∆*cps2J*<>*tacF* ∆*bgaA*::P_Zn_-*cps2J* | *∆cps2J<>tacF* $\times$ NUS0650 | Str^R^, Zn^2+^ | This study |
| NUS0759 | *rpsL1* ∆*cps2J*<>*ytgP* ∆*bgaA*::P_Zn_-*cps2J* | *∆cps2J<>ytgP* $\times$ NUS0650 | Str^R^, Zn^2+^ | This study |
| NUS0763 | *rpsL1* ∆*tacF*::P-*erm* ∆*cps2J*<>*tacF* ∆*bgaA*::P_Zn_-*cps2J* | ∆*tacF*::P-*erm* $\times$ NUS0723 | Erm^R^, Str^R^, Zn^2+^ | This study |
| NUS1013 | *rpsL1* ∆*ytgP*::P-*erm* ∆*cps2J*<>*ytgP* ∆*bgaA*::P_Zn_-*cps2J* | ∆*ytgP*::P-*erm* $\times$ NUS0759 | Erm^R^, Str^R^, Zn^2+^ | This study |
| NUS0112 | *rpsL1 ∆cps2J<>cps34J ∆bgaA::*P*-erm* | *∆bgaA::*P*-erm* $\times$ NUS0096 | Erm^R^, Str^R^ | This study |
| NUS0286 | *rpsL1* ∆*cps2J*<>*cps33BJ* ∆*bgaA*::P*-erm* | *∆bgaA::*P*-erm* $\times$ NUS0266 | Erm^R^, Str^R^ | This study |
| NUS0287 | *rpsL1* ∆*cps2J*<>*cps33CJ* ∆*bgaA*::P*-erm* | *∆bgaA::*P*-erm* $\times$ NUS0142 | Erm^R^, Str^R^ | This study |
| NUS0947 | *rpsL1* ∆*cps2J*<>*cps7AJ* ∆*bgaA*::P*-erm* | *∆bgaA::*P*-erm* $\times$ NUS0539 | Erm^R^, Str^R^ | This study |
| NUS0948 | *rpsL1* ∆*cps2J*<>*cps33DJ* ∆*bgaA*::P*-erm* | *∆bgaA::*P*-erm* $\times$ NUS0362 | Erm^R^, Str^R^ | This study |
| NUS0949 | *rpsL1* ∆*cps2J*<>*cps10BJ* ∆*bgaA*::P*-erm* | *∆bgaA::*P*-erm* $\times$ NUS0520 | Erm^R^, Str^R^ | This study |
| NUS0950 | *rpsL1* ∆*cps2J*<>*cps20J* ∆*bgaA*::P*-erm* | *∆bgaA::*P*-erm* $\times$ NUS0835 | Erm^R^, Str^R^ | This study |
| NUS0951 | *rpsL1* ∆*cps2J*<>*cps29J* ∆*bgaA*::P*-erm* | *∆bgaA::*P*-erm* $\times$ NUS0901 | Erm^R^, Str^R^ | This study |
| NUS0997 | *rpsL1* ∆*cps2J*<>*cps47FJ* ∆*bgaA*::P*-erm* | *∆bgaA::*P*-erm* $\times$ NUS0658 | Erm^R^, Str^R^ | This study |
| NUS0785 | *rpsL1* ∆*cps2J*<>*cps10AJ-*FLAG ∆*bgaA*::P_Zn_-*cps2J* | ∆*cps2J*<>*cps10AJ-*Flag $\times$ NUS0064 | Str^R^, Zn^2+^ | This study |
| NUS0786 | *rpsL1* ∆*cps2J*<>*cps10BJ-*FLAG ∆*bgaA*::P_Zn_-*cps2J* | ∆*cps2J*<>*cps10BJ-*Flag $\times$ NUS0064 | Str^R^, Zn^2+^ | This study |
| NUS0787 | *rpsL1* ∆*cps2J*<>*cps7AJ*-FLAG ∆*bgaA*::P_Zn_-*cps2J* | ∆*cps2J*<>*cps7AJ*-Flag $\times$ NUS0064 | Str^R^, Zn^2+^ | This study |
| NUS0831 | *rpsL1* ∆*cps2J*<>*cps23FJ*-FLAG ∆*bgaA*::P_Zn_-*cps2J* | ∆*cps2J*<>*cps23FJ*-Flag $\times$ NUS0064 | Str^R^, Zn^2+^ | This study |
| NUS0832 | *rpsL1* ∆*cps2J*<>*cps33BJ*-FLAG ∆*bgaA*::P_Zn_-*cps2J* | ∆*cps2J*<>*cps33BJ*-Flag $\times$ NUS0064 | Str^R^, Zn^2+^ | This study |
| NUS0833 | *rpsL1* ∆*cps2J*<>*cps48J*-FLAG ∆*bgaA*::P_Zn_-*cps2J* | ∆*cps2J*<>*cps48J*-Flag $\times$ NUS0064 | Str^R^, Zn^2+^ | This study |
| NUS0880 | *rpsL1* ∆*cps2J*<>*cps2J*-FLAG ∆*bgaA*::P_Zn_-*cps2J* | ∆*cps2J*<>*cps2J*-Flag $\times$ NUS0064 | Str^R^, Zn^2+^ | This study |
| NUS0697 | *rpsL1* ∆*cps2J*<>*cps10AJ* (I101T) ∆*bgaA*::P_Zn_-*cps2J* | ∆*cps2J*<>*cps10AJ* (I101T) $\times$ NUS0650 | Str^R^, Zn^2+^ | This study |
| NUS0698 | *rpsL1* ∆*cps2J*<>*cps10AJ* (F109V) ∆*bgaA*::P_Zn_-*cps2J* | ∆*cps2J*<>*cps10AJ* (F109V)$\times$ NUS0650 | Str^R^, Zn^2+^ | This study |
| NUS0699 | *rpsL1* ∆*cps2J*<>*cps10AJ* (Y192H) ∆*bgaA*::P_Zn_-*cps2J* | ∆*cps2J*<>*cps10AJ* (Y192H)$\times$ NUS0650 | Str^R^, Zn^2+^ | This study |
| NUS0700 | *rpsL1* ∆*cps2J*<>*cps10AJ* (E222N) ∆*bgaA*::P_Zn_-*cps2J* | ∆*cps2J*<>*cps10AJ* (E222N)$\times$ NUS0650 | Str^R^, Zn^2+^ | This study |
| NUS0701 | *rpsL1* ∆*cps2J*<>*cps10AJ* (A356V) ∆*bgaA*::P_Zn_-*cps2J* | ∆*cps2J*<>*cps10AJ* (A356V)$\times$ NUS0650 | Str^R^, Zn^2+^ | This study |
| NUS0726 | *rpsL1* ∆*cps2J*<>*cps10BJ* (T101I) ∆*bgaA*::P_Zn_-*cps2J* | ∆*cps2J*<>*cps10BJ* (T101I) $\times$ NUS0650 | Str^R^, Zn^2+^ | This study |
| NUS0727 | *rpsL1* ∆*cps2J*<>*cps10BJ* (V109F) ∆*bgaA*::P_Zn_-*cps2J* | ∆*cps2J*<>*cps10BJ* (V109F)$\times$ NUS0650 | Str^R^, Zn^2+^ | This study |
| NUS0728 | *rpsL1* ∆*cps2J*<>*cps10BJ* (H192Y) ∆*bgaA*::P_Zn_-*cps2J* | ∆*cps2J*<>*cps10BJ* (H192Y)$\times$ NUS0650 | Str^R^, Zn^2+^ | This study |
| NUS0729 | *rpsL1* ∆*cps2J*<>*cps10BJ* (N222E) ∆*bgaA*::P_Zn_-*cps2J* | ∆*cps2J*<>*cps10BJ* (N222E)$\times$ NUS0650 | Str^R^, Zn^2+^ | This study |
| NUS0730 | *rpsL1* ∆*cps2J*<>*cps10BJ* (V356A) ∆*bgaA*::P_Zn_-*cps2J* | ∆*cps2J*<>*cps10BJ* (V356A)$\times$ NUS0650 | Str^R^, Zn^2+^ | This study |
| NUS0857 | *rpsL1* ∆*cps2J*<>*cps10AJ*(I101T_Y192H) ∆*bgaA*::P_Zn_-*cps2J* | ∆*cps2J*<>*cps10AJ*(I101T_Y192H) $\times$ NUS0650 | Str^R^, Zn^2+^ | This study |
| NUS0858 | *rpsL1* ∆*cps2J*<>*cps10AJ*(I101T_A356V) ∆*bgaA*::P_Zn_-*cps2J* | ∆*cps2J*<>*cps10AJ*(I101T_A356V) $\times$ NUS0650 | Str^R^, Zn^2+^ | This study |
| NUS0881 | *rpsL1* ∆*cps2J*<>*cps10AJ*(F109V_E222N) ∆*bgaA*::P_Zn_-*cps2J* | ∆*cps2J*<>*cps10AJ*(F109V_E222N)$\times$ NUS0650 | Str^R^, Zn^2+^ | This study |
| NUS1069 | *rpsL1* ∆*cps2J*<>*cps10AJ*(I101T_F109V) ∆*bgaA*::P_Zn_-*cps2J* | ∆*cps2J*<>*cps10AJ*(I101T_F109V)$\times$ NUS0650 | Str^R^, Zn^2+^ | This study |
| NUS1120 | *rpsL1* ∆*cps2J*<>*cps10AJ*(F109V/A356V) ∆*bgaA*::P_Zn_-*cps2J* | ∆*cps2J*<>*cps10AJ*(F109V/A356V)$\times$ NUS0650 | Str^R^, Zn^2+^ | This study |
| NUS1121 | *rpsL1* ∆*cps2J*<>*cps10AJ*(E222N/A356V) ∆*bgaA*::P_Zn_-*cps2J* | ∆*cps2J*<>*cps10AJ*(E222N/A356V)$\times$ NUS0650 | Str^R^, Zn^2+^ | This study |
| NUS1142 | *rpsL1* ∆*cps2J*<>*cps10AJ*(I101T_E222N) ∆*bgaA*::P_Zn_-*cps2J* | ∆*cps2J*<>*cps10AJ*(I101T_E222N)$\times$ NUS0650 | Str^R^, Zn^2+^ | This study |
| NUS1981 | *rpsL1* *∆cps2J<>cps2J* (G43R)-FLAG ∆*bgaA*::P_Zn_-*cps2J* | ∆*cps2J*<>*cps2J*(G43R)-FLAG$\times$ NUS0650 | Str^R^, Zn^2+^ | This study |
| NUS1982 | *rpsL1 ∆cps2J<>cps2J* (S60L)-FLAG ∆*bgaA*::P_Zn_-*cps2J* | ∆*cps2J*<>*cps2J*(S60L)-FLAG$\times$ NUS0650 | Str^R^, Zn^2+^ | This study |
| NUS1983 | *rpsL1 ∆cps2J<>cps2J* (G342E)-FLAG ∆*bgaA*::P_Zn_-*cps2J* | ∆*cps2J*<>*cps2J*(G342E)-FLAG$\times$ NUS0650 | Str^R^, Zn^2+^ | This study |
| NUS1984 | *rpsL1 ∆cps2J<>cps2J* (G342R)-FLAG ∆*bgaA*::P_Zn_-*cps2J* | ∆*cps2J*<>*cps2J*(G342R)-FLAG$\times$ NUS0650 | Str^R^, Zn^2+^ | This study |
| NUS1985 | *rpsL1 ∆cps2J<>cps2J* (G346E)-FLAG ∆*bgaA*::P_Zn_-*cps2J* | ∆*cps2J*<>*cps2J*(G346E)-FLAG$\times$ NUS0650 | Str^R^, Zn^2+^ | This study |
| NUS1986 | *rpsL1 ∆cps2J<>cps10BJ*(T101I)-FLAG ∆*bgaA*::P_Zn_-*cps2J* | ∆*cps2J*<>*cps10BJ*(T101I)-FLAG$\times$ NUS0650 | Str^R^, Zn^2+^ | This study |
| NUS1987 | *rpsL1 ∆cps2J<>cps10BJ*(V109F)- FLAG ∆*bgaA*::P_Zn_-*cps2J* | ∆*cps2J*<>*cps10BJ*(V109F)-FLAG$\times$ NUS0650 | Str^R^, Zn^2+^ | This study |
| NUS1988 | *rpsL1 ∆cps2J<>cps10BJ*(N222E)- FLAG ∆*bgaA*::P_Zn_-*cps2J* | ∆*cps2J*<>*cps10BJ*(N222E)-FLAG$\times$ NUS0650 | Str^R^, Zn^2+^ | This study |
| NUS1989 | *rpsL1 ∆cps2J<>cps10BJ*(V356A)- FLAG ∆*bgaA*::P_Zn_-*cps2J* | ∆*cps2J*<>*cps10BJ*(V356A)-FLAG$\times$ NUS0650 | Str^R^, Zn^2+^ | This study |
| **Cps33BJ complementation** | | | | |
| NUS0552 | *rpsL1* CPS33B ∆*cps33BJ*<>*cps2J* ∆*bgaA*::P_Zn_-*cps33BJ* | ∆*cps33BJ*<>*cps2J* $\times$ NUS0490 | Str^R^, Zn^2+^ | This study |
| NUS1574 | *rpsL1* CPS33B ∆*cps33BJ*<>*cps10FJ* // ∆*bgaA*::P_Zn_-*cps33BJ* | ∆*cps33BJ*<>*cps10FJ* $\times$ NUS1549 | Str^R^, Zn^2+^ | This study |
| NUS1575 | *rpsL1* CPS33B ∆*cps33BJ*<>*cps10CJ* // ∆*bgaA*::P_Zn_-*cps33BJ* | ∆*cps33BJ*<>*cps10CJ* $\times$ NUS1549 | Str^R^, Zn^2+^ | This study |
| NUS1576 | *rpsL1* CPS33B ∆*cps33BJ*<>*cps13J* // ∆*bgaA*::P_Zn_-*cps33BJ* | ∆*cps33BJ*<>*cps13J* $\times$ NUS1549 | Str^R^, Zn^2+^ | This study |
| NUS1577 | *rpsL1* CPS33B ∆*cps33BJ*<>*cps33CJ* // ∆*bgaA*::P_Zn_-*cps33BJ* | ∆*cps33BJ*<>*cps33CJ* $\times$ NUS1549 | Str^R^, Zn^2+^ | This study |
| NUS1578 | *rpsL1* CPS33B ∆*cps33BJ*<>*cps34J* // ∆*bgaA*::P_Zn_-*cps33BJ* | ∆*cps33BJ*<>*cps34J* $\times$ NUS1549 | Str^R^, Zn^2+^ | This study |
| NUS1579 | *rpsL1* CPS33B ∆*cps33BJ*<>*cps35BJ* // ∆*bgaA*::P_Zn_-*cps33BJ* | ∆*cps33BJ*<>*cps35BJ* $\times$ NUS1549 | Str^R^, Zn^2+^ | This study |
| NUS1667 | *rpsL1* CPS33B ∆*cps33BJ*<>*cps8J* // ∆*bgaA*::P_Zn_-*cps33BJ* | ∆*cps33BJ*<>*cps8J* $\times$ NUS1549 | Str^R^, Zn^2+^ | This study |
| NUS1668 | *rpsL1* CPS33B ∆*cps33BJ*<>*cps9AJ* // ∆*bgaA*::P_Zn_-*cps33BJ* | ∆*cps33BJ*<>*cps9AJ* $\times$ NUS1549 | Str^R^, Zn^2+^ | This study |
| NUS1669 | *rpsL1* CPS33B ∆*cps33BJ*<>*cps12FJ* // ∆*bgaA*::P_Zn_-*cps33BJ* | ∆*cps33BJ*<>*cps12FJ* $\times$ NUS1549 | Str^R^, Zn^2+^ | This study |
| NUS1670 | *rpsL1* CPS33B ∆*cps33BJ*<>*cps14J* // ∆*bgaA*::P_Zn_-*cps33BJ* | ∆*cps33BJ*<>*cps14J* $\times$ NUS1549 | Str^R^, Zn^2+^ | This study |
| NUS1671 | *rpsL1* CPS33B ∆*cps33BJ*<>*cps23BJ* // ∆*bgaA*::P_Zn_-*cps33BJ* | ∆*cps33BJ*<>*cps23BJ* $\times$ NUS1549 | Str^R^, Zn^2+^ | This study |
| NUS1672 | *rpsL1* CPS33B ∆*cps33BJ*<>*cps40J* // ∆*bgaA*::P_Zn_-*cps33BJ* | ∆*cps33BJ*<>*cps40J* $\times$ NUS1549 | Str^R^, Zn^2+^ | This study |
| NUS1673 | *rpsL1* CPS33B ∆*cps33BJ*<>*cps42J* // ∆*bgaA*::P_Zn_-*cps33BJ* | ∆*cps33BJ*<>*cps42J* $\times$ NUS1549 | Str^R^, Zn^2+^ | This study |
| NUS1797 | *rpsL1* CPS33B ∆*cps33BJ*<>*cps10BJ* // ∆*bgaA*::P_Zn_-*cps33BJ* | ∆*cps33BJ*<>*cps10BJ* $\times$ NUS1549 | Str^R^, Zn^2+^ | This study |
| NUS1798 | *rpsL1* CPS33B ∆*cps33BJ*<>*cps19AJ* // ∆*bgaA*::P_Zn_-*cps33BJ* | ∆*cps33BJ*<>*cps19AJ* $\times$ NUS1549 | Str^R^, Zn^2+^ | This study |
| NUS1799 | *rpsL1* CPS33B ∆*cps33BJ*<>*cps28AJ* // ∆*bgaA*::P_Zn_-*cps33BJ* | ∆*cps33BJ*<>*cps28AJ* $\times$ NUS1549 | Str^R^, Zn^2+^ | This study |
| NUS1800 | *rpsL1* CPS33B ∆*cps33BJ*<>*cps29J* // ∆*bgaA*::P_Zn_-*cps33BJ* | ∆*cps33BJ*<>*cps29J* $\times$ NUS1549 | Str^R^, Zn^2+^ | This study |
| NUS1801 | *rpsL1* CPS33B ∆*cps33BJ*<>*cps36J* // ∆*bgaA*::P_Zn_-*cps33BJ* | ∆*cps33BJ*<>*cps36J* $\times$ NUS1549 | Str^R^, Zn^2+^ | This study |
| NUS1802 | *rpsL1* CPS33B ∆*cps33BJ*<>*cps45J* // ∆*bgaA*::P_Zn_-*cps33BJ* | ∆*cps33BJ*<>*cps45J* $\times$ NUS1549 | Str^R^, Zn^2+^ | This study |
| NUS1803 | *rpsL1* CPS33B ∆*cps33BJ*<>*cps47AJ* // ∆*bgaA*::P_Zn_-*cps33BJ* | ∆*cps33BJ*<>*cps47AJ* $\times$ NUS1549 | Str^R^, Zn^2+^ | This study |
| NUS1894 | *rpsL1* CPS33B ∆*cps33BJ*<>*cps33FJ* // ∆*bgaA*::P_Zn_-*cps33BJ* | ∆*cps33BJ*<>*cps33FJ* $\times$ NUS1549 | Str^R^, Zn^2+^ | This study |
| NUS1930 | *rpsL1* CPS33B ∆*cps33BJ*<>*cps18FJ* // ∆*bgaA*::P_Zn_-*cps33BJ* | ∆*cps33BJ*<>*cps18FJ* $\times$ NUS1549 | Str^R^, Zn^2+^ | This study |
| NUS1931 | *rpsL1* CPS33B ∆cps33BJ<>*cps21J* // ∆*bgaA*::P_Zn_-*cps33BJ* | ∆*cps33BJ*<>*cps21J* $\times$ NUS1549 | Str^R^, Zn^2+^ | This study |
| NUS1932 | *rpsL1* CPS33B ∆*cps33BJ*<>*cps39J* // ∆*bgaA*::P_Zn_-*cps33BJ* | ∆*cps33BJ*<>*cps39J* $\times$ NUS1549 | Str^R^, Zn^2+^ | This study |
| NUS1933 | *rpsL1* CPS33B ∆*cps33BJ*<>*cps47FJ* // ∆*bgaA*::P_Zn_-*cps33BJ* | ∆*cps33BJ*<>*cps47FJ* $\times$ NUS1549 | Str^R^, Zn^2+^ | This study |
| NUS1698 | *rpsL1* CPS33B ∆*bgaA*::p-*erm* | *∆bgaA::*P*-erm* $\times$ NUS0308 | Erm^R^, Str^R^ | This study |
| NUS1699 | *rpsL1* CPS33B ∆*cps33BJ*<>*cps10CJ* ∆*bgaA*::p-*erm* | *∆bgaA::*P*-erm* $\times$ NUS1575 | Erm^R^, Str^R^ | This study |
| NUS1700 | *rpsL1* CPS33B ∆*cps33BJ*<>*cps10FJ* ∆*bgaA*::p-*erm* | *∆bgaA::*P*-erm* $\times$ NUS1574 | Erm^R^, Str^R^ | This study |
| NUS1701 | *rpsL1* CPS33B ∆*cps33BJ*<>*cps13J* ∆*bgaA*::p-*erm* | *∆bgaA::*P*-erm* $\times$ NUS1576 | Erm^R^, Str^R^ | This study |
| NUS1702 | *rpsL1* CPS33B ∆*cps33BJ*<>*cps34J* ∆*bgaA*::p-*erm* | *∆bgaA::*P*-erm* $\times$ NUS1578 | Erm^R^, Str^R^ | This study |
| NUS1703 | rpsL1 CPS33B ∆*cps33BJ*<>*cps35BJ* ∆*bgaA*::p-*erm* | *∆bgaA::*P*-erm* $\times$ NUS1579 | Erm^R^, Str^R^ | This study |
| NUS1709 | *rpsL1* CPS33B ∆*cps33BJ*<>*cps8J* ∆*bgaA*::p-*erm* | *∆bgaA::*P*-erm* $\times$ NUS1667 | Erm^R^, Str^R^ | This study |
| NUS1710 | *rpsL1* CPS33B ∆*cps33BJ*<>*cps9AJ* ∆*bgaA*::p-*erm* | *∆bgaA::*P*-erm* $\times$ NUS1668 | Erm^R^, Str^R^ | This study |
| NUS1711 | *rpsL1* CPS33B ∆*cps33BJ*<>*cps12FJ* ∆*bgaA*::p-*erm* | *∆bgaA::*P*-erm* $\times$ NUS1669 | Erm^R^, Str^R^ | This study |
| NUS1712 | *rpsL1* CPS33B ∆*cps33BJ*<>*cps14J* ∆*bgaA*::p-*erm* | *∆bgaA::*P*-erm* $\times$ NUS1670 | Erm^R^, Str^R^ | This study |
| NUS1713 | *rpsL1* CPS33B ∆*cps33BJ*<>*cps23BJ* ∆*bgaA*::p-*erm* | *∆bgaA::*P*-erm* $\times$ NUS1671 | Erm^R^, Str^R^ | This study |
| NUS1714 | *rpsL1* CPS33B ∆*cps33BJ*<>*cps33CJ* ∆*bgaA*::p-*erm* | *∆bgaA::*P*-erm* $\times$ NUS1577 | Erm^R^, Str^R^ | This study |
| NUS1715 | *rpsL1* CPS33B ∆*cps33BJ*<>*cps40J* ∆*bgaA*::p-*erm* | *∆bgaA::*P*-erm* $\times$ NUS1672 | Erm^R^, Str^R^ | This study |
| NUS1716 | *rpsL1* CPS33B ∆*cps33BJ*<>*cps42J* ∆*bgaA*::p-*erm* | *∆bgaA::*P*-erm* $\times$ NUS1673 | Erm^R^, Str^R^ | This study |
| NUS1843 | *rpsL1* CPS33B ∆*cps33BJ*<>*cps10BJ* ∆*bgaA*::p-*erm* | *∆bgaA::*P*-erm* $\times$ NUS1797 | Erm^R^, Str^R^ | This study |
| NUS1844 | *rpsL1* CPS33B ∆*cps33BJ*<>*cps29J* ∆*bgaA*::p-*erm* | *∆bgaA::*P*-erm* $\times$ NUS1800 | Erm^R^, Str^R^ | This study |
| NUS1845 | *rpsL1* CPS33B ∆*cps33BJ*<>*cps36J* ∆*bgaA*::p-*erm* | *∆bgaA::*P*-erm* $\times$ NUS1673 | Erm^R^, Str^R^ | This study |
| NUS1846 | *rpsL1* CPS33B ∆*cps33BJ*<>*cps10BJ* ∆*bgaA*::p-*erm* | *∆bgaA::*P*-erm* $\times$ NUS1673 | Erm^R^, Str^R^ | This study |
| NUS1948 | *rpsL1* CPS33B ∆*cps33BJ*<>*cps21J* ∆*bgaA*::p-*erm* | *∆bgaA::*P*-erm* $\times$ NUS1931 | Erm^R^, Str^R^ | This study |
| NUS1949 | *rpsL1* CPS33B ∆*cps33BJ*<>*cps33FJ* ∆*bgaA*::p-*erm* | *∆bgaA::*P*-erm* $\times$ NUS1894 | Erm^R^, Str^R^ | This study |
| NUS1950 | *rpsL1* CPS33B ∆*cps33BJ*<>*cps39J* ∆*bgaA*::p-*erm* | *∆bgaA::*P*-erm* $\times$ NUS1932 | Erm^R^, Str^R^ | This study |
| NUS1951 | *rpsL1* CPS33B ∆*cps33BJ*<>*cps47FJ* ∆*bgaA*::p-*erm* | *∆bgaA::*P*-erm* $\times$ NUS1933 | Erm^R^, Str^R^ | This study |
| **Cps23BJ variants** | | | | |
| NUS1161 | *rpsL1* ∆*cps2E* ∆*cps2J*<>*cps23BJ* (A152T) ∆*bgaA*::P_Zn_-*cps2E* | ∆*cps2J*<>*cps23BJ* (A152T) $\times$ NUS0893 | Str^R^ | This study |
| NUS1162 | *rpsL1* ∆*cps2E* ∆*cps2J*<>*cps23BJ* (P254S) ∆*bgaA*::P_Zn_-*cps2E* | ∆*cps2J*<>*cps23BJ* (P254S) $\times$ NUS0893 | Str^R^ | This study |
| NUS1163 | *rpsL1* ∆*cps2E* ∆*cps2J*<>*cps23BJ* (F319L) ∆*bgaA*::P_Zn_-*cps2E* | ∆*cps2J*<>*cps23BJ* (F319L) $\times$ NUS0893 | Str^R^ | This study |
| NUS1183 | *rpsL1* ∆*cps2E* ∆*cps2J*<>*cps23BJ* (I31T) ∆*bgaA*::P_Zn_-*cps2E* | ∆*cps2J*<>*cps23BJ* (I31T) $\times$ NUS0893 | Str^R^ | This study |
| NUS1184 | *rpsL1* ∆*cps2E* ∆*cps2J*<>*cps23BJ* (T33A) ∆*bgaA*::P_Zn_-*cps2E* | ∆*cps2J*<>*cps23BJ* (T33A) $\times$ NUS0893 | Str^R^ | This study |
| NUS1185 | *rpsL1* ∆*cps2E* ∆*cps2J*<>*cps23BJ* (P30S) ∆*bgaA*::P_Zn_-*cps2E* | ∆*cps2J*<>*cps23BJ* (P30S) $\times$ NUS0893 | Str^R^ | This study |
| NUS1186 | *rpsL1* ∆*cps2E* ∆*cps2J*<>*cps23BJ* (L156P) ∆*bgaA*::P_Zn_-*cps2E* | ∆*cps2J*<>*cps23BJ* (L156P) $\times$ NUS0893 | Str^R^ | This study |
| NUS1187 | *rpsL1* ∆*cps2E* ∆*cps2J*<>*cps23BJ* (D231G) ∆*bgaA*::P_Zn_-*cps2E* | ∆*cps2J*<>*cps23BJ* (D231G)$\times$ NUS0893 | Str^R^ | This study |
| NUS1199 | *rpsL1* ∆*cps2E* ∆*cps2J*<>*cps23BJ* (WT) ∆*bgaA*::P_Zn_-*cps2E* | ∆*cps2J*<>*cps23BJ* (WT)$\times$ NUS0893 | Str^R^ | This study |
| NUS1337 | *rpsL1* ∆*cps2E* ∆*cps2J*<>*cps23BJ* (P30L) ∆*bgaA*::P_Zn_-*cps2E* | ∆*cps2J*<>*cps23BJ* (P30L)$\times$ NUS0893 | Str^R^ | This study |
| NUS1338 | *rpsL1* ∆*cps2E* ∆*cps2J*<>*cps23BJ* (Y41H) ∆*bgaA*::P_Zn_-*cps2E* | ∆*cps2J*<>*cps23BJ* (Y41H)$\times$ NUS0893 | Str^R^ | This study |
| NUS1339 | *rpsL1* ∆*cps2E* ∆*cps2J*<>*cps23BJ* (I241T) ∆*bgaA*::P_Zn_-*cps2E* | ∆*cps2J*<>*cps23BJ* (I241T)$\times$ NUS0893 | Str^R^ | This study |
| NUS1340 | *rpsL1* ∆*cps2E* ∆*cps2J*<>*cps23BJ* (F315L) ∆*bgaA*::P_Zn_-*cps2E* | ∆*cps2J*<>*cps23BJ* (F315L)$\times$ NUS0893 | Str^R^ | This study |
| NUS1341 | *rpsL1* ∆*cps2E* ∆*cps2J*<>*cps23BJ* (G316E) ∆*bgaA*::P_Zn_-*cps2E* | ∆*cps2J*<>*cps23BJ* (G316E)$\times$ NUS0893 | Str^R^ | This study |
| NUS1342 | *rpsL1* ∆*cps2E* ∆*cps2J*<>*cps23BJ* (F319S) ∆*bgaA*::P_Zn_-*cps2E* | ∆*cps2J*<>*cps23BJ* (F319S)$\times$ NUS0893 | Str^R^ | This study |
| NUS1347 | *rpsL1* ∆*cps2E* ∆*cps2J*<>*cps23BJ* (V29A) ∆*bgaA*::P_Zn_-*cps2E* | ∆*cps2J*<>*cps23BJ* (V29A)$\times$ NUS0893 | Str^R^ | This study |
| NUS1348 | *rpsL1* ∆*cps2E* ∆*cps2J*<>*cps23BJ* (A250V) ∆*bgaA*::P_Zn_-*cps2E* | ∆*cps2J*<>*cps23BJ* (A250V)$\times$ NUS0893 | Str^R^ | This study |
| NUS1349 | *rpsL1* ∆*cps2E* ∆*cps2J*<>*cps23BJ* (S244G) ∆*bgaA*::P_Zn_-*cps2E* | ∆*cps2J*<>*cps23BJ* (S244G)$\times$ NUS0893 | Str^R^ | This study |
| NUS1202 | *rpsL1* ∆*cps2J*<>*cps23BJ*(I31T) ∆*bgaA*::P_Zn_-*cps2J* | ∆*cps2J*<>*cps23BJ*(I31T)$\times$ NUS0650 | Str^R^, Zn^2+^ | This study |
| NUS1203 | *rpsL1* ∆*cps2J*<>*cps23BJ*(P30S) ∆*bgaA*::P_Zn_-*cps2J* | ∆*cps2J*<>*cps23BJ*(P30S)$\times$ NUS0650 | Str^R^, Zn^2+^ | This study |
| NUS1204 | *rpsL1* ∆*cps2J*<>*cps23BJ*(T33A) ∆*bgaA*::P_Zn_-*cps2J* | ∆*cps2J*<>*cps23BJ*(T33A)$\times$ NUS0650 | Str^R^, Zn^2+^ | This study |
| NUS1205 | *rpsL1* ∆*cps2J*<>*cps23BJ*(A152T) ∆*bgaA*::P_Zn_-*cps2J* | ∆*cps2J*<>*cps23BJ*(A152T)$\times$ NUS0650 | Str^R^, Zn^2+^ | This study |
| NUS1206 | *rpsL1* ∆*cps2J*<>*cps23BJ*(P254S) ∆*bgaA*::P_Zn_-*cps2J* | ∆*cps2J*<>*cps23BJ*(P254S)$\times$ NUS0650 | Str^R^, Zn^2+^ | This study |
| NUS1207 | *rpsL1* ∆*cps2J*<>*cps23BJ*(L156P) ∆*bgaA*::P_Zn_-*cps2J* | ∆*cps2J*<>*cps23BJ*(L156P)$\times$ NUS0650 | Str^R^, Zn^2+^ | This study |
| NUS1208 | *rpsL1* ∆*cps2J*<>*cps23BJ*(D231G) ∆*bgaA*::P_Zn_-*cps2J* | ∆*cps2J*<>*cps23BJ*(D231G)$\times$ NUS0650 | Str^R^, Zn^2+^ | This study |
| NUS1209 | *rpsL1* ∆*cps2J*<>*cps23BJ*(F319L) ∆*bgaA*::P_Zn_-*cps2J* | ∆*cps2J*<>*cps23BJ*(F319L)$\times$ NUS0650 | Str^R^, Zn^2+^ | This study |
| NUS1298 | *rpsL1* ∆*cps2J*<>*cps23BJ*(P30L) ∆*bgaA*::P_Zn_-*cps2J* | ∆*cps2J*<>*cps23BJ*(P30L)$\times$ NUS0650 | Str^R^, Zn^2+^ | This study |
| NUS1469 | *rpsL1* ∆*cps2J*<>*cps23BJ*(V29A) ∆*bgaA*::P_Zn_-*cps2J* | ∆*cps2J*<>*cps23BJ*(V29A)$\times$ NUS0650 | Str^R^, Zn^2+^ | This study |
| NUS1470 | *rpsL1* ∆*cps2J*<>*cps23BJ*(Y41H) ∆*bgaA*::P_Zn_-*cps2J* | ∆*cps2J*<>*cps23BJ*(Y41H)$\times$ NUS0650 | Str^R^, Zn^2+^ | This study |
| NUS1471 | *rpsL1* ∆*cps2J*<>*cps23BJ*(I241T) ∆*bgaA*::P_Zn_-*cps2J* | ∆*cps2J*<>*cps23BJ*(I241T)$\times$ NUS0650 | Str^R^, Zn^2+^ | This study |
| NUS1472 | *rpsL1* ∆*cps2J*<>*cps23BJ*(F319S) ∆*bgaA*::P_Zn_-*cps2J* | ∆*cps2J*<>*cps23BJ*(F319S)$\times$ NUS0650 | Str^R^, Zn^2+^ | This study |
| NUS1473 | *rpsL1* ∆*cps2J*<>*cps23BJ*(F315L) ∆*bgaA*::P_Zn_-*cps2J* | ∆*cps2J*<>*cps23BJ*(F315L)$\times$ NUS0650 | Str^R^, Zn^2+^ | This study |
| NUS1474 | *rpsL1* ∆*cps2J*<>*cps23BJ*(G316E) ∆*bgaA*::P_Zn_-*cps2J* | ∆*cps2J*<>*cps23BJ*(G316E)$\times$ NUS0650 | Str^R^, Zn^2+^ | This study |
| NUS1475 | *rpsL1* ∆*cps2J*<>*cps23BJ*(A250V) ∆*bgaA*::P_Zn_-*cps2J* | ∆*cps2J*<>*cps23BJ*(A250V)$\times$ NUS0650 | Str^R^, Zn^2+^ | This study |
| NUS1476 | *rpsL1* ∆*cps2J*<>*cps23BJ*(S244G) ∆*bgaA*::P_Zn_-*cps2J* | ∆*cps2J*<>*cps23BJ*(S244G)$\times$ NUS0650 | Str^R^, Zn^2+^ | This study |

^a^ Strains were constructed by transformation of amplicons (left of “x”) into the indicated recipient strain (right of “x”) as described in *Experimental procedures*. <> or :: indicates exact replacement of a reading frame or insertion, respectively. Primers for constructing the strains are listed in Table S2.

^b^ “P” refers to the constitutive synthetic promoter that drives the erythromycin resistant gene and the Janus cassette (1) and “P_Zn_” refers to the *czcD* operon promoter (2).

^c^ In *bgaA*, ∆*cps2E*, ∆*cps2J*, and ∆*cps33BJ* mutations, 60 bp of the open reading frame was left intact on the 5’ and 3’ junctions surrounding the inserted cassettes to maintain adjacent genetic signals. In ∆*cps* mutation, the deletion was from the first base of the *cpsA* to the last 60 bp of the *rfbD* reading frame.

^d^ Selectable markers and medium supplements: Erm , erythromycin; Kan , kanamycin; Str , streptomycin; Suc, sucrose. Zn^2+^: ZnCl_2_/MnCl_2_ (see *Experimental procedures*).

**REFERENCE**

1. Kazmierczak, K. M., Wayne, K. J., Rechtsteiner, A. & Winkler, M. E. Roles of *rel* *_Spn_* in stringent response, global regulation and virulence of serotype 2 *Streptococcus pneumoniae* D39. *Molecular Microbiology* **72**, 590–611 (2009).

2. Fenton, A. K., El Mortaji, L., Lau, D. T. C., Rudner, D. Z. & Bernhardt, T. G. CozE is a member of the MreCD complex that directs cell elongation in *Streptococcus pneumoniae*. *Nat Microbiol* **2**, 16237 (2017).

3. Lanie, J. A. *et al.* Genome Sequence of Avery’s Virulent Serotype 2 Strain D39 of *Streptococcus pneumoniae* and Comparison with That of Unencapsulated Laboratory Strain R6. *JB* **189**, 38–51 (2007).

4. Li, Y., Thompson, C. M. & Lipsitch, M. A Modified Janus Cassette (Sweet Janus) to Improve Allelic Replacement Efficiency by High-Stringency Negative Selection in *Streptococcus pneumoniae*. *PLoS ONE* **9**, e100510 (2014).

**Table S1B.** Oligonucleotides used in this study

| Primer | Sequence (5' to 3') | Template | Amplicon |
| --- | --- | --- | --- |
| For construction of ∆*cps2E*::P-*kan-rpsL^+^* | | | |
| CS207 | CTTGTGGAAACGCAAGCTAATG | D39 | *cps2E* 5’ |
| CS205 | CATTATCCATTAAAAATCAAACGGATCCTATAAAATAATAAGAAAACTCTGGATTATGGC |  |  |
| P1 | TAGGATCCGTTTGATTTTTAATGGATAATG | P-*kan*-*rpsL*^+^ cassette^[[2]](#endnote-2)^ | P-*kan-rpsL^+^* |
| P2 | GGGCCCCTTTCCTTATGCTTTTG |  |  |
| CS206 | CAAAAGCATAAGGAAAGGGGCCCGACATTGAAATTTTATTGAAGACAGTTAAAGTAG | D39 | *cps2E* 3’ |
| CS208 | AAGCCTAGTGGATGCCAATG |  |  |
| For construction of ∆*cps2E*::P-*sacB*-*kan*-*rpsL^+^* | | | |
| P64 | ACCAGCTACGACTCCTTCTTCT | HMS0001 | *cps2E* 5’ |
| P251 | CATTATCCATTAAAAATCAAACGGATCCTATAAAATAATAAGAAAACTCTGGATTATGG |  |  |
| P249 | TAGGATCCGTTTGATTTTTAATGGATAATGTTAAGGATCGATCCGTTTGATTTTTAATGG | SpnYL001 | P-*sacB*-*kan*-*rpsL^+^* |
| P250 | GGGCCCCTTTCCTTATGCTTTTGGACGTTTAGTACCGTATTTAGAACGG |  |  |
| P252 | CAAAAGCATAAGGAAAGGGGCCCGACATTGAAATTTTATTGAAGACAGTTAAAG | HMS0001 | *cps2E* 3’ |
| P65 | TCGTCCCACCACTAGATAATAGCC |  |  |
| For construction of ∆*cpsE* | | | |
| CS207 | CTTGTGGAAACGCAAGCTAATG | D39 | *cps2E* 5’ |
| CS210 | CTTTAACTGTCTTCAATAAAATTTCAATGTCTAAAATAATAAGAAAACTCTGGATTATGG |  |  |
| CS209 | CCATAATCCAGAGTTTTCTTATTATTTTAGACATTGAAATTTTATTGAAGACAGTTAAAG | D39 | *cps2E* 3’ |
| CS208 | AAGCCTAGTGGATGCCAATG |  |  |
| For construction of ∆*bgaA*::P-*erm* | | | |
| P23 | CCGTAGAACCACTATCACAAG | D39 | *bgaA* 5’ |
| P24 | TTATCCATTAAAAATCAAACGGATCCTATCCCACAGCAAACTTACGAATGCTATAAACTC |  |  |
| P1 | TAGGATCCGTTTGATTTTTAATGGATAATG | P-*erm* cassettea | P-*erm* |
| P2 | GGGCCCCTTTCCTTATGCTTTTG |  |  |
| P25 | AAAAGCATAAGGAAAGGGGCCCTTAGCTCTTCTAGGTTTGAGTGCAGGATTAGTAGTTAC | D39 | *bgaA* 3’ |
| P6 | TGCATGGTTACGATAGTCTTGG |  |  |
| For construction of ∆*bgaA*::P-*kan-rpsL^+^* | | | |
| P23 | CCGTAGAACCACTATCACAAG | D39 | *bgaA* 5’ |
| P24 | TTATCCATTAAAAATCAAACGGATCCTATCCCACAGCAAACTTACGAATGCTATAAACTC |  |  |
| P1 | TAGGATCCGTTTGATTTTTAATGGATAATG | HMS0001 | P-*kan-rpsL^+^* |
| P2 | GGGCCCCTTTCCTTATGCTTTTG |  |  |
| P25 | AAAAGCATAAGGAAAGGGGCCCTTAGCTCTTCTAGGTTTGAGTGCAGGATTAGTAGTTAC | D39 | *bgaA* 3’ |
| P26 | GACGAAACTTTGCGGATTTG |  |  |
| For construction of ∆*bgaA*::P_Zn_-*cps2J* | | | |
| P86 | GTTTGACTGCCGGTGTATCT | AKF_Spn024 | *bgaA*-Pzn 5’ |
| P147 | ATTTCTCATTCCTTTGTTATAATAG |  |  |
| P148 | CTATTATAACAAAGGAATGAGAAATTTGAGTAGAAGATATAATTACTTAC | D39 | *cps2J* |
| P41 | TTATGTTAGAAACTTTTTTAATTCA |  |  |
| P180 | GAATTAAAAAAGTTTCTAACATAATTAGCTCTTCTAGGTTTGAGTGCAGG | D39 | *bgaA* 3’ |
| P6 | TGCATGGTTACGATAGTCTTGG |  |  |
| For construction of ∆*cps2J*::P-*erm* | | | |
| P51 | GAACATGGAAATGTGGAAGATGAG | D39 | *cps2J* 5’ |
| P52 | CATTATCCATTAAAAATCAAACGGATCCTAAAAGTTGCTTAAAGTTAGTAATCC |  |  |
| P1 | TAGGATCCGTTTGATTTTTAATGGATAATG | P-*erm* cassette^a^ | P-*erm* |
| P2 | GGGCCCCTTTCCTTATGCTTTTG |  |  |
| P53 | CAAAAGCATAAGGAAAGGGGCCCATGTTATTTTATAAAGAAATAAAGAGTATTATTGG | D39 | *cps2J* 3’ |
| P54 | CATAGCCGAAGGAAGGATTGT |  |  |
| For construction of ∆*cps2J*::P-*kan*-*rpsL^+^* | | | |
| P51 | GAACATGGAAATGTGGAAGATGAG | D39 | *cps2J* 5’ |
| P52 | CATTATCCATTAAAAATCAAACGGATCCTAAAAGTTGCTTAAAGTTAGTAATCC |  |  |
| P1 | TAGGATCCGTTTGATTTTTAATGGATAATG | HMS0001 | P-*kan-rpsL^+^* |
| P2 | GGGCCCCTTTCCTTATGCTTTTG |  |  |
| P53 | CAAAAGCATAAGGAAAGGGGCCCATGTTATTTTATAAAGAAATAAAGAGTATTATTGG | D39 | *cps2J* 3’ |
| P54 | CATAGCCGAAGGAAGGATTGT |  |  |
| For construction of ∆*cps2J* | | | |
| P51 | GAACATGGAAATGTGGAAGATGAG | D39 | *cps2I-cps2J* 5’ |
| P268 | CTCTTTATTTCTTTATAAAATAACATAAAGTTGCTTAAAGTTAGTAATCC |  |  |
| P269 | GGATTACTAACTTTAAGCAACTTTATGTTATTTTATAAAGAAATAAAGAG | D39 | *cps2J-cps2K* 3’ |
| P54 | CATAGCCGAAGGAAGGATTGT |  |  |
| For construction of ∆*cps2J*::P-*sacB*-*kan*-*rpsL^+^* | | | |
| P51 | GAACATGGAAATGTGGAAGATGAG | D39 | *cps2J* 5’ |
| P52 | CATTATCCATTAAAAATCAAACGGATCCTAAAAGTTGCTTAAAGTTAGTAATCC |  |  |
| P249 | TAGGATCCGTTTGATTTTTAATGGATAATGTTAAGGATCGATCCGTTTGATTTTTAATGG | SpnYL001 | P-*sacB*-*kan*-*rpsL^+^* |
| P250 | GGGCCCCTTTCCTTATGCTTTTGGACGTTTAGTACCGTATTTAGAACGG |  |  |
| P53 | CAAAAGCATAAGGAAAGGGGCCCATGTTATTTTATAAAGAAATAAAGAGTATTATTGG | D39 | *cps2J* 3’ |
| P54 | CATAGCCGAAGGAAGGATTGT |  |  |
| For construction of ∆*bgaA*::P-*sacB*-*kan*-*rpsL^+^* | | | |
| P64 | ACCAGCTACGACTCCTTCTTCT | HMS0001 | *bgaA* 5' |
| P251 | CATTATCCATTAAAAATCAAACGGATCCTATAAAATAATAAGAAAACTCTGGATTATGG |  |  |
| P249 | TAGGATCCGTTTGATTTTTAATGGATAATGTTAAGGATCGATCCGTTTGATTTTTAATGG | SpnYL001 | P-*sacB*-*kan*-*rpsL^+^* |
| P250 | GGGCCCCTTTCCTTATGCTTTTGGACGTTTAGTACCGTATTTAGAACGG |  |  |
| P252 | CAAAAGCATAAGGAAAGGGGCCCGACATTGAAATTTTATTGAAGACAGTTAAAG | HMS0001 | *bgaA* 3' |
| P65 | TCGTCCCACCACTAGATAATAGCC |  |  |
| For construction of ∆*bgaA*::P_Zn_-*cps2E* | | | |
| P86 | GTTTGACTGCCGGTGTATCT | AKF_Spn024 | *bgaA*::P_Zn_ 5’ |
| P413 | GCCAATGAAGACTTTACTGTTTTTCCATTCATATTTCTCATTCCTTTGTTATAATAG |  |  |
| P208 | ATGAATGGAAAAACAGTAAAGTCTTC | D39 | *cps2E* ORF |
| P209 | CTACTTCGCTCCATCTCTCATAAATAC |  |  |
| P421 | GTAGTATTTATGAGAGATGGAGCGAAGTAGTTAGCTCTTCTAGGTTTGAGTGCAGG | D39 | *bgaA* 3’ |
| P6 | TGCATGGTTACGATAGTCTTGG |  |  |
| For construction of ∆*bgaA*::P*_SpxB_*-*cps19AJ* | | | |
| P23 | CCGTAGAACCACTATCACAAG | D39 | *bgaA*:: P*_SpxB_* 5’ |
| P1787 | AATGATAACTCTCCTTCAATTTTTTTAAAC |  |  |
| P2198 | TTGAAGGAGAGTTATCATTATGAATAGCAAAATTAAAAATATAC | NUH0013 | *cps19AJ* |
| P1273 | TTAATTTGATGTTTTTTTACTAGCT |  |  |
| P2199 | GCTAGTAAAAAAACATCAAATTAATTAGCTCTTCTAGGTTTGAG | D39 | *bgaA* 3’ |
| P6 | TGCATGGTTACGATAGTCTTGG |  |  |
| For construction of ∆*cps19AJ*::P-*erm* | | | |
| P2417 | ACGCTCTAAAGATTTACTGCTCTTA | NUS0013 | *cps19AJ* 5’ |
| P2418 | CATTAAAAATCAAACGGATCCTAAAGATTTGAAGAAATGACATAA |  |  |
| P1 | TAGGATCCGTTTGATTTTTAATGGATAATG | P-*erm* cassette^a^ | P-*erm* |
| P2 | GGGCCCCTTTCCTTATGCTTTTG |  |  |
| P2419 | CAAAAGCATAAGGAAAGGGGCCCCACGAAGATATCAAAACCTAT | NUS0013 | *cps19AJ* 3’ |
| P2420 | AACTTTGGTTCATGAAGTTGTGG |  |  |
| For construction of *∆cps2J<>cps15AJ* | | | |
| P51 | GAACATGGAAATGTGGAAGATGAG | D39 | *cps2J* 5’ |
| P235 | AATTTTTACTAATTTTATTACTCATTTTTCTAGTTCCTTATATAGTTGCATGATCTGC |  |  |
| P236 | ATGAGTAATAAAATTAGTAAAAATT | NUH0008 | *cps15AJ* |
| P237 | TTATAATGTATTTTTATGTACAACC |  |  |
| P238 | GGTTGTACATAAAAATACATTATAAGAACCAATAAGTACGAGTATTGAAAGG | D39 | *cps2J* 3’ |
| P54 | CATAGCCGAAGGAAGGATTGT |  |  |
| For construction of *∆cps2J<>cps23FJ* | | | |
| P51 | GAACATGGAAATGTGGAAGATGAG | D39 | *cps2J* 5’ |
| P160 | TTGCTAATTCCTTATATTTACTCATTTTTCTAGTTCCTTATATAGTTGCATGATCTGC |  |  |
| P161 | ATGAGTAAATATAAGGAATTAGCAA | NUH0017 | *cps23FJ* |
| P162 | CTAATTTCTTTTGAAAATTTTTAAA |  |  |
| P163 | TTTAAAAATTTTCAAAAGAAATTAGGAACCAATAAGTACGAGTATTGAAAGG | D39 | *cps2J* 3’ |
| P54 | CATAGCCGAAGGAAGGATTGT |  |  |
| For construction of *∆cps2J<>cps35BJ* | | | |
| P51 | GAACATGGAAATGTGGAAGATGAG | D39 | *cps2J* 5’ |
| P168 | ATGCATAGTTTTTAAGTACTTTCATTTTTCTAGTTCCTTATATAGTTGCATGATCTGC |  |  |
| P169 | ATGAAAGTACTTAAAAACTATGCAT | PATH51 | *cps35BJ* |
| P170 | CTAATTTTTCCTAATTACTTGTTTT |  |  |
| P171 | AAAACAAGTAATTAGGAAAAATTAGGAACCAATAAGTACGAGTATTGAAAGG | D39 | *cps2J* 3’ |
| P54 | CATAGCCGAAGGAAGGATTGT |  |  |
| For construction of *∆cps2J<>cps8J* | | | |
| P51 | GAACATGGAAATGTGGAAGATGAG | D39 | *cps2J* 5’ |
| P227 | TTCTAGTAATAGATTTTTTAGTCATTTTTCTAGTTCCTTATATAGTTGCATGATCTGC |  |  |
| P228 | ATGACTAAAAAATCTATTACTAGAA | NUH0006 | *cps8J* |
| P229 | TCATTCTTTAATAATATTAAACCGA |  |  |
| P230 | TCGGTTTAATATTATTAAAGAATGAGAACCAATAAGTACGAGTATTGAAAGG | D39 | *cps2J* 3’ |
| P54 | CATAGCCGAAGGAAGGATTGT |  |  |
| For construction of *∆cps2J<>cps34J* | | | |
| P51 | GAACATGGAAATGTGGAAGATGAG | D39 | *cps2J* 5’ |
| P164 | AGGCGTAGTTTTTTAGTACTTTCATTTTTCTAGTTCCTTATATAGTTGCATGATCTGC |  |  |
| P165 | ATGAAAGTACTAAAAAACTACGCCT | CCUG2399 | *cps34J* |
| P166 | TTATTTTTTAATAATATGTTTTAAT |  |  |
| P167 | ATTAAAACATATTATTAAAAAATAAGAACCAATAAGTACGAGTATTGAAAGG | D39 | *cps2J* 3’ |
| P54 | CATAGCCGAAGGAAGGATTGT |  |  |
| For construction of *∆cps2J<>cps14J* | | | |
| P51 | GAACATGGAAATGTGGAAGATGAG | D39 | *cps2J* 5’ |
| P231 | AATTTTTACTGATTTTATTACTCATTTTTCTAGTTCCTTATATAGTTGCATGATCTGC |  |  |
| P232 | ATGAGTAATAAAATCAGTAAAAATT | NUH0006 | *cps14J* |
| P233 | TTATAATATATTTTCATAACCAATC |  |  |
| P234 | GATTGGTTATGAAAATATATTATAAGAACCAATAAGTACGAGTATTGAAAGG | D39 | *cps2J* 3’ |
| P54 | CATAGCCGAAGGAAGGATTGT |  |  |
| For construction of *∆cps2J<>cps6AJ* | | | |
| P51 | GAACATGGAAATGTGGAAGATGAG | D39 | *cps2J* 5’ |
| P223 | TTGTTATAAGAAACTTCAATTTCATTTTTCTAGTTCCTTATATAGTTGCATGATCTGC |  |  |
| P224 | ATGAAATTGAAGTTTCTTATAACAA | NUH0002 | *cps6AJ* |
| P225 | TTATTCAAATATTTTCTTTCTAAAA |  |  |
| P226 | TTTTAGAAAGAAAATATTTGAATAAGAACCAATAAGTACGAGTATTGAAAGG | D39 | *cps2J* 3’ |
| P54 | CATAGCCGAAGGAAGGATTGT |  |  |
| For construction of *∆cps2J<>cps23BJ* | | | |
| P51 | GAACATGGAAATGTGGAAGATGAG | D39 | *cps2J* 5’ |
| P160 | TTGCTAATTCCTTATATTTACTCATTTTTCTAGTTCCTTATATAGTTGCATGATCTGC |  |  |
| P161 | ATGAGTAAATATAAGGAATTAGCAA | PATH212 | *cps*23BJ |
| P361 | TCATTTAAATCCTTTTAATAATTTT |  |  |
| P362 | AAAATTATTAAAAGGATTTAAATGAGAACCAATAAGTACGAGTATTGAAAGG | D39 | *cps2J* 3’ |
| P54 | CATAGCCGAAGGAAGGATTGT |  |  |
| For construction of *∆cps2J<>cps33CJ* | | | |
| P51 | GAACATGGAAATGTGGAAGATGAG | D39 | *cps2J* 5’ |
| P367 | AAGCGTAGTTTTTTAGTACTTTCATTTTTCTAGTTCCTTATATAGTTGCATGATCTGC |  |  |
| P368 | ATGAAAGTACTAAAAAACTACGCTT | PATH344 | *cps*33CJ |
| P369 | TTATTTCTTAATGATTTGTTTTAAT |  |  |
| P370 | ATTAAAACAAATCATTAAGAAATAAGAACCAATAAGTACGAGTATTGAAAGG | D39 | *cps2J* 3’ |
| P54 | CATAGCCGAAGGAAGGATTGT |  |  |
| For construction of *∆cps2J<>cps35AJ* | | | |
| P51 | GAACATGGAAATGTGGAAGATGAG | D39 | *cps2J* 5’ |
| P371 | ATAAGTAGTTTTTTAATACCTTCATTTTTCTAGTTCCTTATATAGTTGCATGATCTGC |  |  |
| P372 | ATGAAGGTATTAAAAAACTACTTAT | PATH1709 | *cps*35AJ |
| P373 | CTAGTTTTTCCTAATAATTTGTTTT |  |  |
| P374 | AAAACAAATTATTAGGAAAAACTAGGAACCAATAAGTACGAGTATTGAAAGG | D39 | *cps2J* 3’ |
| P54 | CATAGCCGAAGGAAGGATTGT |  |  |
| For construction of *∆cps2J<>cps33BJ* | | | |
| P51 | GAACATGGAAATGTGGAAGATGAG | D39 | *cps2J* 5’ |
| P363 | AGGCGTAATTTTTTAGTACTTTCATTTTTCTAGTTCCTTATATAGTTGCATGATCTGC |  |  |
| P364 | ATGAAAGTACTAAAAAATTACGCCT | PATH1945 | *cps*33BJ |
| P365 | TTAGTTTTTCCTAATAATTTGTTTT |  |  |
| P366 | AAAACAAATTATTAGGAAAAACTAAGAACCAATAAGTACGAGTATTGAAAGG | D39 | *cps2J* 3’ |
| P54 | CATAGCCGAAGGAAGGATTGT |  |  |
| For construction of *∆cps2J<>cps33AJ* | | | |
| P51 | GAACATGGAAATGTGGAAGATGAG | D39 | *cps2J* 5’ |
| P640 | AGGCATAGTTTTTTAGTATTTTCATTTTTCTAGTTCCTTATATAGTTGCATGATCTGC |  |  |
| P641 | ATGAAAATACTAAAAAACTATGCCT | PATH1754 | *cps*33AJ |
| P642 | CTAATTTTTCCTGATTATTTGTTTT |  |  |
| P643 | AAAACAAATAATCAGGAAAAATTAGGAACCAATAAGTACGAGTATTGAAAGG | D39 | *cps2J* 3’ |
| P54 | CATAGCCGAAGGAAGGATTGT |  |  |
| For construction of *∆cps2J<>cps33DJ* | | | |
| P51 | GAACATGGAAATGTGGAAGATGAG | D39 | *cps2J* 5’ |
| P363 | AGGCGTAATTTTTTAGTACTTTCATTTTTCTAGTTCCTTATATAGTTGCATGATCTGC |  |  |
| P364 | ATGAAAGTACTAAAAAATTACGCCT | PATH2481 | *cps*33DJ |
| P365 | TTAGTTTTTCCTAATAATTTGTTTT |  |  |
| P366 | AAAACAAATTATTAGGAAAAACTAAGAACCAATAAGTACGAGTATTGAAAGG | D39 | *cps2J* 3’ |
| P54 | CATAGCCGAAGGAAGGATTGT |  |  |
| For construction of *∆cps2J<>cps33FJ* | | | |
| P51 | GAACATGGAAATGTGGAAGATGAG | D39 | *cps2J* 5’ |
| P640 | AGGCATAGTTTTTTAGTATTTTCATTTTTCTAGTTCCTTATATAGTTGCATGATCTGC |  |  |
| P641 | ATGAAAATACTAAAAAACTATGCCT | PATH101 | *cps*33FJ |
| P642 | CTAATTTTTCCTGATTATTTGTTTT |  |  |
| P643 | AAAACAAATAATCAGGAAAAATTAGGAACCAATAAGTACGAGTATTGAAAGG | D39 | *cps2J* 3’ |
| P54 | CATAGCCGAAGGAAGGATTGT |  |  |
| For construction of *∆cps2J<>cps10FJ* | | | |
| P51 | GAACATGGAAATGTGGAAGATGAG | D39 | *cps2J* 5’ |
| P239 | AGGCGTAGTTTTTTAATACTTTCATTTTTCTAGTTCCTTATATAGTTGCATGATCTGC |  |  |
| P240 | ATGAAAGTATTAAAAAACTACGCCT | PATH1539 | *cps*10FJ |
| P241 | CTATTTTTTCCTAATAATTTGTTTT |  |  |
| P242 | AAAACAAATTATTAGGAAAAAATAGGAACCAATAAGTACGAGTATTGAAAGG | D39 | *cps2J* 3’ |
| P54 | CATAGCCGAAGGAAGGATTGT |  |  |
| For construction of *∆cps2J<>cps10CJ* | | | |
| P51 | GAACATGGAAATGTGGAAGATGAG | D39 | *cps2J* 5’ |
| P239 | AGGCGTAGTTTTTTAATACTTTCATTTTTCTAGTTCCTTATATAGTTGCATGATCTGC |  |  |
| P240 | ATGAAAGTATTAAAAAACTACGCCT | PATH2460 | *cps*10CJ |
| P760 | TTATTTTTTCCTAATAATTTGTTTT |  |  |
| P761 | AAAACAAATTATTAGGAAAAAATAAGAACCAATAAGTACGAGTATTGAAAGGAG | D39 | *cps2J* 3’ |
| P54 | CATAGCCGAAGGAAGGATTGT |  |  |
| For construction of *∆cps2J<>cps13J* | | | |
| P51 | GAACATGGAAATGTGGAAGATGAG | D39 | *cps2J* 5’ |
| P756 | AGGCGTAGTTTTTTAAGATTTTCATTTTTCTAGTTCCTTATATAGTTGCATGATCTGC |  |  |
| P757 | ATGAAAATCTTAAAAAACTACGCCT | PATH1886 | *cps13J* |
| P758 | CTAATTTTTCCTAATAATTTGTTTT |  |  |
| P759 | AAAACAAATTATTAGGAAAAATTAGGAACCAATAAGTACGAGTATTGAAAGGAG | D39 | *cps2J* 3’ |
| P54 | CATAGCCGAAGGAAGGATTGT |  |  |
| For construction of *∆cps2J<>cps9AJ* | | | |
| P51 | GAACATGGAAATGTGGAAGATGAG | D39 | *cps2J* 5’ |
| P176 | CCTTAAACTTATTACTTATATCCATTTTTCTAGTTCCTTATATAGTTGCATGATCTGC |  |  |
| P177 | ATGGATATAAGTAATAAGTTTAAGG | PATH4969 | *cps9AJ* |
| P178 | TCACCTACTAAACATCATTATTAAC |  |  |
| P179 | GTTAATAATGATGTTTAGTAGGTGAGAACCAATAAGTACGAGTATTGAAAGG | D39 | *cps2J* 3’ |
| P54 | CATAGCCGAAGGAAGGATTGT |  |  |
| For construction of *∆cps2J<>cps5J* | | | |
| P51 | GAACATGGAAATGTGGAAGATGAG | D39 | *cps2J* 5’ |
| P857 | TAACTTGTTTCAGAATGTTCACCAATTTTCTAGTTCCTTATATAGTTGCATGATCTGC |  |  |
| P858 | TTGGTGAACATTCTGAAACAAGTTA | PATH46 | *cps5J* |
| P859 | TCATACGTTTTTCCTACTTTCTATA |  |  |
| P860 | TATAGAAAGTAGGAAAAACGTATGAGAACCAATAAGTACGAGTATTGAAAGG | D39 | *cps2J* 3’ |
| P54 | CATAGCCGAAGGAAGGATTGT |  |  |
| For construction of ∆*cps2J<>cps9LJ* | | | |
| P51 | GAACATGGAAATGTGGAAGATGAG | D39 | *cps2J* 5’ |
| P172 | GTTTCAAATTTTTTTCAACATTCATTTTTCTAGTTCCTTATATAGTTGCATGATCTGC |  |  |
| P173 | ATGAATGTTGAAAAAAATTTGAAAC | PATH2478 | *cps9LJ* |
| P174 | TTATTTTCTTATAACCGCGAAGACT |  |  |
| P175 | AGTCTTCGCGGTTATAAGAAAATAAGAACCAATAAGTACGAGTATTGAAAGG | D39 | *cps2J* 3’ |
| P54 | CATAGCCGAAGGAAGGATTGT |  |  |
| For construction of ∆*cps2J<>cps23AJ* | | | |
| P51 | GAACATGGAAATGTGGAAGATGAG | D39 | *cps2J* 5’ |
| P160 | TTGCTAATTCCTTATATTTACTCATTTTTCTAGTTCCTTATATAGTTGCATGATCTGC |  |  |
| P161 | ATGAGTAAATATAAGGAATTAGCAA | NUH0016 | *cps23AJ* |
| P162 | CTAATTTCTTTTGAAAATTTTTAAA |  |  |
| P163 | TTTAAAAATTTTCAAAAGAAATTAGGAACCAATAAGTACGAGTATTGAAAGG | D39 | *cps2J* 3’ |
| P54 | CATAGCCGAAGGAAGGATTGT |  |  |
| For construction of ∆*cps2J<>cps7BJ* | | | |
| P51 | GAACATGGAAATGTGGAAGATGAG | D39 | *cps2J* 5’ |
| P865 | TTTTATTTATTGATTTTTCTTTCATTTTTCTAGTTCCTTATATAGTTGCATGATCTGC |  |  |
| P866 | ATGAAAGAAAAATCAATAAATAAAA | PATH1803 | *cps7BJ* |
| P867 | TCATGAAATATCCTTATAAAAAATT |  |  |
| P868 | AATTTTTTATAAGGATATTTCATGAGAACCAATAAGTACGAGTATTGAAAGG | D39 | *cps2J* 3’ |
| P54 | CATAGCCGAAGGAAGGATTGT |  |  |
| For construction of ∆*cps2J<>cps1J* | | | |
| P51 | GAACATGGAAATGTGGAAGATGAG | D39 | *cps2J* 5’ |
| P849 | TATAAAAAATTTCTACTAAGTTCATTTTTCTAGTTCCTTATATAGTTGCATGATCTGC |  |  |
| P850 | ATGAACTTAGTAGAAATTTTTTATA | PATH106 | *cps1J* |
| P851 | TCAATTTCTAAGATAATCTATAATA |  |  |
| P852 | TATTATAGATTATCTTAGAAATTGAGAACCAATAAGTACGAGTATTGAAAGG | D39 | *cps2J* 3’ |
| P54 | CATAGCCGAAGGAAGGATTGT |  |  |
| For construction of ∆*cps2J<>cps7AJ* | | | |
| P51 | GAACATGGAAATGTGGAAGATGAG | D39 | *cps2J* 5’ |
| P861 | AAGCATAATTTTTTATTGTTTTCATTTTTCTAGTTCCTTATATAGTTGCATGATCTGC |  |  |
| P862 | ATGAAAACAATAAAAAATTATGCTT | PATH2477 | *cps7AJ* |
| P863 | TCAAAAAAATCTTGTGTTTTTATTT |  |  |
| P864 | AAATAAAAACACAAGATTTTTTTGAGAACCAATAAGTACGAGTATTGAAAGG | D39 | *cps2J* 3’ |
| P54 | CATAGCCGAAGGAAGGATTGT |  |  |
| For construction of ∆*cps2J<>cps7CJ* | | | |
| P51 | GAACATGGAAATGTGGAAGATGAG | D39 | *cps2J* 5’ |
| P869 | TTATTGATTTTTCTTTCATGCTCATTTTTCTAGTTCCTTATATAGTTGCATGATCTGC |  |  |
| P870 | ATGAGCATGAAAGAAAAATCAATAA | PATH203 | *cps7CJ* |
| P867 | TCATGAAATATCCTTATAAAAAATT |  |  |
| P868 | AATTTTTTATAAGGATATTTCATGAGAACCAATAAGTACGAGTATTGAAAGG | D39 | *cps2J* 3’ |
| P54 | CATAGCCGAAGGAAGGATTGT |  |  |
| For construction of ∆*cps2J<>cps7FJ* | | | |
| P51 | GAACATGGAAATGTGGAAGATGAG | D39 | *cps2J* 5’ |
| P871 | ATTGCAACTCCCCATGAAAGAACAATTTTCTAGTTCCTTATATAGTTGCATGATCTGC |  |  |
| P872 | TTGTTCTTTCATGGGGAGTTGCAAT | NUH0005 | *cps7FJ* |
| P863 | TCAAAAAAATCTTGTGTTTTTATTT |  |  |
| P864 | AAATAAAAACACAAGATTTTTTTGAGAACCAATAAGTACGAGTATTGAAAGG | D39 | *cps2J* 3’ |
| P54 | CATAGCCGAAGGAAGGATTGT |  |  |
| For construction of ∆*cps2J<>cps10AJ* | | | |
| P51 | GAACATGGAAATGTGGAAGATGAG | D39 | *cps2J* 5’ |
| P363 | AGGCGTAATTTTTTAGTACTTTCATTTTTCTAGTTCCTTATATAGTTGCATGATCTGC |  |  |
| P364 | ATGAAAGTACTAAAAAATTACGCCT | PATH691 | *cps10AJ* |
| P897 | TTATTTTTTTATGATTTGTTTTAAT |  |  |
| P898 | ATTAAAACAAATCATAAAAAAATAAGAACCAATAAGTACGAGTATTGAAAGG | D39 | *cps2J* 3’ |
| P54 | CATAGCCGAAGGAAGGATTGT |  |  |
| For construction of ∆*cps2J<>cps10BJ* | | | |
| P51 | GAACATGGAAATGTGGAAGATGAG | D39 | *cps2J* 5’ |
| P363 | AGGCGTAATTTTTTAGTACTTTCATTTTTCTAGTTCCTTATATAGTTGCATGATCTGC |  |  |
| P364 | ATGAAAGTACTAAAAAATTACGCCT | PATH2459 | *cps10BJ* |
| P897 | TTATTTTTTTATGATTTGTTTTAAT |  |  |
| P898 | ATTAAAACAAATCATAAAAAAATAAGAACCAATAAGTACGAGTATTGAAAGG | D39 | *cps2J* 3’ |
| P54 | CATAGCCGAAGGAAGGATTGT |  |  |
| For construction of ∆*cps2J<>cps39J* | | | |
| P51 | GAACATGGAAATGTGGAAGATGAG | D39 | *cps2J* 5’ |
| P909 | ACGCGTAATTTTTTAGTACTTTCATTTTTCTAGTTCCTTATATAGTTGCATGATCTGC |  |  |
| P910 | ATGAAAGTACTAAAAAATTACGCGT | PATH2009 | *cps39J* |
| P911 | TTAAGCTCCTTTATTTTTTAATAAT |  |  |
| P912 | ATTATTAAAAAATAAAGGAGCTTAAGAACCAATAAGTACGAGTATTGAAAGG | D39 | *cps2J* 3’ |
| P54 | CATAGCCGAAGGAAGGATTGT |  |  |
| For construction of ∆*cps2J<>cps15BJ* | | | |
| P51 | GAACATGGAAATGTGGAAGATGAG | D39 | *cps2J* 5’ |
| P235 | AATTTTTACTAATTTTATTACTCATTTTTCTAGTTCCTTATATAGTTGCATGATCTGC |  |  |
| P236 | ATGAGTAATAAAATTAGTAAAAATT | NUH0009 | *cps15BJ* |
| P237 | TTATAATGTATTTTTATGTACAACC |  |  |
| P238 | GGTTGTACATAAAAATACATTATAAGAACCAATAAGTACGAGTATTGAAAGG | D39 | *cps2J* 3’ |
| P54 | CATAGCCGAAGGAAGGATTGT |  |  |
| For construction of ∆*cps2J<>cps35CJ* | | | |
| P51 | GAACATGGAAATGTGGAAGATGAG | D39 | *cps2J* 5’ |
| P371 | ATAAGTAGTTTTTTAATACCTTCATTTTTCTAGTTCCTTATATAGTTGCATGATCTGC |  |  |
| P372 | ATGAAGGTATTAAAAAACTACTTAT | PATH1895 | *cps35CJ* |
| P373 | CTAGTTTTTCCTAATAATTTGTTTT |  |  |
| P374 | AAAACAAATTATTAGGAAAAACTAGGAACCAATAAGTACGAGTATTGAAAGG | D39 | *cps2J* 3’ |
| P54 | CATAGCCGAAGGAAGGATTGT |  |  |
| For construction of ∆*cps2J<>cps15FJ* | | | |
| P51 | GAACATGGAAATGTGGAAGATGAG | D39 | *cps2J* 5’ |
| P235 | AATTTTTACTAATTTTATTACTCATTTTTCTAGTTCCTTATATAGTTGCATGATCTGC |  |  |
| P236 | ATGAGTAATAAAATTAGTAAAAATT | NUH0011 | *cps15FJ* |
| P237 | TTATAATGTATTTTTATGTACAACC |  |  |
| P238 | GGTTGTACATAAAAATACATTATAAGAACCAATAAGTACGAGTATTGAAAGG | D39 | *cps2J* 3’ |
| P54 | CATAGCCGAAGGAAGGATTGT |  |  |
| For construction of ∆*cps2J<>cps15CJ* | | | |
| P51 | GAACATGGAAATGTGGAAGATGAG | D39 | *cps2J* 5’ |
| P235 | AATTTTTACTAATTTTATTACTCATTTTTCTAGTTCCTTATATAGTTGCATGATCTGC |  |  |
| P236 | ATGAGTAATAAAATTAGTAAAAATT | NUH0010 | *cps15CJ* |
| P237 | TTATAATGTATTTTTATGTACAACC |  |  |
| P238 | GGTTGTACATAAAAATACATTATAAGAACCAATAAGTACGAGTATTGAAAGG | D39 | *cps2J* 3’ |
| P54 | CATAGCCGAAGGAAGGATTGT |  |  |
| For construction of ∆*cps2J<>cps36J* | | | |
| P51 | GAACATGGAAATGTGGAAGATGAG | D39 | *cps2J* 5’ |
| P905 | ATGCATAATTTTTTAAAACCTTCATTTTTCTAGTTCCTTATATAGTTGCATGATCTGC |  |  |
| P906 | ATGAAGGTTTTAAAAAATTATGCAT | PATH1833 | *cps36J* |
| P907 | CTAATTATTTAACAGCTGCTCTTTT |  |  |
| P908 | AAAAGAGCAGCTGTTAAATAATTAGGAACCAATAAGTACGAGTATTGAAAGG | D39 | *cps2J* 3’ |
| P54 | CATAGCCGAAGGAAGGATTGT |  |  |
| For construction of ∆*cps2J<>cps31J* | | | |
| P51 | GAACATGGAAATGTGGAAGATGAG | D39 | *cps2J* 5’ |
| P899 | TTCGGGCAATGGATTTCCTTTTCACTTTTCTAGTTCCTTATATAGTTGCATGATCTGC |  |  |
| P900 | GTGAAAAGGAAATCCATTGCCCGAA | PATH18 | *cps31J* |
| P901 | CTATCTATATATTTTCTTTCTAACT |  |  |
| P902 | AGTTAGAAAGAAAATATATAGATAGGAACCAATAAGTACGAGTATTGAAAGG | D39 | *cps2J* 3’ |
| P54 | CATAGCCGAAGGAAGGATTGT |  |  |
| For construction of ∆*cps2J<>cps41AJ* | | | |
| P51 | GAACATGGAAATGTGGAAGATGAG | D39 | *cps2J* 5’ |
| P931 | CAATGGATTTCCTTTTCACAATCACTTTTCTAGTTCCTTATATAGTTGCATGATCTGC |  |  |
| P932 | GTGATTGTGAAAAGGAAATCCATTG | PATH2471 | *cps41AJ* |
| P901 | CTATCTATATATTTTCTTTCTAACT |  |  |
| P902 | AGTTAGAAAGAAAATATATAGATAGGAACCAATAAGTACGAGTATTGAAAGG | D39 | *cps2J* 3’ |
| P54 | CATAGCCGAAGGAAGGATTGT |  |  |
| For construction of ∆*cps2J<>cps41FJ* | | | |
| P51 | GAACATGGAAATGTGGAAGATGAG | D39 | *cps2J* 5’ |
| P899 | TTCGGGCAATGGATTTCCTTTTCACTTTTCTAGTTCCTTATATAGTTGCATGATCTGC |  |  |
| P900 | GTGAAAAGGAAATCCATTGCCCGAA | PATH2470 | *cps41FJ* |
| P933 | TTATCTATATATTTTCTTTCTGACT |  |  |
| P934 | AGTCAGAAAGAAAATATATAGATAAGAACCAATAAGTACGAGTATTGAAAGG | D39 | *cps2J* 3’ |
| P54 | CATAGCCGAAGGAAGGATTGT |  |  |
| For construction of ∆*cps2J<>cps43J* | | | |
| P51 | GAACATGGAAATGTGGAAGATGAG | D39 | *cps2J* 5’ |
| P935 | TAGATTTAACTTTATAACTTGTCATTTTTCTAGTTCCTTATATAGTTGCATGATCTGC |  |  |
| P936 | ATGACAAGTTATAAAGTTAAATCTA | PATH2472 | *cps43J* |
| P937 | TTAAATTTTCTTTAATTTCTTATAG |  |  |
| P938 | CTATAAGAAATTAAAGAAAATTTAAGAACCAATAAGTACGAGTATTGAAAGG | D39 | *cps2J* 3’ |
| P54 | CATAGCCGAAGGAAGGATTGT |  |  |
| For construction of ∆*cps2J<>cps45J* | | | |
| P51 | GAACATGGAAATGTGGAAGATGAG | D39 | *cps2J* 5’ |
| P939 | GATAATTTTTAGAAATTTTGCTCAATTTTCTAGTTCCTTATATAGTTGCATGATCTGC |  |  |
| P940 | TTGAGCAAAATTTCTAAAAATTATC | PATH656 | *cps45J* |
| P941 | TCATCTACTTTTTTTGAATCCTTTC |  |  |
| P942 | GAAAGGATTCAAAAAAAGTAGATGAGAACCAATAAGTACGAGTATTGAAAGG | D39 | *cps2J* 3’ |
| P54 | CATAGCCGAAGGAAGGATTGT |  |  |
| For construction of ∆*cps2J<>cps32AJ* | | | |
| P51 | GAACATGGAAATGTGGAAGATGAG | D39 | *cps2J* 5’ |
| P976 | CAACGCTCTTTTTTTGACTTTCCATTTTTCTAGTTCCTTATATAGTTGCATGATCTGC |  |  |
| P977 | ATGGAAAGTCAAAAAAAGAGCGTTG | PATH6653 | *cps32AJ* |
| P978 | TCATACTTTCCTTGCAAGAACATTT |  |  |
| P979 | AAATGTTCTTGCAAGGAAAGTATGAGAACCAATAAGTACGAGTATTGAAAGG | D39 | *cps2J* 3’ |
| P54 | CATAGCCGAAGGAAGGATTGT |  |  |
| For construction of ∆*cps2J<>cps32FJ* | | | |
| P51 | GAACATGGAAATGTGGAAGATGAG | D39 | *cps2J* 5’ |
| P976 | CAACGCTCTTTTTTTGACTTTCCATTTTTCTAGTTCCTTATATAGTTGCATGATCTGC |  |  |
| P977 | ATGGAAAGTCAAAAAAAGAGCGTTG | PATH2468 | *cps32FJ* |
| P978 | TCATACTTTCCTTGCAAGAACATTT |  |  |
| P979 | AAATGTTCTTGCAAGGAAAGTATGAGAACCAATAAGTACGAGTATTGAAAGG | D39 | *cps2J* 3’ |
| P54 | CATAGCCGAAGGAAGGATTGT |  |  |
| For construction of ∆*cps2J<>cps40J* | | | |
| P51 | GAACATGGAAATGTGGAAGATGAG | D39 | *cps2J* 5’ |
| P988 | TTGATTTTTCTTTCATGCTCATCAATTTTCTAGTTCCTTATATAGTTGCATGATCTGC |  |  |
| P989 | TTGATGAGCATGAAAGAAAAATCAA | PATH2469 | *cps40J* |
| P867 | TCATGAAATATCCTTATAAAAAATT |  |  |
| P868 | AATTTTTTATAAGGATATTTCATGAGAACCAATAAGTACGAGTATTGAAAGG | D39 | *cps2J* 3’ |
| P54 | CATAGCCGAAGGAAGGATTGT |  |  |
| For construction of ∆*cps2J<>cps44J* | | | |
| P51 | GAACATGGAAATGTGGAAGATGAG | D39 | *cps2J* 5’ |
| P990 | AAAAACTATTTTTTACACGACTCATTTTTCTAGTTCCTTATATAGTTGCATGATCTGC |  |  |
| P991 | ATGAGTCGTGTAAAAAATAGTTTTT | PATH2473 | *cps44J* |
| P992 | TTAATCATGATTTTTTTCCTCCAAA |  |  |
| P993 | TTTGGAGGAAAAAAATCATGATTAAGAACCAATAAGTACGAGTATTGAAAGG | D39 | *cps2J* 3’ |
| P54 | CATAGCCGAAGGAAGGATTGT |  |  |
| For construction of ∆*cps2J<>cps46J* | | | |
| P51 | GAACATGGAAATGTGGAAGATGAG | D39 | *cps2J* 5’ |
| P990 | AAAAACTATTTTTTACACGACTCATTTTTCTAGTTCCTTATATAGTTGCATGATCTGC |  |  |
| P991 | ATGAGTCGTGTAAAAAATAGTTTTT | PATH2474 | *cps46J* |
| P992 | TTAATCATGATTTTTTTCCTCCAAA |  |  |
| P993 | TTTGGAGGAAAAAAATCATGATTAAGAACCAATAAGTACGAGTATTGAAAGG | D39 | *cps2J* 3’ |
| P54 | CATAGCCGAAGGAAGGATTGT |  |  |
| For construction of ∆*cps2J<>cps47AJ* | | | |
| P51 | GAACATGGAAATGTGGAAGATGAG | D39 | *cps2J* 5’ |
| P943 | ATAAAAAATTTTTCAAAACTTTCATTTTTCTAGTTCCTTATATAGTTGCATGATCTGC |  |  |
| P944 | ATGAAAGTTTTGAAAAATTTTTTAT | PATH2476 | *cps47AJ* |
| P945 | CTAGGGGGTTTTTCTTATCATAGAA |  |  |
| P946 | TTCTATGATAAGAAAAACCCCCTAGGAACCAATAAGTACGAGTATTGAAAGG | D39 | *cps2J* 3’ |
| P54 | CATAGCCGAAGGAAGGATTGT |  |  |
| For construction of ∆*cps2J<>cps47FJ* | | | |
| P51 | GAACATGGAAATGTGGAAGATGAG | D39 | *cps2J* 5’ |
| P903 | GTACTTTCATACAAGCACACTCCACTTTTCTAGTTCCTTATATAGTTGCATGATCTGC |  |  |
| P904 | GTGGAGTGTGCTTGTATGAAAGTAC | PATH2475 | *cps47FJ* |
| P947 | CTAATTTTTCCTAATTACTTGTTTT |  |  |
| P948 | AAAACAAGTAATTAGGAAAAATTAGGAACCAATAAGTACGAGTATTGAAAGG | D39 | *cps2J* 3’ |
| P54 | CATAGCCGAAGGAAGGATTGT |  |  |
| For construction of ∆*cps2J<>cps48J* | | | |
| P51 | GAACATGGAAATGTGGAAGATGAG | D39 | *cps2J* 5’ |
| P949 | ATATGTAGTTTTTTAATAATTTCAATTTTCTAGTTCCTTATATAGTTGCATGATCTGC |  |  |
| P950 | TTGAAATTATTAAAAAACTACATAT | PATH1937 | *cps48J* |
| P951 | CTACTGTATCTTTATTTTTTTAAGT |  |  |
| P952 | ACTTAAAAAAATAAAGATACAGTAGGAACCAATAAGTACGAGTATTGAAAGG | D39 | *cps2J* 3’ |
| P54 | CATAGCCGAAGGAAGGATTGT |  |  |
| For construction of ∆*cps2J<>cps38J* | | | |
| P51 | GAACATGGAAATGTGGAAGATGAG | D39 | *cps2J* 5’ |
| P980 | CTAAACGTTTTTCTTTTGTTTCCATTTTTCTAGTTCCTTATATAGTTGCATGATCTGC |  |  |
| P981 | ATGGAAACAAAAGAAAAACGTTTAG | PATH112 | *cps38J* |
| P982 | TTATCTTTTTGATTTTTTGAATGAA |  |  |
| P983 | TTCATTCAAAAAATCAAAAAGATAAGAACCAATAAGTACGAGTATTGAAAGG | D39 | *cps2J* 3’ |
| P54 | CATAGCCGAAGGAAGGATTGT |  |  |
| For construction of ∆*cps2J<>cps25FJ* | | | |
| P51 | GAACATGGAAATGTGGAAGATGAG | D39 | *cps2J* 5’ |
| P980 | CTAAACGTTTTTCTTTTGTTTCCATTTTTCTAGTTCCTTATATAGTTGCATGATCTGC |  |  |
| P981 | ATGGAAACAAAAGAAAAACGTTTAG | PATH352 | *cps25FJ* |
| P982 | TTATCTTTTTGATTTTTTGAATGAA |  |  |
| P983 | TTCATTCAAAAAATCAAAAAGATAAGAACCAATAAGTACGAGTATTGAAAGG | D39 | *cps2J* 3’ |
| P54 | CATAGCCGAAGGAAGGATTGT |  |  |
| For construction of ∆*cps2J<>cps25AJ* | | | |
| P51 | GAACATGGAAATGTGGAAGATGAG | D39 | *cps2J* 5’ |
| P980 | CTAAACGTTTTTCTTTTGTTTCCATTTTTCTAGTTCCTTATATAGTTGCATGATCTGC |  |  |
| P981 | ATGGAAACAAAAGAAAAACGTTTAG | PATH2466 | *cps25AJ* |
| P982 | TTATCTTTTTGATTTTTTGAATGAA |  |  |
| P983 | TTCATTCAAAAAATCAAAAAGATAAGAACCAATAAGTACGAGTATTGAAAGG | D39 | *cps2J* 3’ |
| P54 | CATAGCCGAAGGAAGGATTGT |  |  |
| For construction of ∆*cps2J<>cps24BJ* | | | |
| P51 | GAACATGGAAATGTGGAAGATGAG | D39 | *cps2J* 5’ |
| P988 | TTGATTTTTCTTTCATGCTCATCAATTTTCTAGTTCCTTATATAGTTGCATGATCTGC |  |  |
| P989 | TTGATGAGCATGAAAGAAAAATCAA | PATH2465 | *cps24BJ* |
| P867 | TCATGAAATATCCTTATAAAAAATT |  |  |
| P868 | AATTTTTTATAAGGATATTTCATGAGAACCAATAAGTACGAGTATTGAAAGG | D39 | *cps2J* 3’ |
| P54 | CATAGCCGAAGGAAGGATTGT |  |  |
| For construction of ∆*cps2J<>cps24FJ* | | | |
| P51 | GAACATGGAAATGTGGAAGATGAG | D39 | *cps2J* 5’ |
| P869 | TTATTGATTTTTCTTTCATGCTCATTTTTCTAGTTCCTTATATAGTTGCATGATCTGC |  |  |
| P870 | ATGAGCATGAAAGAAAAATCAATAA | PATH20 | *cps24FJ* |
| P867 | TCATGAAATATCCTTATAAAAAATT |  |  |
| P868 | AATTTTTTATAAGGATATTTCATGAGAACCAATAAGTACGAGTATTGAAAGG | D39 | *cps2J* 3’ |
| P54 | CATAGCCGAAGGAAGGATTGT |  |  |
| For construction of ∆*cps2J<>cps6BJ* | | | |
| P51 | GAACATGGAAATGTGGAAGATGAG | D39 | *cps2J* 5’ |
| P223 | TTGTTATAAGAAACTTCAATTTCATTTTTCTAGTTCCTTATATAGTTGCATGATCTGC |  |  |
| P224 | ATGAAATTGAAGTTTCTTATAACAA | NUH0003 | *cps6BJ* |
| P225 | TTATTCAAATATTTTCTTTCTAAAA |  |  |
| P226 | TTTTAGAAAGAAAATATTTGAATAAGAACCAATAAGTACGAGTATTGAAAGG | D39 | *cps2J* 3’ |
| P54 | CATAGCCGAAGGAAGGATTGT |  |  |
| For construction of ∆*cps2J<>cps6CJ* | | | |
| P51 | GAACATGGAAATGTGGAAGATGAG | D39 | *cps2J* 5’ |
| P223 | TTGTTATAAGAAACTTCAATTTCATTTTTCTAGTTCCTTATATAGTTGCATGATCTGC |  |  |
| P224 | ATGAAATTGAAGTTTCTTATAACAA | NUH0004 | *cps6CJ* |
| P225 | TTATTCAAATATTTTCTTTCTAAAA |  |  |
| P226 | TTTTAGAAAGAAAATATTTGAATAAGAACCAATAAGTACGAGTATTGAAAGG | D39 | *cps2J* 3’ |
| P54 | CATAGCCGAAGGAAGGATTGT |  |  |
| For construction of ∆*cps2J<>cps6DJ* | | | |
| P51 | GAACATGGAAATGTGGAAGATGAG | D39 | *cps2J* 5’ |
| P223 | TTGTTATAAGAAACTTCAATTTCATTTTTCTAGTTCCTTATATAGTTGCATGATCTGC |  |  |
| P224 | ATGAAATTGAAGTTTCTTATAACAA | PATH3390 | *cps6DJ* |
| P225 | TTATTCAAATATTTTCTTTCTAAAA |  |  |
| P226 | TTTTAGAAAGAAAATATTTGAATAAGAACCAATAAGTACGAGTATTGAAAGG | D39 | *cps2J* 3’ |
| P54 | CATAGCCGAAGGAAGGATTGT |  |  |
| For construction of ∆*cps2J<>cps9VJ* | | | |
| P51 | GAACATGGAAATGTGGAAGATGAG | D39 | *cps2J* 5’ |
| P176 | CCTTAAACTTATTACTTATATCCATTTTTCTAGTTCCTTATATAGTTGCATGATCTGC |  |  |
| P177 | ATGGATATAAGTAATAAGTTTAAGG | PATH30 | *cps9VJ* |
| P178 | TCACCTACTAAACATCATTATTAAC |  |  |
| P179 | GTTAATAATGATGTTTAGTAGGTGAGAACCAATAAGTACGAGTATTGAAAGG | D39 | *cps2J* 3’ |
| P54 | CATAGCCGAAGGAAGGATTGT |  |  |
| For construction of ∆*cps2J<>cps9NJ* | | | |
| P51 | GAACATGGAAATGTGGAAGATGAG | D39 | *cps2J* 5’ |
| P172 | GTTTCAAATTTTTTTCAACATTCATTTTTCTAGTTCCTTATATAGTTGCATGATCTGC |  |  |
| P173 | ATGAATGTTGAAAAAAATTTGAAAC | PATH98 | *cps9NJ* |
| P174 | TTATTTTCTTATAACCGCGAAGACT |  |  |
| P175 | AGTCTTCGCGGTTATAAGAAAATAAGAACCAATAAGTACGAGTATTGAAAGG | D39 | *cps2J* 3’ |
| P54 | CATAGCCGAAGGAAGGATTGT |  |  |
| For construction of *∆cps2J<>cps20J* | | | |
| P51 | GAACATGGAAATGTGGAAGATGAG | D39 | *cps2J* 5’ |
| P1230 | AAGCATAGTTTTTTATTACTTTCATTTTTCTAGTTCCTTATATAGTTGCATGATCTGC |  |  |
| P1231 | ATGAAAGTAATAAAAAACTATGCTT | PATH682 | *cps20J* |
| P373 | CTAGTTTTTCCTAATAATTTGTTTT |  |  |
| P374 | AAAACAAATTATTAGGAAAAACTAGGAACCAATAAGTACGAGTATTGAAAGG | D39 | *cps2J* 3’ |
| P54 | CATAGCCGAAGGAAGGATTGT |  |  |
| For construction of *∆cps2J<>cps21J* | | | |
| P51 | GAACATGGAAATGTGGAAGATGAG | D39 | *cps2J* 5’ |
| P1232 | AGGCGTAATTTTTAAGAACTTTCATTTTTCTAGTTCCTTATATAGTTGCATGATCTGC |  |  |
| P1233 | ATGAAAGTTCTTAAAAATTACGCCT | PATH57 | *cps21J* |
| P1234 | TTAAATCTCCCTATTTTTTAATAGT |  |  |
| P1235 | ACTATTAAAAAATAGGGAGATTTAAGAACCAATAAGTACGAGTATTGAAAGG | D39 | *cps2J* 3’ |
| P54 | CATAGCCGAAGGAAGGATTGT |  |  |
| For construction of ∆*cps2J<>cps22AJ* | | | |
| P51 | GAACATGGAAATGTGGAAGATGAG | D39 | *cps2J* 5’ |
| P1236 | TAATAAGTGAATTTCTGGTTGACATTTTTCTAGTTCCTTATATAGTTGCATGATCTGC |  |  |
| P1237 | ATGTCAACCAGAAATTCACTTATTA | NUH0015 | *cps22AJ* |
| P1238 | TTATCTCTTTCTCTTTATAATACCT |  |  |
| P1239 | AGGTATTATAAAGAGAAAGAGATAAGAACCAATAAGTACGAGTATTGAAAGG | D39 | *cps2J* 3’ |
| P54 | CATAGCCGAAGGAAGGATTGT |  |  |
| For construction of ∆*cps2J<>cps22FJ* | | | |
| P51 | GAACATGGAAATGTGGAAGATGAG | D39 | *cps2J* 5’ |
| P1236 | TAATAAGTGAATTTCTGGTTGACATTTTTCTAGTTCCTTATATAGTTGCATGATCTGC |  |  |
| P1237 | ATGTCAACCAGAAATTCACTTATTA | PATH115 | *cps22FJ* |
| P1238 | TTATCTCTTTCTCTTTATAATACCT |  |  |
| P1239 | AGGTATTATAAAGAGAAAGAGATAAGAACCAATAAGTACGAGTATTGAAAGG | D39 | *cps2J* 3’ |
| P54 | CATAGCCGAAGGAAGGATTGT |  |  |
| For construction of ∆*cps2J<>cps27J* | | | |
| P51 | GAACATGGAAATGTGGAAGATGAG | D39 | *cps2J* 5’ |
| P1240 | GATTACTTATTTTTGTAGTACTCATTTTTCTAGTTCCTTATATAGTTGCATGATCTGC |  |  |
| P1241 | ATGAGTACTACAAAAATAAGTAATC | PATH2467 | *cps27J* |
| P1242 | TTATAATTTTATGTGTCTTATTTTT |  |  |
| P1243 | AAAAATAAGACACATAAAATTATAAGAACCAATAAGTACGAGTATTGAAAGG | D39 | *cps2J* 3’ |
| P54 | CATAGCCGAAGGAAGGATTGT |  |  |
| For construction of ∆*cps2J<>cps28AJ* | | | |
| P51 | GAACATGGAAATGTGGAAGATGAG | D39 | *cps2J* 5’ |
| P1244 | ATATTTTTTTCATTACAGATTCCAATTTTCTAGTTCCTTATATAGTTGCATGATCTGC |  |  |
| P1245 | TTGGAATCTGTAATGAAAAAAATAT | PATH9002 | *cps28AJ* |
| P1246 | TCACTGTTTTTTTATCATGACTATC |  |  |
| P1247 | GATAGTCATGATAAAAAAACAGTGAGAACCAATAAGTACGAGTATTGAAAGG | D39 | *cps2J* 3’ |
| P54 | CATAGCCGAAGGAAGGATTGT |  |  |
| For construction of ∆*cps2J<>cps28FJ* | | | |
| P51 | GAACATGGAAATGTGGAAGATGAG | D39 | *cps2J* 5’ |
| P1244 | ATATTTTTTTCATTACAGATTCCAATTTTCTAGTTCCTTATATAGTTGCATGATCTGC |  |  |
| P1245 | TTGGAATCTGTAATGAAAAAAATAT | PATH382 | *cps28FJ* |
| P1246 | TCACTGTTTTTTTATCATGACTATC |  |  |
| P1247 | GATAGTCATGATAAAAAAACAGTGAGAACCAATAAGTACGAGTATTGAAAGG | D39 | *cps2J* 3’ |
| P54 | CATAGCCGAAGGAAGGATTGT |  |  |
| For construction of *∆cps2J<>cps12AJ* | | | |
| P51 | GAACATGGAAATGTGGAAGATGAG | D39 | *cps2J* 5’ |
| P990 | AAAAACTATTTTTTACACGACTCATTTTTCTAGTTCCTTATATAGTTGCATGATCTGC |  |  |
| P991 | ATGAGTCGTGTAAAAAATAGTTTTT | PATH1706 | *cps12AJ* |
| P992 | TTAATCATGATTTTTTTCCTCCAAA |  |  |
| P993 | TTTGGAGGAAAAAAATCATGATTAAGAACCAATAAGTACGAGTATTGAAAGG | D39 | *cps2J* 3’ |
| P54 | CATAGCCGAAGGAAGGATTGT |  |  |
| For construction of ∆*cps2J<>cps12BJ* | | | |
| P51 | GAACATGGAAATGTGGAAGATGAG | D39 | *cps2J* 5’ |
| P990 | AAAAACTATTTTTTACACGACTCATTTTTCTAGTTCCTTATATAGTTGCATGATCTGC |  |  |
| P991 | ATGAGTCGTGTAAAAAATAGTTTTT | PATH2479 | *cps12BJ* |
| P992 | TTAATCATGATTTTTTTCCTCCAAA |  |  |
| P993 | TTTGGAGGAAAAAAATCATGATTAAGAACCAATAAGTACGAGTATTGAAAGG | D39 | *cps2J* 3’ |
| P54 | CATAGCCGAAGGAAGGATTGT |  |  |
| For construction of ∆*cps2J<>cps12FJ* | | | |
| P51 | GAACATGGAAATGTGGAAGATGAG | D39 | *cps2J* 5’ |
| P990 | AAAAACTATTTTTTACACGACTCATTTTTCTAGTTCCTTATATAGTTGCATGATCTGC |  |  |
| P991 | ATGAGTCGTGTAAAAAATAGTTTTT | PATH122 | *cps12FJ* |
| P992 | TTAATCATGATTTTTTTCCTCCAAA |  |  |
| P993 | TTTGGAGGAAAAAAATCATGATTAAGAACCAATAAGTACGAGTATTGAAAGG | D39 | *cps2J* 3’ |
| P54 | CATAGCCGAAGGAAGGATTGT |  |  |
| For construction of ∆*cps2J<>cps18BJ* | | | |
| P51 | GAACATGGAAATGTGGAAGATGAG | D39 | *cps2J* 5’ |
| P1267 | CAATGTTTTTCTTTAAATTAGGCAATTTTCTAGTTCCTTATATAGTTGCATGATCTGC |  |  |
| P1268 | TTGCCTAATTTAAAGAAAAACATTG | PATH269 | *cps18BJ* |
| P1269 | TTATTTCCTTTTAAATAAATGAGTA |  |  |
| P1270 | TACTCATTTATTTAAAAGGAAATAAGAACCAATAAGTACGAGTATTGAAAGG | D39 | *cps2J* 3’ |
| P54 | CATAGCCGAAGGAAGGATTGT |  |  |
| For construction of ∆*cps2J<>cps18CJ* | | | |
| P51 | GAACATGGAAATGTGGAAGATGAG | D39 | *cps2J* 5’ |
| P1267 | CAATGTTTTTCTTTAAATTAGGCAATTTTCTAGTTCCTTATATAGTTGCATGATCTGC |  |  |
| P1268 | TTGCCTAATTTAAAGAAAAACATTG | NUH0012 | *cps18CJ* |
| P1269 | TTATTTCCTTTTAAATAAATGAGTA |  |  |
| P1270 | TACTCATTTATTTAAAAGGAAATAAGAACCAATAAGTACGAGTATTGAAAGG | D39 | *cps2J* 3’ |
| P54 | CATAGCCGAAGGAAGGATTGT |  |  |
| For construction of ∆*cps2J<>cps18FJ* | | | |
| P51 | GAACATGGAAATGTGGAAGATGAG | D39 | *cps2J* 5’ |
| P1267 | CAATGTTTTTCTTTAAATTAGGCAATTTTCTAGTTCCTTATATAGTTGCATGATCTGC |  |  |
| P1268 | TTGCCTAATTTAAAGAAAAACATTG | PATH4599 | *cps18FJ* |
| P1265 | TTACTTCCTTTTAAATAAATGAGTA |  |  |
| P1266 | TACTCATTTATTTAAAAGGAAGTAAGAACCAATAAGTACGAGTATTGAAAGG | D39 | *cps2J* 3’ |
| P54 | CATAGCCGAAGGAAGGATTGT |  |  |
| For construction of ∆*cps2J<>cps19AJ* | | | |
| P51 | GAACATGGAAATGTGGAAGATGAG | D39 | *cps2J* 5’ |
| P1271 | GTATATTTTTAATTTTGCTATTCATTTTTCTAGTTCCTTATATAGTTGCATGATCTGC |  |  |
| P1272 | ATGAATAGCAAAATTAAAAATATAC | NUH0013 | *cps19AJ* |
| P1273 | TTAATTTGATGTTTTTTTACTAGCT |  |  |
| P1274 | AGCTAGTAAAAAAACATCAAATTAAGAACCAATAAGTACGAGTATTGAAAGG | D39 | *cps2J* 3’ |
| P54 | CATAGCCGAAGGAAGGATTGT |  |  |
| For construction of ∆*cps2J<>cps18AJ* | | | |
| P51 | GAACATGGAAATGTGGAAGATGAG | D39 | *cps2J* 5’ |
| P1263 | CAATATTTTTCTTTAAACTAGGCAATTTTCTAGTTCCTTATATAGTTGCATGATCTGC |  |  |
| P1264 | TTGCCTAGTTTAAAGAAAAATATTG | PATH4560 | *cps18AJ* |
| P1265 | TTACTTCCTTTTAAATAAATGAGTA |  |  |
| P1266 | TACTCATTTATTTAAAAGGAAGTAAGAACCAATAAGTACGAGTATTGAAAGG | D39 | *cps2J* 3’ |
| P54 | CATAGCCGAAGGAAGGATTGT |  |  |
| For construction of ∆*cps2J<>cps19FJ* | | | |
| P51 | GAACATGGAAATGTGGAAGATGAG | D39 | *cps2J* 5’ |
| P1279 | TTATATTTTTAATTTTAGTATTCATTTTTCTAGTTCCTTATATAGTTGCATGATCTGC |  |  |
| P1280 | ATGAATACTAAAATTAAAAATATAA | NUH0014 | *cps19FJ* |
| P1281 | TTATGATATTTTTTTATGATTTTTA |  |  |
| P1282 | TAAAAATCATAAAAAAATATCATAAGAACCAATAAGTACGAGTATTGAAAGG | D39 | *cps2J* 3’ |
| P54 | CATAGCCGAAGGAAGGATTGT |  |  |
| For construction of ∆*cps2J<>cps19BJ* | | | |
| P51 | GAACATGGAAATGTGGAAGATGAG | D39 | *cps2J* 5’ |
| P1275 | TCAACTTTATGGATTTATTCCCCATTTTTCTAGTTCCTTATATAGTTGCATGATCTGC |  |  |
| P1276 | ATGGGGAATAAATCCATAAAGTTGA | PATH2606 | *cps19BJ* |
| P1277 | TTACTTCTTCTTTGCAAGAAGAGAA |  |  |
| P1278 | TTCTCTTCTTGCAAAGAAGAAGTAAGAACCAATAAGTACGAGTATTGAAAGG | D39 | *cps2J* 3’ |
| P54 | CATAGCCGAAGGAAGGATTGT |  |  |
| For construction of ∆*cps2J<>cps19CJ* | | | |
| P51 | GAACATGGAAATGTGGAAGATGAG | D39 | *cps2J* 5’ |
| P1275 | TCAACTTTATGGATTTATTCCCCATTTTTCTAGTTCCTTATATAGTTGCATGATCTGC |  |  |
| P1276 | ATGGGGAATAAATCCATAAAGTTGA | PATH2463 | *cps19CJ* |
| P1277 | TTACTTCTTCTTTGCAAGAAGAGAA |  |  |
| P1278 | TTCTCTTCTTGCAAAGAAGAAGTAAGAACCAATAAGTACGAGTATTGAAAGG | D39 | *cps2J* 3’ |
| P54 | CATAGCCGAAGGAAGGATTGT |  |  |
| For construction of ∆*cps2J<>cps16FJ* | | | |
| P51 | GAACATGGAAATGTGGAAGATGAG | D39 | *cps2J* 5’ |
| P1297 | ATATTTTTTTCATGACAGATTCCAATTTTCTAGTTCCTTATATAGTTGCATGATCTGC |  |  |
| P1298 | TTGGAATCTGTCATGAAAAAAATAT | PATH1702 | *cps16FJ* |
| P1299 | TCACTGTTGTTTAATGATAGCTATC |  |  |
| P1300 | GATAGCTATCATTAAACAACAGTGAGAACCAATAAGTACGAGTATTGAAAGG | D39 | *cps2J* 3’ |
| P54 | CATAGCCGAAGGAAGGATTGT |  |  |
| For construction of ∆*cps2J<>cps17AJ* | | | |
| P51 | GAACATGGAAATGTGGAAGATGAG | D39 | *cps2J* 5’ |
| P1301 | AGCTTAAACTTTTTTCGTTATTCATTTTTCTAGTTCCTTATATAGTTGCATGATCTGC |  |  |
| P1302 | ATGAATAACGAAAAAAGTTTAAGCT | PATH2480 | *cps17AJ* |
| P1303 | CTACTCAGTGCTCAAAAATCTATTT |  |  |
| P1304 | AAATAGATTTTTGAGCACTGAGTAGGAACCAATAAGTACGAGTATTGAAAGG | D39 | *cps2J* 3’ |
| P54 | CATAGCCGAAGGAAGGATTGT |  |  |
| For construction of ∆*cps2J<>cps17FJ* | | | |
| P51 | GAACATGGAAATGTGGAAGATGAG | D39 | *cps2J* 5’ |
| P1305 | TAGTATTAACAAGTCTATTACTCATTTTTCTAGTTCCTTATATAGTTGCATGATCTGC |  |  |
| P1306 | ATGAGTAATAGACTTGTTAATACTA | PATH680 | *cps17FJ* |
| P1307 | TTATTTTAATAATTTCGACTTGATT |  |  |
| P1308 | AATCAAGTCGAAATTATTAAAATAAGAACCAATAAGTACGAGTATTGAAAGG | D39 | *cps2J* 3’ |
| P54 | CATAGCCGAAGGAAGGATTGT |  |  |
| For construction of ∆*cps2J<>cps24AJ* | | | |
| P51 | GAACATGGAAATGTGGAAGATGAG | D39 | *cps2J* 5’ |
| P984 | CTGAACGTTGCTTTCCTATATTCATTTTTCTAGTTCCTTATATAGTTGCATGATCTGC |  |  |
| P985 | ATGAATATAGGAAAGCAACGTTCAG | PATH2464 | *cps24AJ* |
| P986 | TCATTTAATTTCTTTAATAATTCTC |  |  |
| P987 | GAGAATTATTAAAGAAATTAAATGAGAACCAATAAGTACGAGTATTGAAAGG | D39 | *cps2J* 3’ |
| P54 | CATAGCCGAAGGAAGGATTGT |  |  |
| For construction of ∆*cps2J<>cps4J* | | | |
| P51 | GAACATGGAAATGTGGAAGATGAG | D39 | *cps2J* 5’ |
| P853 | TAAATGAAATTCTATCAACTTTCACTTTTCTAGTTCCTTATATAGTTGCATGATCTGC |  |  |
| P854 | GTGAAAGTTGATAGAATTTCATTTA | CCUG37285 | *cps4J* |
| P855 | TTACTTACTCCTAACAAATAATGAA |  |  |
| P856 | TTCATTATTTGTTAGGAGTAAGTAAGAACCAATAAGTACGAGTATTGAAAGG | D39 | *cps2J* 3’ |
| P54 | CATAGCCGAAGGAAGGATTGT |  |  |
| For construction of ∆*cps2J<>cps29J* | | | |
| P51 | GAACATGGAAATGTGGAAGATGAG | D39 | *cps2J* 5’ |
| P1248 | AGGCATAGTTTTTTAATACTTTCATTTTTCTAGTTCCTTATATAGTTGCATGATCTGC |  |  |
| P1249 | ATGAAAGTATTAAAAAACTATGCCT | PATH4478 | *cps29J* |
| P1250 | TTATGCTCCTTTATTTTTTAATAAT |  |  |
| P1251 | ATTATTAAAAAATAAAGGAGCATAAGAACCAATAAGTACGAGTATTGAAAGG | D39 | *cps2J* 3’ |
| P54 | CATAGCCGAAGGAAGGATTGT |  |  |
| For construction of ∆*cps2J<>cps42J* | | | |
| P51 | GAACATGGAAATGTGGAAGATGAG | D39 | *cps2J* 5’ |
| P371 | ATAAGTAGTTTTTTAATACCTTCATTTTTCTAGTTCCTTATATAGTTGCATGATCTGC |  |  |
| P372 | ATGAAGGTATTAAAAAACTACTTAT | CCUG6568 | *cps42J* |
| P373 | CTAGTTTTTCCTAATAATTTGTTTT |  |  |
| P374 | AAAACAAATTATTAGGAAAAACTAGGAACCAATAAGTACGAGTATTGAAAGG | D39 | *cps2J* 3’ |
| P54 | CATAGCCGAAGGAAGGATTGT |  |  |
| For construction of ∆*cps*::P-*sacB*-*kan*-*rpsL^+^* | | | |
| P118 | GGTGAGTAGGGAAGAAGAGGTA | D39 | *cps* 5’ |
| P16 | CATTATCCATTAAAAATCAAACGGATCCTAGATTAACACCTATACATTGAACATCTTACG |  |  |
| P1 | TAGGATCCGTTTGATTTTTAATGGATAATG | NUS0067 | P-*sacB*-*kan*-*rpsL^+^* |
| P2 | GGGCCCCTTTCCTTATGCTTTTG |  |  |
| P59 | GTCCAAAAGCATAAGGAAAGGGGCCCTTTGTTATTCCAACTTGGCAAGATGCATTGCAAG | D39 | *cps* 3’ |
| P117 | GGCGCGTTCAGCTAGATTAT |  |  |
| For construction of ∆*bgaA*::P_Zn_-*cps33BJ* | | | |
| P23 | CCGTAGAACCACTATCACAAG | AKF_Spn024 | *bgaA*-P_zn_ 5’ |
| P147 | ATTTCTCATTCCTTTGTTATAATAG |  |  |
| P780 | CTATTATAACAAAGGAATGAGAAATATGAAAGTACTAAAAAATTACGCC | PATH1945 | *cps33BJ* |
| P365 | TTAGTTTTTCCTAATAATTTGTTTT |  |  |
| P781 | AAAACAAATTATTAGGAAAAACTAATTAGCTCTTCTAGGTTTGAGTGCAGG | D39 | *bgaA* 3’ |
| P6 | TGCATGGTTACGATAGTCTTGG |  |  |
| For construction of ∆*cps33BJ*::P-*kan*-*rpsL^+^* | | | |
| P817 | TCAATGGTCTGGTGGTCAATTA | PATH1945 | *cps33BJ* 5’ |
| P818 | CATTATCCATTAAAAATCAAACGGATCCTAGTTTTATTAATTTATTTCACTTGCTTTAAA |  |  |
| P1 | TAGGATCCGTTTGATTTTTAATGGATAATG | HMS0001 | P-*kan-rpsL^+^* |
| P2 | GGGCCCCTTTCCTTATGCTTTTG |  |  |
| P819 | CAAAAGCATAAGGAAAGGGGCCCAATGAGAAAAATTCGAAATATCAACCTAGATTTAC | PATH1945 | *cps33BJ* 3’ |
| P820 | AAATCCCTACGCTTACAACTTT |  |  |
| For construction of ∆*cps33BJ*<>*cps2J* | | | |
| P817 | TCAATGGTCTGGTGGTCAATTA | PATH1945 | *cps33BJ* 5’ |
| P895 | GTAAGTAATTATATCTTCTACTCAAGTTTTATTAATTTATTTCACTTGCTTTAAA |  |  |
| P40 | TTGAGTAGAAGATATAATTACTTAC | D39 | *cps2J* |
| P41 | TTATGTTAGAAACTTTTTTAATTCA |  |  |
| P896 | TGAATTAAAAAAGTTTCTAACATAAAATGAGAAAAATTCGAAATATCAACCTAGATTTAC | PATH1945 | *cps33BJ* 3’ |
| P820 | AAATCCCTACGCTTACAACTTT |  |  |
| For construction of ∆*cps33BJ*::P-*erm* | | | |
| P817 | TCAATGGTCTGGTGGTCAATTA | PATH1945 | *cps33BJ* 5’ |
| P818 | CATTATCCATTAAAAATCAAACGGATCCTAGTTTTATTAATTTATTTCACTTGCTTTAAA |  |  |
| P1 | TAGGATCCGTTTGATTTTTAATGGATAATG | NUS0013 | P-*erm* |
| P2 | GGGCCCCTTTCCTTATGCTTTTG |  |  |
| P819 | CAAAAGCATAAGGAAAGGGGCCCAATGAGAAAAATTCGAAATATCAACCTAGATTTAC | PATH1945 | *cps33BJ* 3’ |
| P820 | AAATCCCTACGCTTACAACTTT |  |  |
| For construction of ∆*cps2J*<>*tacF* | | | |
| P51 | GAACATGGAAATGTGGAAGATGAG | D39 | *cps2J* 5’ |
| P1094 | GAGCATTTAATTTTATACTTTTCATAAAGTTGCTTAAAGTTAGTAATCC |  |  |
| P1095 | ATGAAAAGTATAAAATTAAATGCTC | D39 | *tacF* |
| P1096 | CTATGATTTTTTAAATTTATTTTTT |  |  |
| P1097 | AAAAAATAAATTTAAAAAATCATAGATGTTATTTTATAAAGAAATAAAGAGTATTATTGG | D39 | *cps2J* 3’ |
| P54 | CATAGCCGAAGGAAGGATTGT |  |  |
| For construction of ∆*cps2J*<>*ytgP* | | | |
| P36 | GAACATGGAAATGTGGAAGATGAG | D39 | *cps2J* 5’ |
| P1568 | GCTGGTGATTGTTTTCGTGCGACATAAAGTTGCTTAAAGTTAGTAATCCAATATTTTTTA |  |  |
| P1100 | ATGTCGCACGAAAACAATCACCAGC | D39 | *ytgp* |
| P1099 | TTACGAAAGCTTAAATTTTGCTCGC |  |  |
| P1569 | GCGAGCAAAATTTAAGCTTTCGTAAATGTTATTTTATAAAGAAATAAAGAGTATTATTGG | D39 | *cps2J* 3’ |
| P300 | CATAGCCGAAGGAAGGATTGT |  |  |
| For construction of ∆*tacF*::P-*erm* | | | |
| P972 | CATGCATCTGTCAAGATTTCCTTC | D39 | *tacF* |
| P1092 | CATTATCCATTAAAAATCAAACGGATCCTAAATAATATTCAAGACACGAATTCCC |  |  |
| P1 | TAGGATCCGTTTGATTTTTAATGGATAATG | P-*erm* cassette | P-*erm* |
| P2 | GGGCCCCTTTCCTTATGCTTTTG |  |  |
| P1093 | CAAAAGCATAAGGAAAGGGGCCCATTTTCTATGAATTTTTAAACCATGTCCTAGCC | D39 | *tacF* |
| P975 | ACGCTCATAATCCTCACGATAAA |  |  |
| For construction of ∆*ytgP*::P-*erm* | | | |
| P1289 | ATCCCAGAACCAGAGCAATC | D39 | *ytgP* 5’ |
| P1290 | CATTATCCATTAAAAATCAAACGGATCCTACGTTAGCCAAGCAGTCCCCCGTAACATC |  |  |
| P1 | TAGGATCCGTTTGATTTTTAATGGATAATG | P-*erm* cassette | P-*erm* |
| P2 | GGGCCCCTTTCCTTATGCTTTTG |  |  |
| P1291 | CAAAAGCATAAGGAAAGGGGCCCGATAAGGTAATAGGAAAAGCCCAAGCAGATC | D39 | *ytgP* 3’ |
| P1292 | TTGAGAACTGCTTCCTGAGTC |  |  |
| For construction of ∆*cps2J*<>*cps10AJ-*FLAG | | | |
| P748 | CGTTGACTTCTTTTTTAGGAGCTCAG | NUS0549 | *cps10AJ*-FLAG 5’ |
| P1219 | TTATTTGTCATCATCATCTTTATAATCTTTTTTTATGATTTGTTTTAATTCTTTCACATC |  |  |
| P1223 | GATTATAAAGATGATGATGACAAATAAGAACCAATAAGTACGAGTATTGAAAGGAGAAAA | NUS0549 | *cps10AJ*-FLAG 3’ |
| P54 | CATAGCCGAAGGAAGGATTGT |  |  |
| For construction of ∆*cps2J*<>*cps10BJ-*FLAG | | | |
| P748 | CGTTGACTTCTTTTTTAGGAGCTCAG | NUS0550 | *cps10BJ*-FLAG 5’ |
| P1219 | TTATTTGTCATCATCATCTTTATAATCTTTTTTTATGATTTGTTTTAATTCTTTCACATC |  |  |
| P1223 | GATTATAAAGATGATGATGACAAATAAGAACCAATAAGTACGAGTATTGAAAGGAGAAAA | NUS0550 | *cps10BJ*-FLAG 3’ |
| P54 | CATAGCCGAAGGAAGGATTGT |  |  |
| For construction of ∆*cps2J*<>*cps7AJ*-FLAG | | | |
| P748 | CGTTGACTTCTTTTTTAGGAGCTCAG | NUS0539 | *cps7AJ*-FLAG 5’ |
| P1221 | TCATTTGTCATCATCATCTTTATAATCAAAAAATCTTGTGTTTTTATTTAATAATTTTAT |  |  |
| P1225 | GATTATAAAGATGATGATGACAAATGAGAACCAATAAGTACGAGTATTGAAAGGAGAAAA | NUS0539 | *cps7AJ*-FLAG 3’ |
| P54 | CATAGCCGAAGGAAGGATTGT |  |  |
| For construction of ∆*cps2J*<>*cps23FJ*-FLAG | | | |
| P748 | CGTTGACTTCTTTTTTAGGAGCTCAG | NUS0089 | *cps23FJ*-FLAG 5’ |
| P1220 | CTATTTGTCATCATCATCTTTATAATCATTTCTTTTGAAAATTTTTAAATATAAAGGTTT |  |  |
| P1224 | GATTATAAAGATGATGATGACAAATAGGAACCAATAAGTACGAGTATTGAAAGGAGAAAA | NUS0089 | *cps23FJ*-FLAG 3’ |
| P54 | CATAGCCGAAGGAAGGATTGT |  |  |
| For construction of ∆*cps2J*<>*cps33BJ*-FLAG | | | |
| P748 | CGTTGACTTCTTTTTTAGGAGCTCAG | NUS0266 | *cps33BJ*-FLAG 5’ |
| P1218 | TTATTTGTCATCATCATCTTTATAATCGTTTTTCCTAATAATTTGTTTTAATTCTTTCAC |  |  |
| P1223 | GATTATAAAGATGATGATGACAAATAAGAACCAATAAGTACGAGTATTGAAAGGAGAAAA | NUS0266 | *cps33BJ*-FLAG 3’ |
| P54 | CATAGCCGAAGGAAGGATTGT |  |  |
| For construction of ∆*cps2J*<>*cps48J*-FLAG | | | |
| P748 | CGTTGACTTCTTTTTTAGGAGCTCAG | NUS0659 | *cps48J*-FLAG 5’ |
| P1222 | CTATTTGTCATCATCATCTTTATAATCCTGTATCTTTATTTTTTTAAGTATAGTATTTAC |  |  |
| P1224 | GATTATAAAGATGATGATGACAAATAGGAACCAATAAGTACGAGTATTGAAAGGAGAAAA | NUS0659 | *cps48J*-FLAG 3’ |
| P54 | CATAGCCGAAGGAAGGATTGT |  |  |
| For construction of ∆*cps2J*<>*cps2J*-FLAG | | | |
| P748 | CGTTGACTTCTTTTTTAGGAGCTCAG | D39 | *cps2J*-FLAG 5’ |
| P1344 | TTATTTGTCATCATCATCTTTATAATCTGTTAGAAACTTTTTTAATTCACCAATAATACT |  |  |
| P1223 | GATTATAAAGATGATGATGACAAATAAGAACCAATAAGTACGAGTATTGAAAGGAGAAAA | D39 | *cps2J*-FLAG 3’ |
| P54 | CATAGCCGAAGGAAGGATTGT |  |  |
| For construction of ∆*cps2J*<>*cps10AJ* (I101T) | | | |
| P51 | GAACATGGAAATGTGGAAGATGAG | NUS0549 | *cps10AJ(I101T)* 5' |
| P1066 | TTTGCATAAAAGGAAGAGTTAGACAAAGAACAAGG |  |  |
| P1065 | CCTTGTTCTTTGTCTAACTCTTCCTTTTATGCAAA | NUS0549 | *cps10AJ(I101T)* 3' |
| P54 | CATAGCCGAAGGAAGGATTGT |  |  |
| For construction of ∆*cps2J*<>*cps10AJ* (F109V) | | | |
| P51 | GAACATGGAAATGTGGAAGATGAG | NUS0549 | *cps10AJ(F109V)* 5' |
| P1068 | GCCTAGAATATAGGCGACTGGATTTTGCATAAAAG |  |  |
| P1067 | CTTTTATGCAAAATCCAGTCGCCTATATTCTAGGC | NUS0549 | *cps10AJ(F109V)* 3' |
| P54 | CATAGCCGAAGGAAGGATTGT |  |  |
| For construction of ∆*cps2J*<>*cps10AJ* (Y192H) | | | |
| P51 | GAACATGGAAATGTGGAAGATGAG | NUS0549 | *cps10AJ(Y192H)* 5' |
| P1070 | ATAGCTTAAATCAAAATGTGGTTTCCCAATAAATT |  |  |
| P1069 | AATTTATTGGGAAACCACATTTTGATTTAAGCTAT | NUS0549 | *cps10AJ(Y192H)* 3' |
| P54 | CATAGCCGAAGGAAGGATTGT |  |  |
| For construction of ∆*cps2J*<>*cps10AJ* (E222N) | | | |
| P51 | GAACATGGAAATGTGGAAGATGAG | NUS0549 | *cps10AJ(E222N)* 5' |
| P1072 | CACCAAGCATCGTACGATTTAGAGTAACATATAAG |  |  |
| P1071 | CTTATATGTTACTCTAAATCGTACGATGCTTGGTG | NUS0549 | *cps10AJ(E222N)* 3' |
| P54 | CATAGCCGAAGGAAGGATTGT |  |  |
| For construction of ∆*cps2J*<>*cps10AJ* (A356V) | | | |
| P51 | GAACATGGAAATGTGGAAGATGAG | NUS0549 | *cps10AJ(A356V)* 3' |
| P1074 | CACTGATAATTGCAGAAACTGTTGTTGAAATCATG |  |  |
| P1073 | CATGATTTCAACAACAGTTTCTGCAATTATCAGTG | NUS0549 | *cps10AJ(A356V)* 3' |
| P54 | CATAGCCGAAGGAAGGATTGT |  |  |
| For construction of ∆*cps2J*<>*cps10BJ* (T101I) | | | |
| P51 | GAACATGGAAATGTGGAAGATGAG | NUS0550 | *cps10BJ* (*T101I*) 5' |
| P1154 | TTTGCATAAAAGGAAGAATTAGACAAAGAACAAGG |  |  |
| P1155 | CCTTGTTCTTTGTCTAATTCTTCCTTTTATGCAAA | NUS0550 | *cps10BJ* (*T101I*) 3' |
| P54 | CATAGCCGAAGGAAGGATTGT |  |  |
| For construction of ∆*cps2J*<>*cps10BJ* (V109F) | | | |
| P51 | GAACATGGAAATGTGGAAGATGAG | NUS0550 | *cps10BJ* (*V109F*) 5' |
| P1156 | GCCTAGAATATAGGCGAATGGATTTTGCATAAAAG |  |  |
| P1157 | CTTTTATGCAAAATCCATTCGCCTATATTCTAGGC | NUS0550 | *cps10BJ* (*V109F*) 3' |
| P54 | CATAGCCGAAGGAAGGATTGT |  |  |
| For construction of ∆*cps2J*<>*cps10BJ* (H192Y) | | | |
| P51 | GAACATGGAAATGTGGAAGATGAG | NUS0550 | *cps10BJ* (*H192Y*) 5' |
| P1158 | ATAGCTTAAATCAAAATATGGTTTCCCAATAAATT |  |  |
| P1159 | AATTTATTGGGAAACCATATTTTGATTTAAGCTAT | NUS0550 | *cps10BJ* (*H192Y*) 3' |
| P54 | CATAGCCGAAGGAAGGATTGT |  |  |
| For construction of ∆*cps2J*<>*cps10BJ* (N222E) | | | |
| P51 | GAACATGGAAATGTGGAAGATGAG | NUS0550 | *cps10BJ* (*N222E*) 5' |
| P1160 | CACCAAGCATCGTACGTTCTAGAGTAACATATAAG |  |  |
| P1161 | CTTATATGTTACTCTAGAACGTACGATGCTTGGTG | NUS0550 | *cps10BJ* (*N222E*) 3' |
| P54 | CATAGCCGAAGGAAGGATTGT |  |  |
| For construction of ∆*cps2J*<>*cps10BJ* (V356A) | | | |
| P51 | GAACATGGAAATGTGGAAGATGAG | NUS0550 | *cps10BJ* (*V356A*) 5' |
| P1162 | CACTGATAATTGCAGAAGCTGTTGTTGAAATCATG |  |  |
| P1163 | CATGATTTCAACAACAGCTTCTGCAATTATCAGTG | NUS0550 | *cps10BJ* (*V356A*) 3' |
| P54 | CATAGCCGAAGGAAGGATTGT |  |  |
| For construction of ∆*cps2J*<>*cps10AJ*(I101T_Y192H) | | | |
| P51 | GAACATGGAAATGTGGAAGATGAG | NUS0697 | *cps10AJ(I101T_Y192H)* 5' |
| P1070 | ATAGCTTAAATCAAAATGTGGTTTCCCAATAAATT |  |  |
| P1069 | AATTTATTGGGAAACCACATTTTGATTTAAGCTAT | NUS0697 | *cps10AJ(I101T_Y192H)* 3' |
| P54 | CATAGCCGAAGGAAGGATTGT |  |  |
| For construction of ∆*cps2J*<>*cps10AJ*(I101T_A356V) | | | |
| P51 | GAACATGGAAATGTGGAAGATGAG | NUS0697 | *cps10AJ(I101T_A356V)* 5' |
| P1074 | CACTGATAATTGCAGAAACTGTTGTTGAAATCATG |  |  |
| P1073 | CATGATTTCAACAACAGTTTCTGCAATTATCAGTG | NUS0697 | *cps10AJ(I101T_A356V)* 3' |
| P54 | CATAGCCGAAGGAAGGATTGT |  |  |
| For construction of ∆*cps2J*<>*cps10AJ*(F109V_E222N) | | | |
| P51 | GAACATGGAAATGTGGAAGATGAG | NUS0698 | *cps10AJ(F109V_E222N)* 5' |
| P1072 | CACCAAGCATCGTACGATTTAGAGTAACATATAAG |  |  |
| P1071 | CTTATATGTTACTCTAAATCGTACGATGCTTGGTG | NUS0698 | *cps10AJ(F109V_E222N)* 3' |
| P54 | CATAGCCGAAGGAAGGATTGT |  |  |
| For construction of ∆*cps2J*<>*cps10AJ*(I101T_F109V) | | | |
| P51 | GAACATGGAAATGTGGAAGATGAG | NUS0697 | *cps10AJ(I101T_F109V)* 5' |
| P1068 | GCCTAGAATATAGGCGACTGGATTTTGCATAAAAG |  |  |
| P1067 | CTTTTATGCAAAATCCAGTCGCCTATATTCTAGGC | NUS0697 | *cps10AJ(I101T_F109V)* 3' |
| P54 | CATAGCCGAAGGAAGGATTGT |  |  |
| For construction of ∆*cps2J*<>*cps10AJ*(F109V/A356V) | | | |
| P51 | GAACATGGAAATGTGGAAGATGAG | NUS0698 | *cps10AJ(F109V/A356V)* 5' |
| P1074 | CACTGATAATTGCAGAAACTGTTGTTGAAATCATG |  |  |
| P1073 | CATGATTTCAACAACAGTTTCTGCAATTATCAGTG | NUS0698 | *cps10AJ(F109V/A356V)* 3' |
| P54 | CATAGCCGAAGGAAGGATTGT |  |  |
| For construction of ∆*cps2J*<>*cps10AJ*(E222N/A356V) | | | |
| P51 | GAACATGGAAATGTGGAAGATGAG | NUS0701 | *cps10AJ(E222N/A356V)* 5' |
| P1072 | CACCAAGCATCGTACGATTTAGAGTAACATATAAG |  |  |
| P1071 | CTTATATGTTACTCTAAATCGTACGATGCTTGGTG | NUS0701 | *cps10AJ(E222N/A356V)* 3' |
| P54 | CATAGCCGAAGGAAGGATTGT |  |  |
| For construction of ∆*cps2J*<>*cps10AJ*(I101T_E222N) | | | |
| P51 | GAACATGGAAATGTGGAAGATGAG | NUS0697 | *cps10AJ(I101T_E222N)* 5' |
| P1072 | CACCAAGCATCGTACGATTTAGAGTAACATATAAG |  |  |
| P1071 | CTTATATGTTACTCTAAATCGTACGATGCTTGGTG | NUS0697 | *cps10AJ(I101T_E222N)* 3' |
| P54 | CATAGCCGAAGGAAGGATTGT |  |  |
| For construction of ∆*cps2J*::*cps23BJ*(G316E) | | | |
| P1694 | ACCCGGTCATTGTTACGATTAG | NUS0141 | *cps23BJ(G316E) 5'* |
| P1974 | CTGAATAAAATGAAGCTTCGAAGAGTATATTAGAG |  |  |
| P1973 | CTCTAATATACTCTTCGAAGCTTCATTTTATTCAG | NUS0141 | *cps23BJ(G316E) 3'* |
| P1695 | CAATGGCATTACTCAGTCGTTTG |  |  |
| For construction of ∆*cps2J*<>*cps23BJ*(A152T) | | | |
| P1694 | ACCCGGTCATTGTTACGATTAG | NUS1161 | *cps2J’-cps23BJ*(*A152T*)- *cps2J’* |
| P1695 | CAATGGCATTACTCAGTCGTTTG |  |  |
| For construction of ∆*cps2J*<>*cps23BJ*(P254S) | | | |
| P1694 | ACCCGGTCATTGTTACGATTAG | NUS1162 | *cps2J’-cps23BJ*(*P254S*)- *cps2J’*) |
| P1695 | CAATGGCATTACTCAGTCGTTTG |  |  |
| For construction of ∆*cps2J*<>*cps23BJ*(F319L) | | | |
| P1694 | ACCCGGTCATTGTTACGATTAG | NUS1163 | *cps2J’-cps23BJ*(*F319L*)- *cps2J’* |
| P1695 | CAATGGCATTACTCAGTCGTTTG |  |  |
| For construction of ∆*cps2J*<> *cps23BJ*(I31T) | | | |
| P1694 | ACCCGGTCATTGTTACGATTAG | NUS1183 | *cps2J’-cps23BJ*(*I31T*)- *cps2J’* |
| P1695 | CAATGGCATTACTCAGTCGTTTG |  |  |
| For construction of ∆*cps2J*<>*cps23BJ*(T33A) | | | |
| P1694 | ACCCGGTCATTGTTACGATTAG | NUS1184 | *cps2J’-cps23BJ*(*T33A*)- *cps2J’* |
| P1695 | CAATGGCATTACTCAGTCGTTTG |  |  |
| For construction of ∆*cps2J*<>*cps23BJ*(P30S) | | | |
| P1694 | ACCCGGTCATTGTTACGATTAG | NUS1185 | *cps2J’-cps23BJ*(*P30S*)- *cps2J’* |
| P1695 | CAATGGCATTACTCAGTCGTTTG |  |  |
| For construction of ∆*cps2J*<>*cps23BJ*(L156P) | | | |
| P1694 | ACCCGGTCATTGTTACGATTAG | NUS1186 | *cps2J’-cps23BJ*(*L156P*)- *cps2J’* |
| P1695 | CAATGGCATTACTCAGTCGTTTG |  |  |
| For construction of ∆*cps2J*<>*cps23BJ*(D231G) | | | |
| P1694 | ACCCGGTCATTGTTACGATTAG | NUS1187 | *cps2J’-cps23BJ*(*D231G*)- *cps2J’* |
| P1695 | CAATGGCATTACTCAGTCGTTTG |  |  |
| For construction of ∆*cps2J*<>*cps23BJ*(WT) | | | |
| P1694 | ACCCGGTCATTGTTACGATTAG | NUS1199 | *cps2J’-cps23BJ*(*WT*)- *cps2J’* |
| P1695 | CAATGGCATTACTCAGTCGTTTG |  |  |
| For construction of ∆*cps2J*<>*cps23BJ*(P30L) | | | |
| P1694 | ACCCGGTCATTGTTACGATTAG | NUS1337 | *cps2J’-cps23BJ*(*P30L*)- *cps2J’* |
| P1695 | CAATGGCATTACTCAGTCGTTTG |  |  |
| For construction of ∆*cps2J*<>*cps23BJ*(Y41H) | | | |
| P1694 | ACCCGGTCATTGTTACGATTAG | NUS1338 | *cps2J’-cps23BJ*(*Y41H*)- *cps2J’* |
| P1695 | CAATGGCATTACTCAGTCGTTTG |  |  |
| For construction of ∆*cps2J*<>*cps23BJ*(I241T) | | | |
| P1694 | ACCCGGTCATTGTTACGATTAG | NUS1339 | *cps2J’-cps23BJ*(*I241T*)- *cps2J’* |
| P1695 | CAATGGCATTACTCAGTCGTTTG |  |  |
| For construction of ∆*cps2J*<>*cps23BJ*(F315L) | | | |
| P1694 | ACCCGGTCATTGTTACGATTAG | NUS1340 | *cps2J’-cps23BJ*(*F315L*)- *cps2J’* |
| P1695 | CAATGGCATTACTCAGTCGTTTG |  |  |
| For construction of ∆*cps2J*<>*cps23BJ*(F319S) | | | |
| P1694 | ACCCGGTCATTGTTACGATTAG | NUS1342 | *cps2J’-cps23BJ* (*F319S*)- *cps2J’* |
| P1695 | CAATGGCATTACTCAGTCGTTTG |  |  |
| For construction of ∆*cps2J*<>*cps23BJ*(V29A) | | | |
| P1694 | ACCCGGTCATTGTTACGATTAG | NUS1347 | *cps2J’-cps23BJ*(*V29A*)- *cps2J’* |
| P1695 | CAATGGCATTACTCAGTCGTTTG |  |  |
| For construction of ∆*cps2J*<>*cps23BJ*(A250V) | | | |
| P1694 | ACCCGGTCATTGTTACGATTAG | NUS1348 | *cps2J’-cps23BJ* (*A250V*)- *cps2J’* |
| P1695 | CAATGGCATTACTCAGTCGTTTG |  |  |
| For construction of ∆*cps2J*<>*cps23BJ*(S244G) | | | |
| P1694 | ACCCGGTCATTGTTACGATTAG | NUS1349 | *cps2J’-cps23BJ* (*S244G*)- *cps2J’* |
| P1695 | CAATGGCATTACTCAGTCGTTTG |  |  |

^a^ P-*kan*-*rpsL*^+^ and P-*erm* cassette were kindly provided by the laboratory of Malcolm Winkler.

1. [↑](#endnote-ref-1)
2. [↑](#endnote-ref-2)
