# Supplementary material for: High-Throughput Mutagenesis and Cross-Complementation Experiments Reveal Substrate Preference and Critical Residues of the Capsule Transporters in Streptococcus pneumoniae
Source: mBio. 2021 Nov 2;12(6):e02615-21. doi: 10.1128/mBio.02615-21 (PMC8561386; doi:10.1128/mBio.02615-21)
Supplement: TABLE S2 [file mbio.02615-21-st002.docx]

**Table S2A.** Cps33BJ does not transport lipid-linked peptidoglycan and teichoic acid precursors

| **Amplicon transformed** | **Relevant genotypes of the recipient cells** | **Transformants (CFU)** | |
| --- | --- | --- | --- |
|  |  | **-Zn^2+^** | **+Zn^2+^** |
| None | $\Delta$*cps2J*<>*cps33BJ* // P_Zn_-*cps2J* | 0 | 0 |
| $\Delta$*cps33BJ::*P-*erm* |  | 0 | >300 |
| $\Delta$*bgaA*::P-*erm* |  | >300 | >300 |
| ∆*tacF*::P-*erm* |  | - | 0 |
| ∆*ytgP*::P-*erm* |  | - | 8^b^ |
| ∆*pgdA*::P-*erm*^a^ |  | - | >300 |
| ∆*tacF*::P-*erm* | Wildtype serotype 2 D39 | - | 0 |
| ∆*ytgP*::P-*erm* |  | - | 10^b^ |
| ∆*pgdA*::P-*erm*^a^ |  | - | >300 |
| ∆*tacF*::P-*erm* | ∆*cps2J*<>*ytgP* // P_Zn_*-cps2J* | - | 0 |
| ∆*ytgP*::P-*erm* |  | - | >300 |
| ∆*pgdA*::P-*erm*^a^ |  | - | >300 |
| ∆*tacF*::P-*erm* | ∆*cps2J*<>*tacF* // P_Zn_*-cps2J* | - | >300 |
| ∆*ytgP*::P-*erm* |  | - | 0 |
| ∆*pgdA*::P-*erm*^a^ |  | - | >300 |

"-" denote that transformation reaction was not tested.

^a^ *pgdA* is an unrelated gene and ∆*pgdA*::P-*erm* was used as a transformation efficiency control.

^b^ Diagnostic PCR were performed and showed that the corresponding amplicons were not introduced in these isolates.

**Table S2B.** Cps10AJ* variants transport the cognate precursor

| **Genotype of recipient cells** | **Transformants obtained when the indicated amplicon was introduced (CFU)** | |
| --- | --- | --- |
|  | None | ∆*cps10AJ*::P-*erm* |
| *rpsL1* CPS10A | - | 0 |
| *rpsL1* CPS10A *∆bgaA*::P-*kan*-*cps10AJ^+^* | 0 | >300 |
| *rpsL1* CPS10A ∆*bgaA*::P-*kan-cps10AJ(I101T)* | 0 | >300 |
| *rpsL1* CPS10A ∆*bgaA*::P-*kan-cps10AJ(F109V)* | 0 | >300 |
| *rpsL1* CPS10A ∆*bgaA*::P-*kan-cps10AJ(Y192H)* | 0 | >300 |
| *rpsL1* CPS10A ∆*bgaA*::P-*kan-cps10AJ(E222N)* | 0 | >300 |
| *rpsL1* CPS10A ∆*bgaA*::P-*kan-cps10AJ(A346V)* | 0 | >300 |

"-" denote that transformation reaction was not tested.

**Table S2C.** Cps10BJ does not transport lipid-linked peptidoglycan and teichoic acid precursors

| **Amplicon transformed** | **Relevant genotypes of the recipient cells** | **Transformants (CFU)** |
| --- | --- | --- |
| None | *rpsL1* ∆*cps2J<>cps10BJ* ∆*bgaA*::P_Zn_-*cps2J* | 0 |
| $\Delta$*pgdA::*P*-erm*^a^ | *rpsL1* ∆*cps2J<>cps10BJ* ∆*bgaA*::P_Zn_-*cps2J* | >300 |
| $\Delta$*ytgP::*P*-erm* | *rpsL1* ∆*cps2J*<>*ytgP* ∆*bgaA*::P_Zn_-*cps2J* | >300 |
|  | *rpsL1* ∆*cps2J<>cps10BJ* ∆*bgaA*::P_Zn_-*cps2J* | 4^b^ |
| $\Delta$*tacF::*P*-erm* | *rpsL1* ∆*cps2J*<>*tacF* ∆*bgaA*::P_Zn_-*cps2J* | >300 |
|  | *rpsL1* ∆*cps2J<>cps10BJ* ∆*bgaA*::P_Zn_-*cps2J* | 2^b^ |

^a^ *pgdA* is an unrelated gene and ∆*pgdA*::P-*erm* was used as a transformation efficiency control.

^b^ Diagnostic PCR were performed and showed that the corresponding amplicons were not introduced in these isolates.

**Table S2D.** Cps23BJ* variants transport the cognate precursor

| **Genotype of recipient cells** | **Transformants obtained when the indicated amplicon was introduced (CFU)** | |
| --- | --- | --- |
|  | None | ∆*cps23BJ*::P-*erm* |
| *rpsL1* CPS23B | - | 0 |
| *rpsL1* CPS23B *∆bgaA*::P-*kan*-*cps23BJ^+^* | 0 | >300 |
| *rpsL1 CPS23B* ∆*bgaA*::P-*kan-cps23BJ(P30S)* | - | >300 |
| *rpsL1 CPS23B* ∆*bgaA*::P-*kan-cps23BJ(T33A)* | - | >300 |
| *rpsL1 CPS23B* ∆*bgaA*::P-*kan-cps23BJ(A152T)* | - | >300 |
| *rpsL1* CPS23B ∆*bgaA*::P-*kan-cps23BJ(S244G)* | - | >300 |
| *rpsL1* CPS23B ∆*bgaA*::P-*kan-cps23BJ(D231G)* | - | >300 |
| *rpsL1* CPS23B ∆*bgaA*::P-*kan-cps23BJ(P254S)* | - | >300 |
| *rpsL1* CPS23B ∆*bgaA*::P-*kan-cps23BJ(G316E)* | - | >300 |

"-" denote that transformation reaction was not tested.

**Table S2E.** Cps23BJ* variants do not transport lipid-linked peptidoglycan and teichoic acid precursors

| **Genotype of recipient cells** | **Transformants obtained when the indicated amplicon was introduced (CFU)** | | | |
| --- | --- | --- | --- | --- |
|  | None | ∆*pgdA*:: P-*erm*^a^ | ∆*ytgP*:: P-*erm* | ∆*tacF*:: P-*erm* |
| *rpsL1* ∆*cps2J*<>*tacF* ∆*bgaA*::P_Zn_-*cps2J* | - | - | - | >300 |
| *rpsL1* ∆*cps2J*<>*ytgP* ∆*bgaA*::P_Zn_-*cps2J* | - | - | >300 | - |
| *rpsL1* ∆*cps2J*<>*cps23BJ ∆bgaA*::P_Zn_-*cps2J* | 0 | >300 | 0 | 0 |
| *rpsL1* ∆*cps2J*<>*cps23BJ(V29A) ∆bgaA*::P_Zn_-*cps2J* | 0 | >300 | 4^b^ | 0 |
| *rpsL1* ∆*cps2J*<>*cps23BJ(P30L) ∆bgaA*::P_Zn_-*cps2J* | 0 | >300 | 0 | 0 |
| *rpsL1* ∆*cps2J*<>*cps23BJ(P30S) ∆bgaA*::P_Zn_-*cps2J* | 0 | >300 | 0 | 0 |
| *rpsL1* ∆*cps2J*<>*cps23BJ(I31T) ∆bgaA*::P_Zn_-*cps2J* | 0 | >300 | 0 | 0 |
| *rpsL1* ∆*cps2J*<>*cps23BJ(T33A) ∆bgaA*::P_Zn_-*cps2J* | 0 | >300 | 1^b^ | 0 |
| *rpsL1* ∆*cps2J*<>*cps23BJ(Y41H) ∆bgaA*::P_Zn_-*cps2J* | 0 | >300 | 0 | 0 |
| *rpsL1* ∆*cps2J*<>*cps23BJ(A152T) ∆bgaA*::P_Zn_-*cps2J* | 0 | >300 | 0 | 0 |
| *rpsL1* ∆*cps2J*<>*cps23BJ(L156P) ∆bgaA*::P_Zn_-*cps2J* | 0 | >300 | 0 | 0 |
| *rpsL1* ∆*cps2J*<>*cps23BJ(D231G) ∆bgaA*::P_Zn_-*cps2J* | 0 | >300 | 0 | 0 |
| *rpsL1* ∆*cps2J*<>*cps23BJ(I241T) ∆bgaA*::P_Zn_-*cps2J* | 0 | >300 | 0 | 0 |
| *rpsL1* ∆*cps2J*<>*cps23BJ(S244G) ∆bgaA*::P_Zn_-*cps2J* | 0 | >300 | 0 | 0 |
| *rpsL1* ∆*cps2J*<>*cps23BJ(A250V) ∆bgaA*::P_Zn_-*cps2J* | 0 | >300 | 0 | 0 |
| *rpsL1* ∆*cps2J*<>*cps23BJ(P254S) ∆bgaA*::P_Zn_-*cps2J* | 1 | >300 | 0 | 0 |
| *rpsL1* ∆*cps2J*<>*cps23BJ(F315L) ∆bgaA*::P_Zn_-*cps2J* | 0 | >300 | 0 | 0 |
| *rpsL1* ∆*cps2J*<>*cps23BJ(G316E) ∆bgaA*::P_Zn_-*cps2J* | 0 | >300 | 0 | 0 |
| *rpsL1* ∆*cps2J*<>*cps23BJ(F319L) ∆bgaA*::P_Zn_-*cps2J* | 0 | >300 | 1^b^ | 2^b^ |
| *rpsL1* ∆*cps2J*<>*cps23BJ(F319S) ∆bgaA*::P_Zn_-*cps2J* | 0 | >300 | 0 | 1^b^ |

"-" denote that transformation reaction was not tested.

^a^ *pgdA* is an unrelated gene and ∆*pgdA*::P-*erm* was used as a transformation efficiency control.

^b^ Diagnostic PCR were performed and showed that the corresponding amplicons were not introduced in these isolates.
