# Supplementary material for: High-Throughput Mutagenesis and Cross-Complementation Experiments Reveal Substrate Preference and Critical Residues of the Capsule Transporters in Streptococcus pneumoniae
Source: mBio. 2021 Nov 2;12(6):e02615-21. doi: 10.1128/mBio.02615-21 (PMC8561386; doi:10.1128/mBio.02615-21)

A

```
Serotype10A    MKVLKNYAYNLSYQLLLIILPIITTPYVTRVFSLNDLGTYGYFNSIVTYFILLATLGVAN    60
Serotype10B    MKVLKNYAYNLSYQLLLIILPIITTPYVTRVFSLNDLGTYGYFNSIVTYFILLATLGVAN    60
               ************************************************************

Serotype10A    YGTKVISGHRKEIEKNFWGIYSLQLGATVLSMFLYLVLCLILPFMQNPFAYILGLSLVSK    120
Serotype10B    YGTKVISGHRKEIEKNFWGIYSLQLGATVLSMFLYLVLCLTLPFMQNPVAYILGLSLVSK    120
               **************************************** *******.***********

Serotype10A    GLDISWLFQGLEDFRKITARNIIVKLVGVSSIFLFIKSASDLYLYVFLLTIFELLGQLSM    180
Serotype10B    GLDISWLFQGLEDFRKITARNIIVKLVGVSSIFLFIKSASDLYLYVFLLTIFELLGQLSM    180
               ************************************************************

Serotype10A    WLPAREFIGKPYFDLSYAKQHLKPIVLLFLPQVAISLYVTLERTMLGALSSTKDVGIYDQ    240
Serotype10B    WLPAREFIGKPHFDLSYAKQHLKPIVLLFLPQVAISLYVTLNRTMLGALSSTKDVGIYDQ    240
               ***********:*****************************:******************

Serotype10A    ALKLVNILLTLVTSLGSVMLPRVANLLATGDYKAVNKMHEMSFLIYNLVIFPIMAGILIV    300
Serotype10B    ALKLVNILLTLVTSLGSVMLPRVANLLATGDYKAVNKMHEMSFLIYNLVIFPIMAGILIV    300
               ************************************************************

Serotype10A    NDDFVQFFLGQDFQDARYAIAIMIFRMFFIGWTNIMGIQILIPHNKNKEFMISTTASAII    360
Serotype10B    NDDFVQFFLGQDFQDARYAIAIMIFRMFFIGWTNIMGIQILIPHNKNKEFMISTTVSAII    360
               *******************************************************.****

Serotype10A    SVGLNMLFLPKLGYIGAAIVSVLTEALVWAIQLFYTRRYLKEVPIIVSMTKIVLASVIMY    420
Serotype10B    SVGLNMLFLPKLGYIGAAIVSVLTEALVWAIQLFYTRRYLKEVPIIVSMTKIVLASVIMY    420
               ************************************************************

Serotype10A    GILLGSKIFIHFSPTINVLAFAVLGGIIYLFAILSMKVIDVKELKQIIKK*    470
Serotype10B    GILLGSKIFIHFSPTINVLAFAVLGGIIYLFAILSMKVIDVKELKQIIKK*    470
               **************************************************
```

B

Number	Position	Codon (amino acid) Cps10AJ	Codon (amino acid) Cps10BJ
1	93	TTT (Phe)	TTC (Phe)
2	101	ATT (Ile)	ACT (Thr)
3	109	TTC (Phe)	GTC (Val)
4	165	TAC (Tyr)	TAT (Tyr)
5	192	TAT (Tyr)	CAT (His)
6	222	GAA (Glu)	AAT (Asn)
7	356	GCT (Ala)	GTT (Val)

C

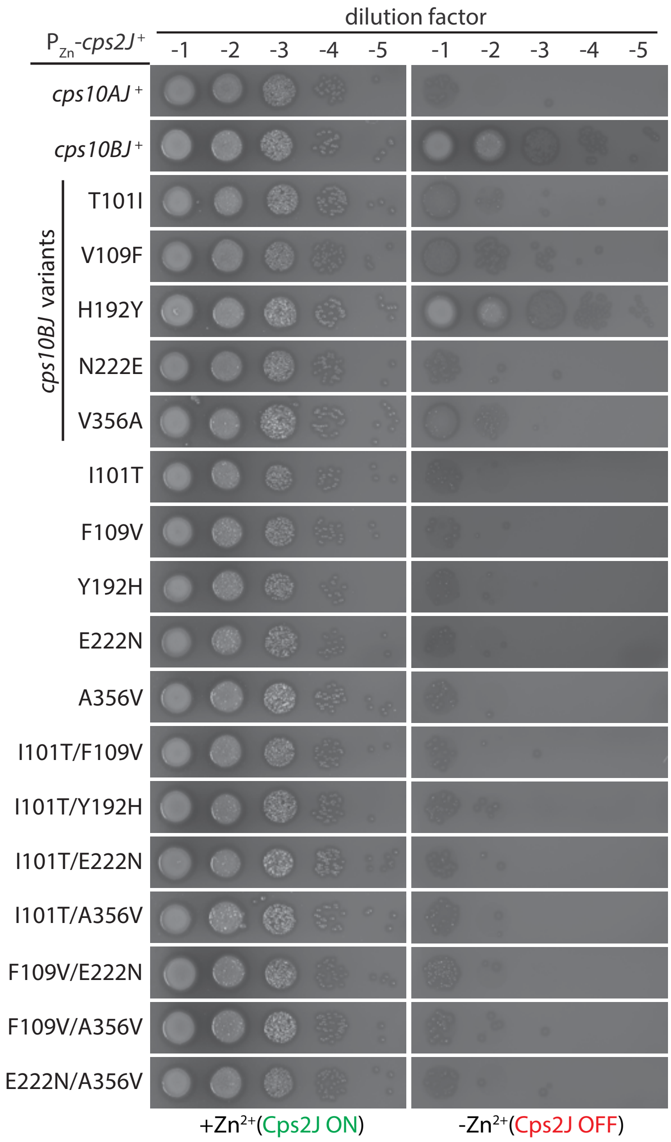

D

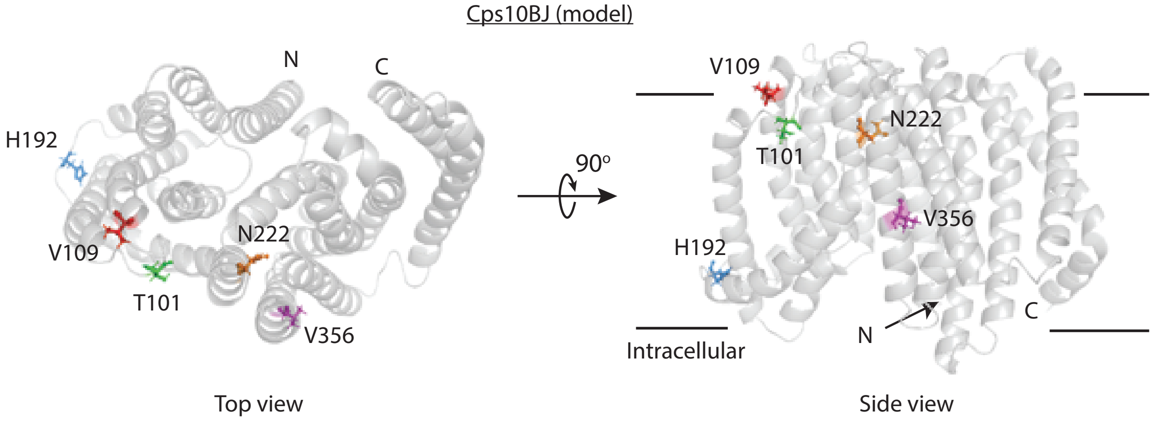

E

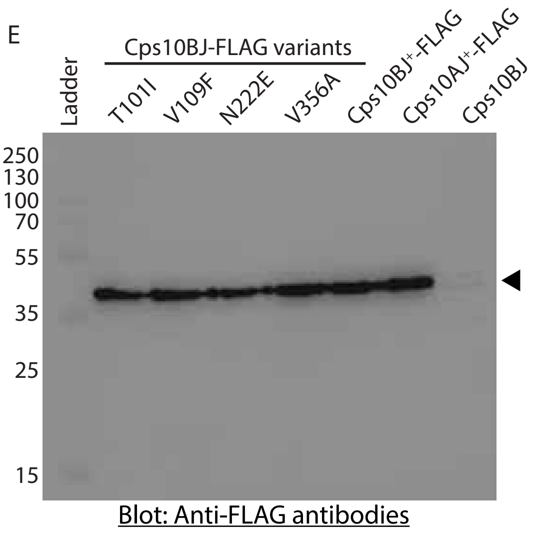

F

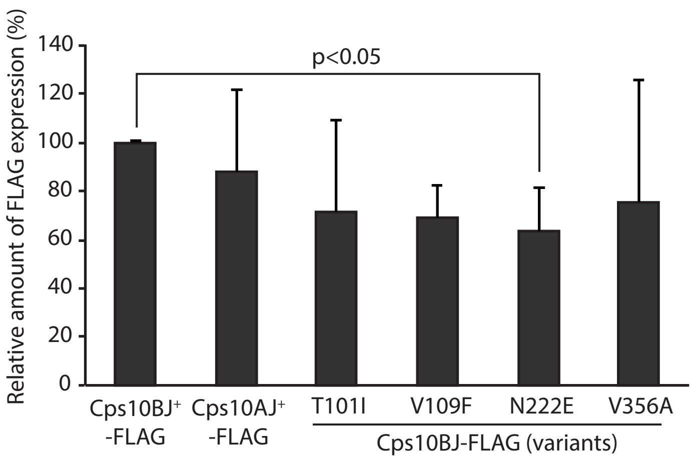

Supplement: FIG S4 [file mbio.02615-21-sf004.pdf]
